# A computational model of spatio-temporal cardiac intracellular calcium handling with realistic structure and spatial flux distribution from sarcoplasmic reticulum and t-tubule reconstructions

**DOI:** 10.1371/journal.pcbi.1005714

**Published:** 2017-08-31

**Authors:** Michael A. Colman, Christian Pinali, Andrew W. Trafford, Henggui Zhang, Ashraf Kitmitto

**Affiliations:** 1 School of Biomedical Sciences, Faculty of Biological Sciences, University of Leeds, Leeds, United Kingdom; 2 School of Physics and Astronomy, Faculty of Engineering and Physical Sciences, University of Manchester, Manchester, United Kingdom; 3 Division of Cardiovascular Sciences, Faculty of Biology, Medicine and Health Sciences, University of Manchester, Manchester, United Kingdom; University of Virginia, UNITED STATES

## Abstract

Intracellular calcium cycling is a vital component of cardiac excitation-contraction coupling. The key structures responsible for controlling calcium dynamics are the cell membrane (comprising the surface sarcolemma and transverse-tubules), the intracellular calcium store (the sarcoplasmic reticulum), and the co-localisation of these two structures to form dyads within which calcium-induced-calcium-release occurs. The organisation of these structures tightly controls intracellular calcium dynamics. In this study, we present a computational model of intracellular calcium cycling in three-dimensions (3-D), which incorporates high resolution reconstructions of these key regulatory structures, attained through imaging of tissue taken from the sheep left ventricle using serial block face scanning electron microscopy. An approach was developed to model the sarcoplasmic reticulum structure at the whole-cell scale, by reducing its full 3-D structure to a 3-D network of one-dimensional strands. The model reproduces intracellular calcium dynamics during control pacing and reveals the high-resolution 3-D spatial structure of calcium gradients and intracellular fluxes in both the cytoplasm and sarcoplasmic reticulum. We also demonstrated the capability of the model to reproduce potentially pro-arrhythmic dynamics under perturbed conditions, pertaining to calcium-transient alternans and spontaneous release events. Comparison with idealised cell models emphasised the importance of structure in determining calcium gradients and controlling the spatial dynamics associated with calcium-transient alternans, wherein the probabilistic nature of dyad activation and recruitment was constrained. The model was further used to highlight the criticality in calcium spark propagation in relation to inter-dyad distances. The model presented provides a powerful tool for future investigation of structure-function relationships underlying physiological and pathophysiological intracellular calcium handling phenomena at the whole-cell. The approach allows for the first time direct integration of high-resolution images of 3-D intracellular structures with models of calcium cycling, presenting the possibility to directly assess the functional impact of structural remodelling at the cellular scale.

## Author summary

The organisation of the membrane and sub-cellular structures of cells in the heart closely controls the coupling between its electrical and mechanical function. Computational models of the cellular calcium handling system, which is responsible for this electro-mechanical coupling, have been developed in recent years to study underlying structure-function relationships. Previous models have been largely idealised in structure; we present a new model which incorporates experimental data describing the high-resolution organisation of the primary structures involved in calcium dynamics. Significantly, the structure of the intracellular calcium store is modelled for the first time. The model is shown to reproduce calcium dynamics in control cells in both normal and abnormal conditions, demonstrating its suitability for future investigation of structure-function relationships. Thus, the model presented provides a powerful tool for the direct integration of experimentally acquired structural data in healthy and diseased cells and assessment of the role of structure in regulating normal and abnormal calcium dynamics.

## Introduction

The cardiac intracellular calcium (Ca^2+^) handling system is responsible for the control of cellular and organ contraction associated with the heartbeat [1]. Malfunction of this system can directly affect the ability of the heart to work effectively as a pump–reducing cardiac output and potentially leading to mortality. Moreover, abnormal Ca^2+^ handling dynamics has been increasingly linked to the development of arrhythmogenic triggers in the myocardium [2–4], through two-way coupling between the electrical and Ca^2+^ handling systems.

Ca^2+^ handling in cardiac myocytes is regulated by multiple ion channels, pumps and transporters. During the cellular electrical action potential (AP) associated with excitation, an influx of Ca^2+^ through opening of the voltage-gated L-type Ca^2+^ channels (LTCCs—carrying flux *J*_*CaL*_) triggers a significant release of Ca^2+^ from the intracellular Ca^2+^ store (the sarcoplasmic reticulum, SR) through opening of the Ryanodine Receptors (RyRs—carrying flux *J*_*rel*_). This process is referred to as Ca^2+^-induced- Ca^2+^-release (CICR) [5]. The Ca^2+^ released from the SR binds with the contractile myofilaments in the bulk intracellular space—the cytoplasm—facilitating cellular contraction. During relaxation, the Ca^2+^ concentration in the SR is restored from the cytoplasm through the flux *J*_*up*_, carried by the SERCA Ca^2+^ pump; Ca^2+^ is removed from the cell primarily by the sodium- Ca^2+^-exchanger (NCX–carrying flux *J*_*NaCa*_) and also through the membrane Ca^2+^ pump (PMCA—carrying flux *J*_*pCa*_). This completes the cardiac cellular Ca^2+^ cycle associated with electrical activation and contraction.

The LTCCs are found in clusters along the surface membrane and transverse-tubules (TTs)—invaginations of the sarcolemmal membrane responsible for delivering the AP into the interior of the cell. PMCA and NCX are distributed on the surface sarcolemmal membrane and the TTs. On the SR membrane there are clusters of RyRs [6] and continuously distributed SERCA proteins. Critical for CICR is the spatial arrangement of the TT network and the junctional portion of the SR (jSR) to form a microdomain, termed the dyad, to co-localise LTCCs and RyRs on the two membranes. There are thousands of dyads within a cell, and the bulk of the SR forms a network like structure (nSR) connecting the distributed jSRs.

Employing serial block face scanning electron microscopy (SBF-SEM) we have previously determined the 3-D organisation of the TTs and SR in a large animal model, the sheep, revealing details of the network organisation and jSR morphology and importantly the relationship with the TT network to form dyads [7]. Multiple groups have demonstrated that the TTs and related structures are remodelled in disease conditions [7–14] and we have additionally shown that the SR structure is also perturbed in heart failure [7]. These observations highlight the potential importance of structure-function relationships at the sub-cellular scale in Ca^2+^ dynamics associated with cardiac arrhythmia and perturbed contraction; the precise nature and impact of these relationships requires further investigation.

Computational modelling is a complementary approach to experimental research, and has been successfully applied to provide insight into numerous cardiac phenomena, such as pacemaker activity (e.g., [15]) and the functional impact of pathophysiological ion channel remodelling (e.g., [16]). Recently, computational models which explicitly account for spatio-temporal Ca^2+^ dynamics have been developed and successfully reproduced phenomena including Ca^2+^ transient alternans and spontaneous Ca^2+^ release [17–41]. Such models are in general idealised (with an idealised structure and dyad distribution), but some have used imaging data to distribute RyRs throughout the cell or cell-portion [17,18,22,39]; Other models have been created of small regions of the cell (e.g. the volume surrounding a single TT or even a single dyad) and have integrated more detailed imaging data [29,31,32,37]. However, a spatio-temporal Ca^2+^ handling model which accounts for realistic SR structure, TT structure and dyad distribution at the whole-cell scale has not yet been developed and involves significant challenges. Nevertheless, the potential advantages offered by such a model–providing a method to directly study variability in structure-function relationships–remain an attractive prospect.

Therefore, the aim of this study was to develop an approach to overcome the challenges of modelling spatio-temporal Ca^2+^ dynamics using the experimentally reconstructed 3-D structures for the TT and SR at the whole-cell scale. We demonstrate that the approaches developed are sufficient to capture spatio-temporal calcium dynamics with a realistic network SR structure and membrane fluxes distributed according to the sarcolemma/TTs. We additionally report how this model can be used to reproduce Ca^2+^ transient alternans and spontaneous release events during perturbed rapid pacing as a demonstration of its suitability for future research, and provide preliminary analysis of the importance of structure-function relationships underlying cardiac cellular dynamics. This model (provided in S1 Code) therefore provides a powerful tool to understand structure-function relationships in physiological and pathophysiological cardiac electro-mechanics.

## Methods

**Fig 1:**
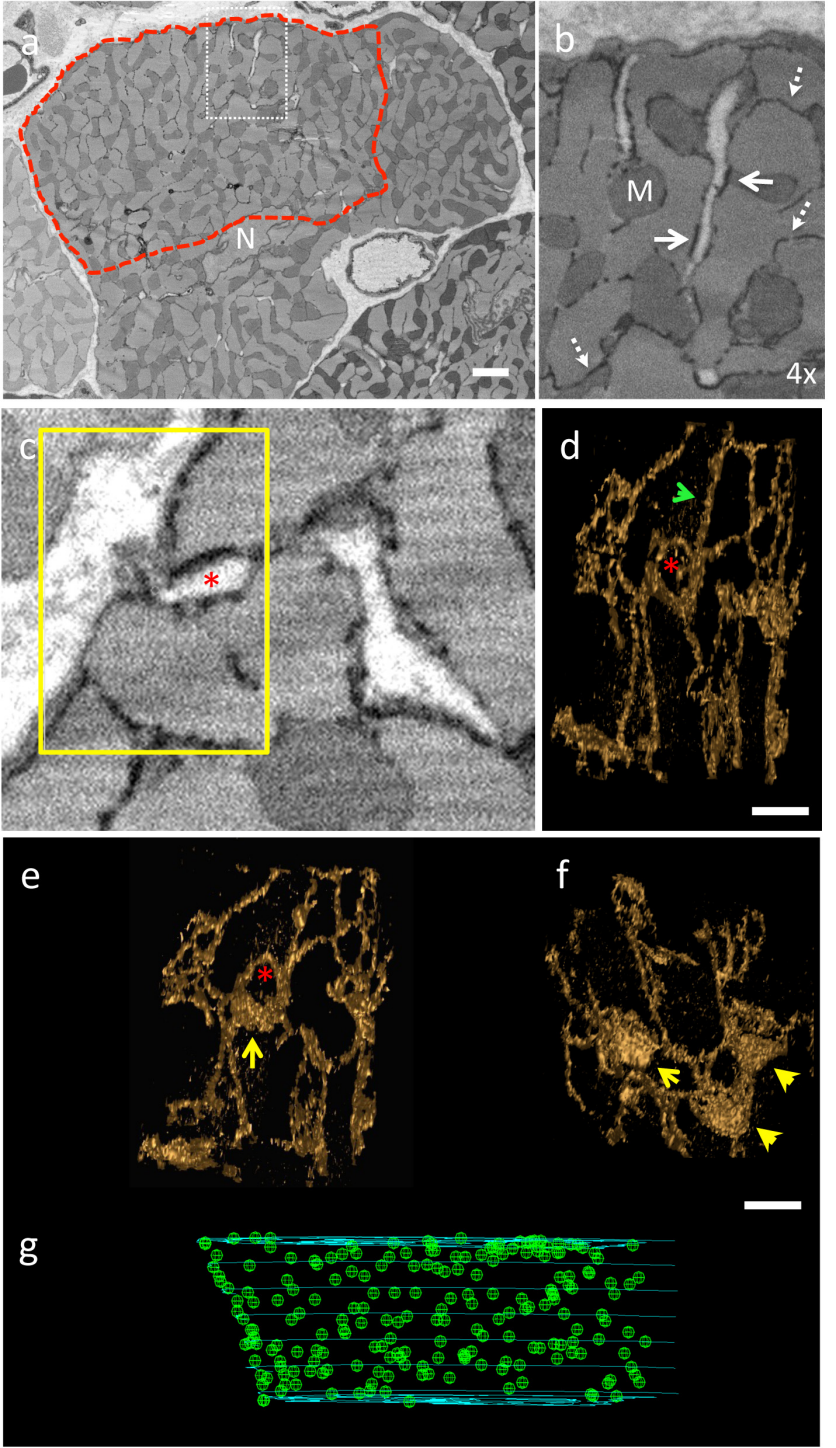
The application of serial block face scanning electron microscopy to analyse the distribution of the dyads, sites of Ca^2+^ release, within a cardiac myocyte. (a) Exemplar portion of a serial image taken from an SBF-SEM stack showing a cardiac myocyte in cross-section. The t-tubules are white (no stain uptake) and the SR is black (electron dense). N = nucleus. The region enclosed by the dashed red line highlights the portion of the cell through which the jSR (dyads) were mapped. Scale bar (in white) = 2μm. The boxed region (white dash) is magnified in panel (b) to illustrate the SR morphology, black density, (dashed arrows) and the relationship with the t-tubules to form dyads (white arrows). M = mitochondria (c) The region highlighted by the yellow box indicates an exemplar region through which the SR 3-D structure has been determined to illustrate how it is formed from network SR and jSR. (d) 3-D structure of the SR isosurface (gold), rotated to display where the SR circles a t-tubule (not shown for clarity but position indicated by red asterisk). An example of network SR is indicated by a green arrowhead. Scale bar = 500 nm (e) Rotation of the 3-D structure shown in (d) to illustrate that the portion of SR wrapped around the t-tubule is a jSR patch (yellow arrow) (f) Reconstruction of the SR deeper into the image stack (Z-direction) reveals several other jSR patches (yellow arrowheads). Scale bar = 500 nm (g) Shows a portion of the volume through which the positions of the jSR patches (each jSR indicated by a green sphere) have been identified and mapped within the region encompassed by the red boundary shown in panel (a). The positions of jSR were identified through 357 images (17.85 μm) in the Z-direction.

The development of a computational model to achieve the goal of simulating spatio-temporal Ca^2+^ dynamics with realistic structure and fluxes requires multiple steps: first, the selected image-based dataset describing structure of the TTs and nSR (Fig 1) must be processed to (a) identify and segment the dyads and (b) construct geometry grids suitable for numerical simulation; second, an idealised model for spatio-temporal Ca^2+^ dynamics must be updated to include these new structures. A major feature of this approach is processing of the SR reconstruction for simulation.

### Identifying the location of each dyad within a portion of a cardiac myocyte

Details of image acquisition using SBF-SEM (FEI Quanta 250 FEG SEM equipped with a Gatan 3View ultramicrotome) and methods for the 3-D reconstruction of the TT and SR structure have been described fully elsewhere [7]. In brief, tissue was extracted from the sheep left ventricle and immediately fixed and prepared for SBF-SEM [42]. The voxel size of the acquired data was 13.5 nm /pixel in the x-y plane and 50 nm in the z-direction. The region within a cardiac myocyte for which the dyad positional data was generated for this study is indicated in Fig 1A. The staining technique employed allowed clear delineation of the TT and SR features (Fig 1A–1C). Segmentation of the SR subsequently allowed the network SR (nSR) to be distinguished from the jSR ‘patches’. 3-D reconstruction of both the TTs and SR morphology ([43]) within the defined region enabled identification of jSR along TTs forming putative dyads (Fig 1D–1F). The position of each dyad was marked with a sphere (Fig 1) to build up a 3-D distribution grid through 357 consecutive slices.

### Reconstructing intracellular structure for numerical simulation

The resolution at which the images were acquired, 13.5nm ×13.5nm × 50nm_,_ is impractical for computational modelling at the whole-cell scale. Computational grids created for numerical simulation were down-sampled to a resolution of 350nm × 350nm × 350nm following processing at the full resolution.

#### Bulk cytoplasm geometry

**Fig 2:**
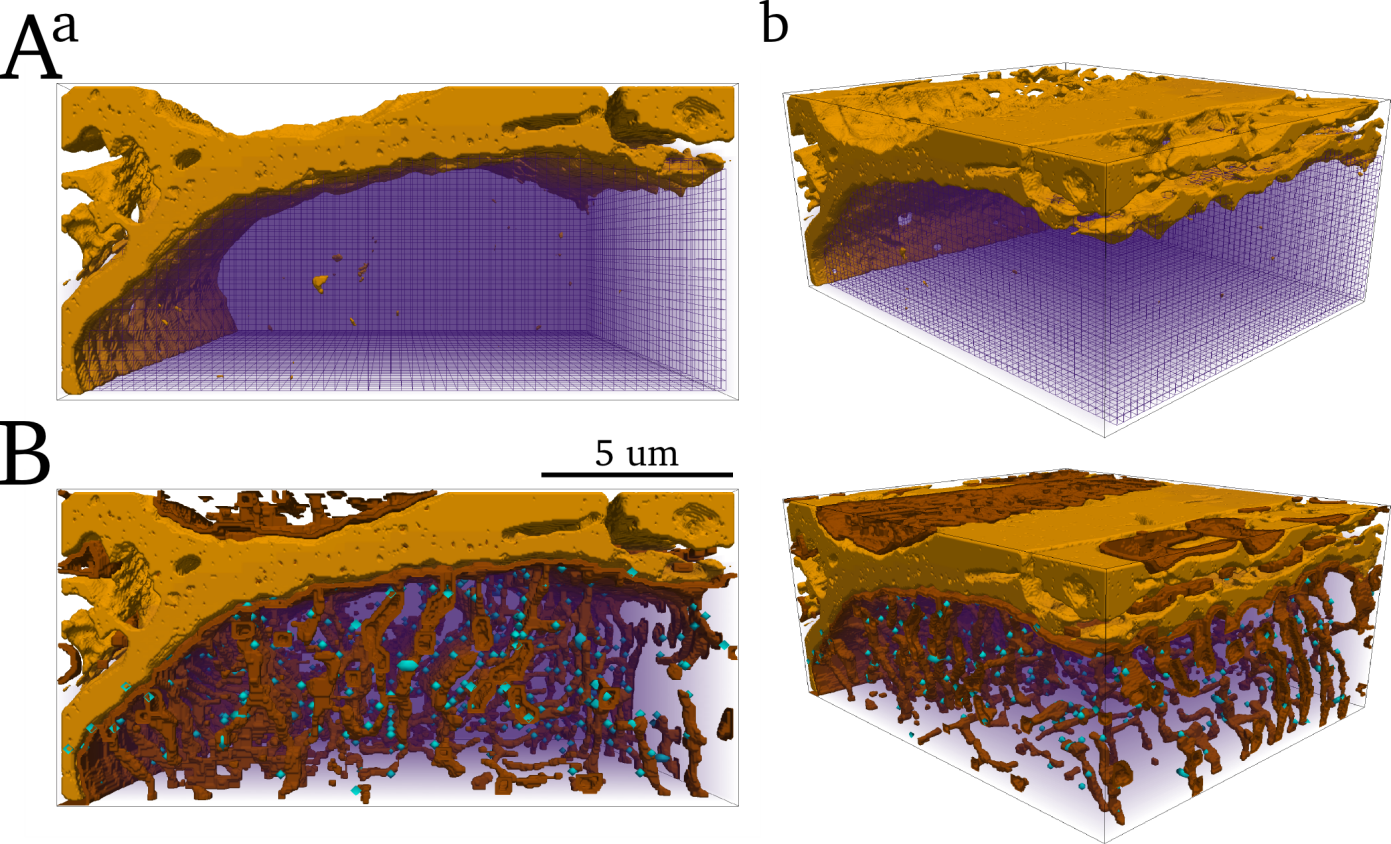
Reconstruction of a portion of the cytoplasm and TT structure for computational modelling. **A)** The CYTO grid (wireframe and purple volume render) enclosed by the isolated surface sarcolemma (light brown contour). **B)** Smoothed and interpolated reconstruction of the TTs (dark brown contour) and dyad locations (light blue contour dots). **a)** is a view along the longitudinal axis of the cell and **b)** is at an angle.

The surface sarcolemma was first isolated from the TTs. The volume enclosed by the surface sarcolemma and the grid boundaries was down-sampled to form the computational grid describing the bulk intracellular space (Fig 2A). The volume of a single voxel in the cytoplasm is given as ~75% of the volume at the discretised resolution, i.e., 0.0314 μm^3^, accounting for the volume occupied by other intracellular structures such as the mitochondria and myofilaments.

#### Surface sarcolemma and t-tubule membranes

The outer surface of the cytoplasm grid was designated as the surface sarcolemma. The reconstructed TT geometry was smoothed and cleaned to create a continuous TT-network (Fig 2B). Voxels of the cytoplasm grid associated with the surface sarcolemma and TTs form the surface-sarcolemma and TT maps (SS_M and TT_M, respectively). The co-ordinates of the reconstructed dyads were then transformed to the appropriate locations at the lower resolution grid to define the location of the dyads.

### Reconstructing the SR for numerical simulation

**Fig 3:**
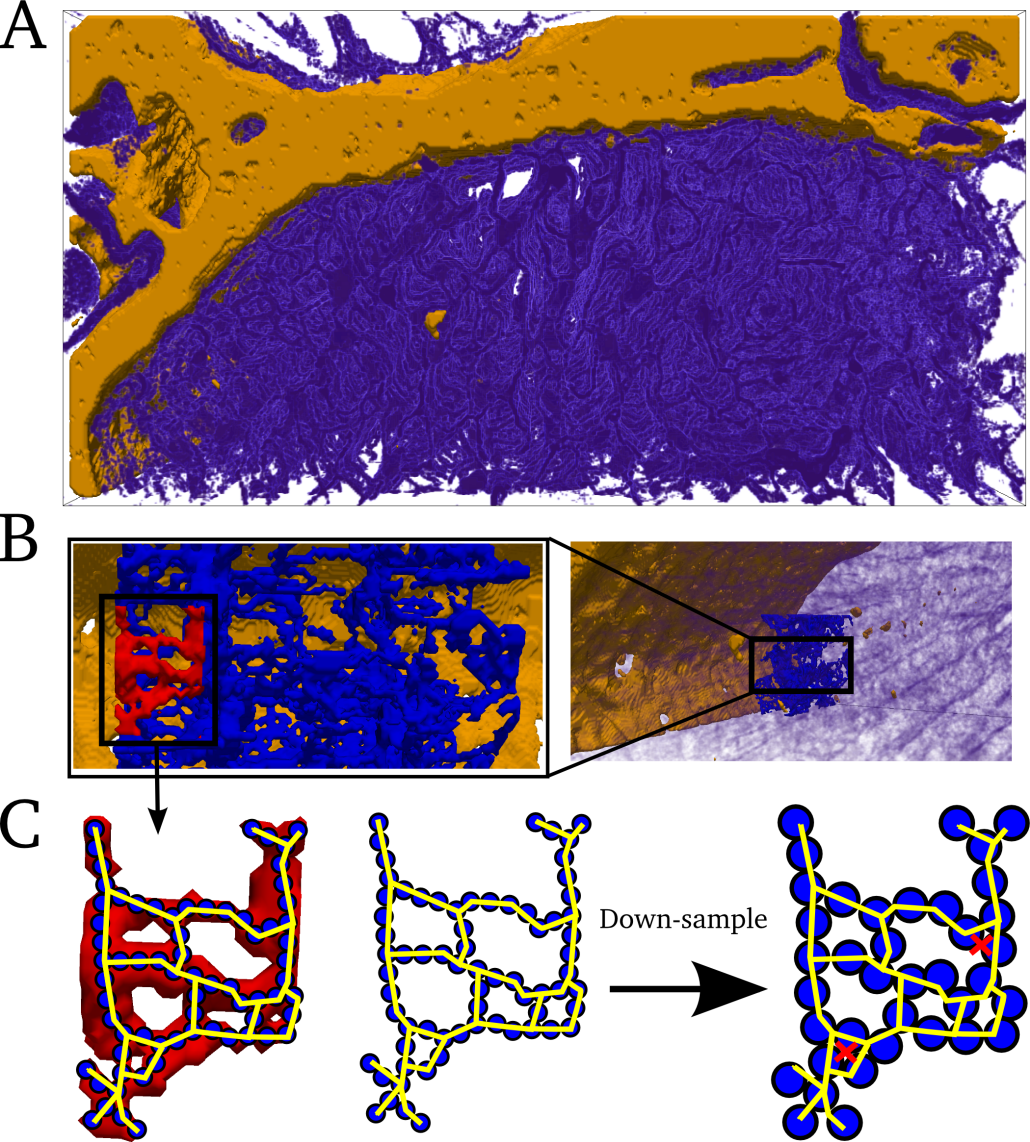
Reconstruction of the SR for computational modelling. **A**) Smoothed reconstruction of the SR at full resolution, shown in purple volume render with the surface sarcolemma shown in light brown for reference. **B**) Zoomed view of a small portion of the SR (blue contour) and smaller extracted portion (red contour). **C)** Illustration of the 1-D strand map (yellow) which is described by nodes (blue circles) at the original resolution (left and centre panels) and the down-sampled resolution (right panel). Spatial coupling occurs only along the strand map; adjacent nodes at the down-sampled resolution are only coupled through the neighbourhood map described by the strand structure–the red crosses in the right panel indicate nodes which are spatially adjacent but not coupled.

First, the reconstruction of the SR at full resolution was smoothed and cleaned (Fig 3A). This was the baseline geometry to which the down-sampled geometry was mapped. The primary challenge in modelling SR structure is due to its thin cross section: resolutions necessary to capture detailed SR structure are impractical for whole-cell simulations.

To overcome this challenge, the 3-D network structure of the SR, rather than its full 3-D cross-sectional structure, was identified as the key feature to be captured in the model. Under this reduction, the SR can be approximated by a 3-D network of 1-D strands (Fig 3C). The resolution of this “1-D-strand model” was down-sampled to 350nm × 350nm × 350nm, and an algorithm applied to preserve each SR voxel’s nearest-neighbours (Fig 3C).

The volume of each node in the 1-D strand model corresponds to the total volume of the full resolution reconstructed SR divided by the number of nodes in the 1-D strand map, and is thus not defined by the volume of a single voxel at the discretised resolution (*v*_*vox*.*nsr*_ = 0.00305 μm^3^). For visualization of small portions of the cell model, the concentration distribution in the 1-D strand model was mapped back onto the full resolution reconstruction; this was impractical for visualization of the whole cell due to the computational memory demands of rendering such a large, high resolution structure.

### Development of the computational model

In this section, a general spatio-temporal Ca^2+^ handling model is described in the context of an idealised cell structure. Then, approaches to incorporating the reconstructed SR and membrane structures are discussed. Full model equations can be found in S1 Text; in this section, only the fundamental equations are given.

#### Basic spatio-temporal Ca^2+^ cycling model

**Fig 4:**
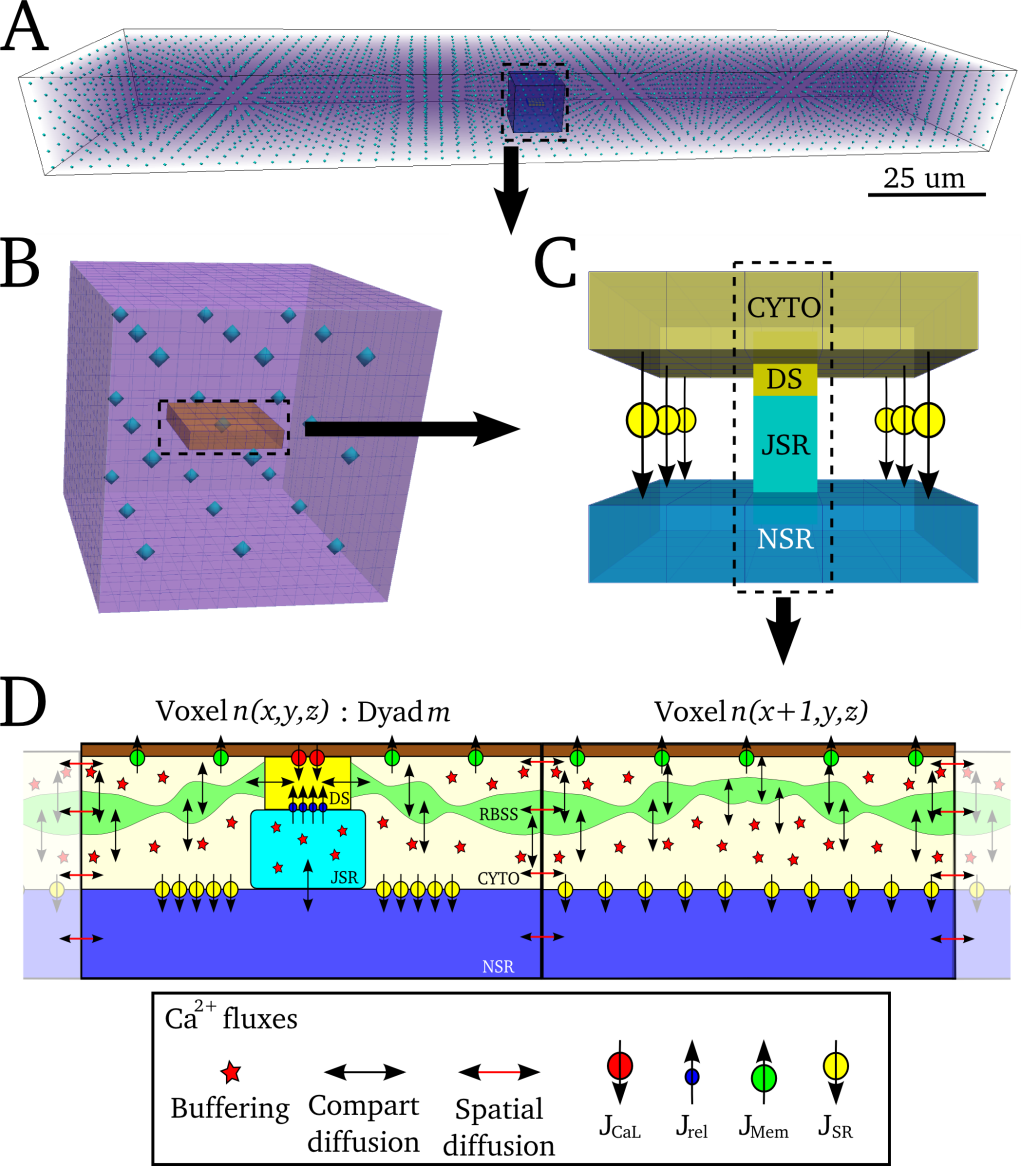
Schematic structure of idealised 3-D spatio-temporal Ca^2+^ handling model. **A)** Whole cell grid (purple volume view), with voxels corresponding to dyads shown by the blue contour dots. **B)** Enlarged view of a small portion of the whole cell grid containing 3x3x3 dyads, corresponding to the highlighted portion shown in (**A**). A 5x5 voxel slice containing a single dyad at the centre is shown in orange. **C)** Illustration of the cytoplasm and nSR domains contained within the 5x5 slice in (**B**), with the dyadic cleft and jSR shown. Flux J_SR_ is illustrated between corresponding voxels of the CYTO and NSR grids. **D**) Schematic of two voxels of each domain, with one containing a dyad. The RBSS and CYTO compartments of the CYTO grid are shown. Continuous Ca^2+^ diffusion occurs within the CYTO, RBSS and NSR grids. DS refers to the dyadic cleft space.

Model structure: The general model of intracellular Ca^2+^ cycling in an idealised geometry is similar to those previously published by other groups [17–23]. First, an idealised cellular geometry grid (in this case, an oblong or cylinder) was created which describes Ca^2+^ concentration in the bulk cytoplasm and nSR (Fig 4A). The bulk cytoplasm also contains a restricted buffering subspace (rbSS, Fig 4D), functionally equivalent to the direct coupling between dyads used in previous studies [26,28]. Ca^2+^ diffusion occurs continuously throughout the grid for each of these three domains. Voxels at regular intervals were selected to correspond to the dyads (Fig 4A–4C). The fluxes coupling the three domains and the dyads are shown schematically in Fig 4C and 4D.

Ca^2+^ dynamics in the bulk intracellular and nSR spaces: Ca^2+^ dynamics in the three diffusively coupled domains is described by the isotropic reaction-diffusion equation:

$$\frac{d{\left[Ca^{2+}\right]}_{cyto}}{dt}=\beta_{cyto}\left(D\nabla^{2}{\left[Ca^{2+}\right]}_{cyto}+\phi_{cyto}\right)$$ (1)

$$\frac{d{\left[Ca^{2+}\right]}_{nSR}}{dt}=\left(D\nabla^{2}{\left[Ca^{2+}\right]}_{nSR}+\phi_{nSR}\right)$$ (2)

$$\frac{d{\left[Ca^{2+}\right]}_{rbSS}}{dt}=\left(D\nabla^{2}{\left[Ca^{2+}\right]}_{rbSS}+\phi_{rbSS}\right)$$ (3)

Where *Φ* is a general reaction term, defined in the following paragraphs, *β*_*cyto*_ is the instantaneous buffering term [44], given in S1 Text, *∇*^*2*^ is the spatial Laplacian operator in 3-D and ***D*** is the diffusion coefficient. At each voxel, *n*, the Laplacian is approximated by the 6 node nearest neighbour finite difference method.

In the bulk cytoplasm (i.e., not at a dyad location), three primary fluxes contribute to the reaction term: the membrane Ca^2+^ current fluxes (*J*_*Mem*_ = *J*_*NaCa*_+ *J*_*pCa*_+ *J*_*Cab*_); intracellular uptake into the SR (*J*_*SR*_ = *J*_*up*_-*J*_*leak*_); and diffusion between the cytoplasm and rbSS spaces (*J*_*ss*_), as well as troponin buffering (*J*_*trpn*_). In the bulk nSR, only *J*_*SR*_ contributes to the reaction. For the idealised cell model, *J*_*Mem*_ and *J*_*SR*_ are solved continuously throughout the voxel grid. Thus, the reaction, *Φ*, at each voxel *n* is given by:

$${}^{n}\phi_{cyto}={}^{n}J_{NaCa}+{}^{n}J_{pCa}+{}^{n}J_{Cab}-({}^{n}J_{up}-{}^{n}J_{leak})+\left(\frac{v_{rbSS\_vox}}{v_{cyto\_vox}}\right){}^{n}J_{SS}-{}^{n}J_{trpn}$$ (4)

$${}^{n}\phi_{nSR}=\left({}^{n}J_{up}-{}^{n}J_{leak}\right)\left(\frac{v_{cyto\_vox}}{v_{nSR\_vox}}\right)$$ (5)

$${}^{n}\phi_{rbSS}={-}^{n}J_{SS}$$ (6)

Where

$${}^{n}J_{SS}=\left({}^{n}\left[Ca^{2+}\right]{}_{rbSS}-{}^{n}\left[Ca^{2+}\right]{}_{cyto}\right)\tau_{ss}^{-1}$$ (7)

and where *v*_*cyto*,*rbSS*,*nSR_vox*_ is the volume of a single voxel of each of the three domains, the preceding superscript *n* denotes the individual voxels (*n = 1*, *2 … N*_*vox*_) and ^n^*J*_*up*_, ^n^*J*_*NaCa*_, ^n^*J*_*Cab*_, ^n^*J*_*pCa*,_^n^*J*_*trpn*_ are solved for local Ca^2+^ concentrations at voxel *n*.

Ca^2+^ dynamics at dyad locations: For voxels corresponding to the location of dyads, the reaction term for the subspace–to which the dyad is coupled (Fig 4)—was updated to include diffusion from dyadic cleft, *J*_*ds*_, and the reaction term for the nSR was updated to include diffusion into the jSR, *J*_*jSR*_. Mapping functions associate dyad *m (m = 1*, *2 … N*_*dyads*_*)* with voxel *n*, defined as the dyad map, *θ(m) = n*, and inverse map, *θ* ^*-1*^*(n) = m*. Thus, for all voxels *n* within the set *θ(m)*:

$${}^{n}\phi_{rbSS}={-}^{n}J_{SS}+{}^{\theta^{-1}(n)}J_{ds}\left(\frac{v_{ds}}{v_{rbSS\_vox}}\right)$$ (8)

$${}^{n}\phi_{nSR}=\left({}^{n}J_{up}-{}^{n}J_{leak}\right)\left(\frac{v_{cyto\_vox}}{v_{nSR\_vox}}\right){+}^{\theta^{-1}(n)}J_{jSR}\left(\frac{v_{jSR}}{v_{nSR\_vox}}\right)$$ (9)

$${}^{m}J_{ds}=\left({}^{m}\left[Ca^{2+}\right]{}_{ds}-{}^{\theta (m)}\left[Ca^{2+}\right]{}_{rbSS}\right)\tau_{ds}^{-1}$$ (10)

$${}^{m}J_{jSR}=\left({}^{m}\left[Ca^{2+}\right]{}_{jSR}-{}^{\theta (m)}\left[Ca^{2+}\right]{}_{nSR}\right)\tau_{jSR}^{-1}$$ (11)

Ca^2+^ dynamics in the dyadic cleft and jSR are described by:

$$\frac{d{}^{m}\left[Ca^{2+}\right]{}_{ds}}{dt}=\beta \left({}^{m}J_{rel}+{}^{m}J_{CaL}-{}^{m}J_{ds}\right)$$ (12)

$$\frac{d{}^{m}\left[Ca^{2+}\right]{}_{jSR}}{dt}=\beta \left(-{}^{m}J_{rel}\left(\frac{v_{ds}}{v_{jSR}}\right)-{}^{m}J_{jSR}\right)$$ (13)

Where *v*_*ds*,*jSR*_ is the volume of a single dyad/jSR and *J*_*rel*_ and *J*_*CaL*_ are a function of the RyRs and LTCCs, respectively. Both of these are described by stochastic-differential-equation Markov chain models and solved by the standard Monte-Carlo method at the individual channel level (with random numbers generated by the Mersenne Twister [45]). The channel numbers for the RyRs and LTCCs can be varied heterogeneously throughout the cell; for simplicity (and due to a lack of specific data describing the distribution of these channels), in the present study these channels were homogeneously distributed between dyads, where the number of RyRs and LTCCs per dyad is 100 and 15, respectively [20].

Due to the small volume of the dyadic cleft, an analytical description can be found for the dyadic cleft Ca^2+^ concentration under the approximation that within a small time-interval (*Δt* = 0.01ms) the volume rapidly reaches its steady state resultant from the fluxes acting into it [20,46]. Thus, by setting:

$$\frac{d{\left[Ca^{2+}\right]}_{ds}}{dt}=0$$ (14)

An approximation for Eq (10) can be obtained as:

$${}^{m}\left[Ca^{2+}\right]{}_{ds}≃{}^{\theta (m)}\left[Ca^{2+}\right]{}_{rbSS}+\frac{\tau_{ds}.\left({}^{m}k{}_{rel}.{}^{m}\left[Ca^{2+}\right]{}_{jSR}+{}^{m}J_{CaL}\right)}{\left(1+\tau_{ds}.{}^{m}k{}_{rel}\right)}$$ (15)

$${}^{m}k{}_{rel}={}^{m}n{}_{o\_RyR}.g_{RyR}.{}^{m}v{}_{ds}^{-1}$$ (16)

Where *n*_*O_RyR*_ is the number of open RyR channels in the dyad (described by the RyR Markov chain model) and *g*_*RyR*_ is the maximal flux rate through the RyRs. We note that this approximation yields the same results as numerical integration, at reduced computational cost.

Troponin buffering and active force development: Active force is calculated in each voxel from the local troponin levels according to a modified whole-cell model developed in previous studies [47,48].

Parameter values and further equations are given in S1 Text.

#### Incorporating processed structure datasets

Updating the model to include the processed reconstructions of the SR and membrane systems involves: (1) defining the geometry of the cytoplasm and subspace; (2) coupling between the nSR and cytoplasm spaces; (3) distributing the membrane fluxes according to the reconstructed surface sarcolemma and TTs.

The reconstructed bulk intracellular space (Fig 2A) forms the geometry of the bulk cytoplasm and the subspace. Because the 1-D-strand model of the nSR is discretised at the same resolution as the cytoplasm, a direct map between the two structures can be created, wherein SR voxel *q* (*q = 1*, *2 … N*_*SR*_) is associated with cytoplasm voxel *n* by the mapping function *θ*_*SR*_*(q) = n* and inverse map *θ*_*SR*_^*-1*^*(n) = q*. Similarly, the surface sarcolemma and TT maps (SS_M, TT_M–see Methods: Reconstructing intracellular structure for numerical simulation) are also discretised at the same resolution and mapping functions were created to relate membrane voxel *p* (*p = 1*, *2 … N*_*mem*_) with cytoplasm voxel *n* (*θ*_*mem*_*(p) = n* and inverse map *θ*_*mem*_^*-1*^*(n) = p*). Eq (4) is thus updated to solve the reaction terms only at voxels defined by the mapping functions:

$$\begin{array}{l} {}^{n}\phi_{cyto}{=}^{n}J_{SS}{-}^{n}J_{trpn}\\ {}^{n}\phi_{cyto}=({}^{q}J_{up}-{}^{q}J_{leak}){+}^{n}J_{SS}{-}^{n}J_{trpn}\\ {}^{n}\phi_{cyto}={}^{p}J_{NaCa}+{}^{p}J_{pCa}+{}^{p}J_{Cab}{+}^{n}J_{SS}{-}^{n}J_{trpn}\\ {}^{n}\phi_{cyto}={}^{p}J_{NaCa}+{}^{p}J_{pCa}+{}^{p}J_{Cab}-({}^{q}J_{up}-{}^{q}J_{leak}){+}^{n}J_{SS}{-}^{n}J_{trpn} \end{array}\}\begin{array}{c} \forall n\notin \theta_{mem}(p)\wedge n\notin \theta_{SR}(q)\\ \forall n\notin \theta_{mem}(p)\wedge n\in \theta_{SR}(q)\\ \forall n\in \theta_{mem}(p)\wedge n\notin \theta_{SR}(q)\\ \forall n\in \theta_{mem}(p)\wedge n\in \theta_{SR}(q) \end{array}$$ (17)

And Eqs (5) and (6) are also updated similarly:

$$\begin{array}{l} {}^{q}\phi_{nSR}=\left({}^{q}J_{up}-{}^{q}J_{leak}\right)\\ {}^{q}\phi_{nSR}=\left({}^{q}J_{up}-{}^{q}J_{leak}\right)+{}^{m}J_{jSR} \end{array}\}\begin{array}{c} \forall q\notin \theta (m)\\ \forall q\in \theta (m) \end{array}$$ (18)

$$\begin{array}{l} {}^{n}\phi_{rbSS}={-}^{n}J_{SS}\\ {}^{n}\phi_{rbSS}={-}^{n}J_{SS}+{}^{m}J_{ds} \end{array}\}\begin{array}{c} \forall n\notin \theta (m)\\ \forall n\in \theta (m) \end{array}$$ (19)

Where volume balances have been implicitly incorporated for clarity and *p* and *q* are given by the inverse mapping functions of *n*.

Heterogeneity in the membrane and SR fluxes can be easily incorporated by scaling the fluxes in individual voxels. Moreover, the distribution of membrane currents between the surface sarcolemma and TTs can be controlled by adjusting the flux rates relative to the number of voxels associated with each sarcolemmal membrane map. In the present study, for simplicity and to ensure that any emergent heterogeneity in Ca^2+^ concentration is a result only of structure, heterogeneity in protein expression was not included and the membrane current fluxes were evenly distributed between the two membranes (except where explicitly stated otherwise).

#### Coupling to an ionic model

The focus of this study is the development of a new spatio-temporal Ca^2+^ handling model, and therefore a simplified ionic model was implemented to describe the AP. Key currents (*I*_*Na*_, *I*_*NaL*_, *I*_*Kr*_, *I*_*Ks*_, *I*_*K1*_, *I*_*NaK*_, *I*_*b*_) were extracted from the O’hara-Rudy Human Ventricle model [49]. Small modifications to the conductance of some ion currents (S1 Text) were made to reflect the difference between human and sheep AP characteristics [50,51] and to account for the model simplification. Local fluxes from the intracellular model (*J*_*CaL*_, *J*_*NaCa*_, *J*_*pCa*_, *J*_*Cab*_) are converted into currents through:

$$I_{NaCa,pCa,Cab}=C_{m}^{-1}z.\sum_{p}\left({}^{p}J_{NaCa,pCa,Cab}.F.v_{vox}\right)$$ (20)

$$I_{CaL}=C_{m}^{-1}z.\sum_{m}\left({}^{m}J_{CaL}.F.v_{ds}\right)$$ (21)

Where *v*_*vox*_ and *v*_*ds*_ are the volume of a single voxel of the cytoplasm and dyadic cleft space, respectively, *z* is the valence associated with the current, *F* is the faraday constant, and the sums over *p* and *m* refer to the total voxels associated with the membrane and dyad maps, respectively. In models of a portion of the whole cell, the currents were scaled to the whole cell by the factor N_tot_dyads_/N_dyads_.

This gives the differential equation describing the membrane potential, *V*_*m*_, as:

$$\frac{dV_{m}}{dt}=-\left(I_{Na}+I_{NaL}+I_{to}+I_{Kr}+I_{Ks}+I_{K1}+I_{NaK}+I_{b}+I_{CaL}+I_{NaCa}+I_{pCa}+I_{Cab}\right)/C_{m}$$ (22)

#### Creating a whole cell model

The focus of the present study is the methodological development and therefore performing a detailed reconstruction of the full cell was considered beyond its scope and unnecessary. Instead, a full cell geometry was created from the reconstructed portion by tessellating the reconstructed geometries (cytoplasm and nSR) and associated mapping functions.

**Fig 5:**
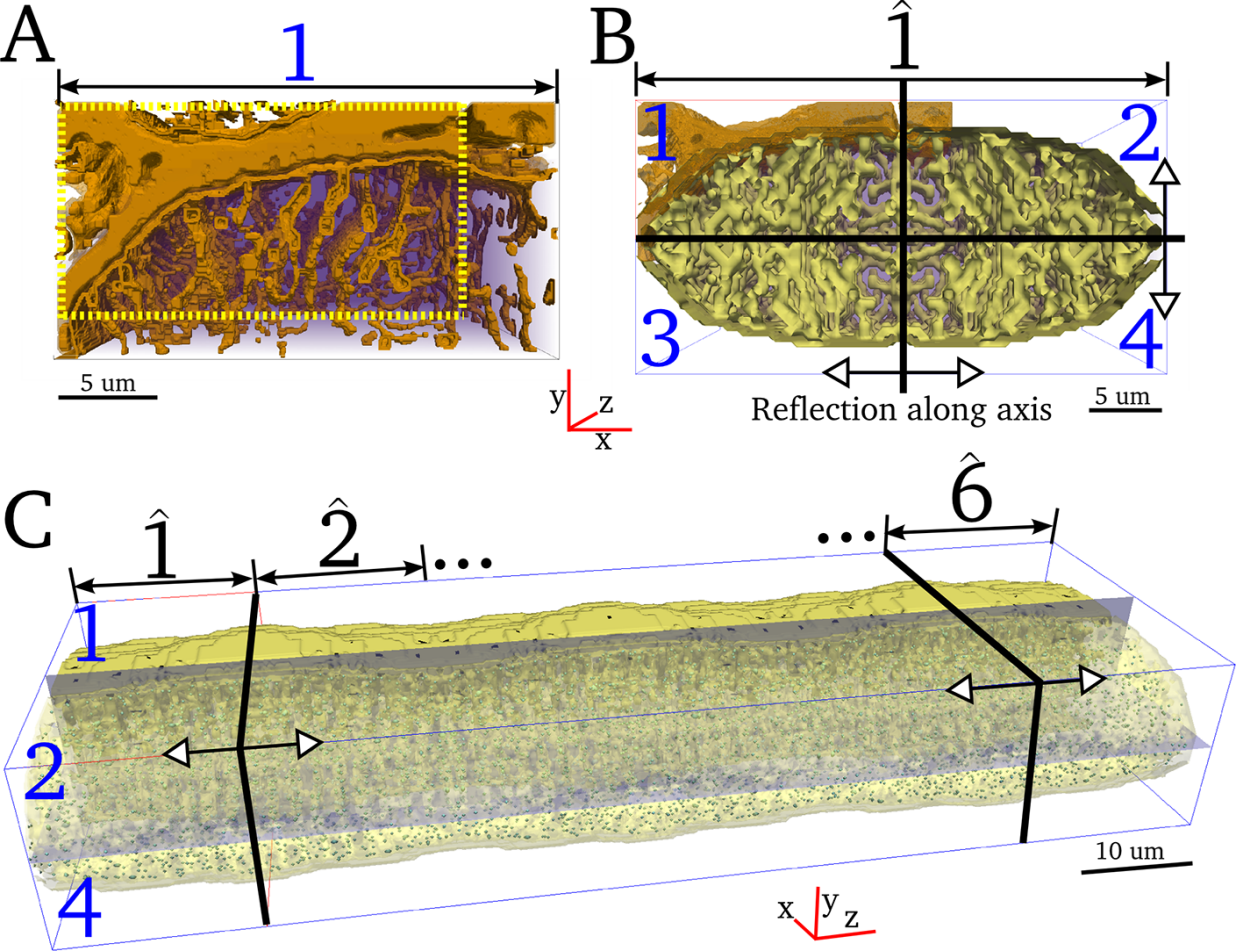
Creating a whole cell geometry. **A)** Portion of the cell which has been reconstructed at full resolution, with the full resolution TTs and surface membranes shown in brown contour. View is of the x-y plane with the z axis into the page. Cropped quarter-portion is shown within the yellow dotted box. **B)** Full cross-section of the cell, created by reflecting the cropped portion (1) in the y-z plane (creating 2) and then the x-z plane (creating 3 and 4). Yellow contour is the reduced resolution membrane map in the cytoplasm. The symmetry of the reflected structure is clear. **C)** Full cell length reconstruction. The whole cross section is reflected in the x-y plane 6–8 times. Dyads are shown as contour dots, and slices are shown to illustrate the cross-sectional axes (x-z and y-z planes).

First, the available portion (dimension 60 × 31 × 52 voxels) was cropped such that it corresponds to a quarter portion of the cross-section, dimension 50 × 26 × 52 voxels (Fig 5A). The quarter portion was then reflected along each cross-sectional axis to create a full cross-section of the cell of length 18 μm, dimension 100 × 52 × 52 voxels (Fig 5B). The cross section was reflected along the longitudinal axis 6–8 times to create cell models accounting for a range of cell lengths (Fig 5C). The dimensions of these full cell models are 100 × 52 × 312–416 voxels = 35μm ×18μm × 109–145μm, containing 13,094–17,458 dyads.

Comparison of behaviour in a single cross-sectional portion of the cell (length 18μm) with the full length cell model demonstrates that the overall dynamics are comparable between these two models, with the cross-sectional portion cell model exhibiting a larger degree of noise than the full cell model due to the smaller dyad numbers (S1 Fig). Therefore, the cross-sectional portion model of the cell is suitable for long run simulations and pre-pacing and is significantly more computationally efficient than the full length cell model.

### Semi-idealised and perturbed structure models

In order to perform preliminary analysis to assess the role of intracellular structure affecting spatio-temporal Ca^2+^ dynamics, alternative geometries were considered for comparison to the fully detailed structural model: (1) Semi-idealised structure: The cytoplasm geometry was used to describe all of the spatially diffuse spaces and fluxes. Dyads were distributed evenly throughout the volume to match the mean inter-dyad distances in the structurally detailed model, resulting in comparable dyad densities (*N*_*dyads*_ for cross section = 2182 vs 2208 for the full and semi-idealised models, respectively). (2) Altered dyad densities: The density of the dyad distribution (and thus inter-dyad distances) in the fully detailed structural model was altered, by either including additional dyads (at junctions of the SR and TT) or by removing dyads. (3) Altered SR diffusion properties: The diffusion coefficient in the SR was varied (by a factor of half and a factor of two). Furthermore, the connectivity of the SR neighbour map was perturbed by removing some neighbours for a randomly selected set of the SR voxels.

## Results

### Whole cell characteristics

**Fig 6:**
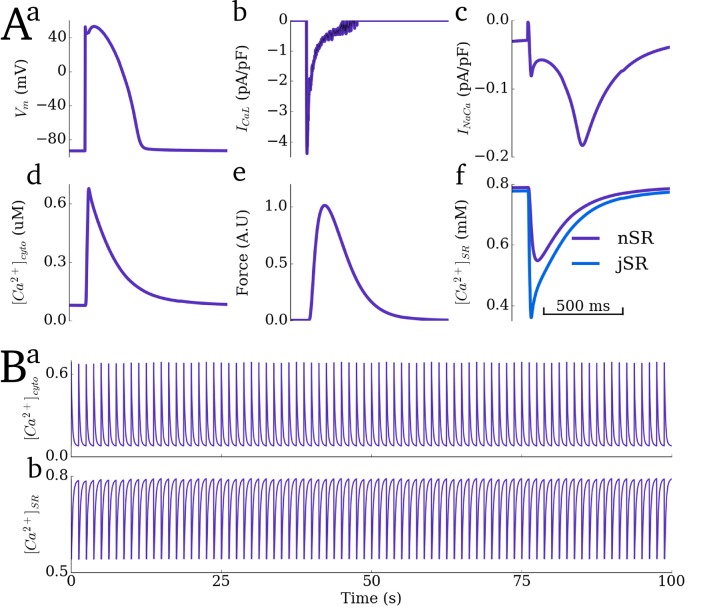
Whole-cell average characteristics during control pacing. **A**) Single beat dynamics for: **a)** membrane potential (*V*_*m*_); **b)** L-type Ca^2+^ current, *I*_*CaL*_; **c)** sodium-Ca^2+^ exchanger current, *I*_*NaCa*_; **d)** average intracellular Ca^2+^ transient; **e)** normalised contractile force; **f)** average Ca^2+^ concentration in the network (purple) and junctional (blue) SR. **B**) long term stability of the dynamic Ca^2+^ concentrations in the cytoplasm (**a**) and SR (**b**). Basic cycle length (BCL) for all panels is 1250ms.

The developed cell model reproduces properties of whole cell electrical and Ca^2+^ handling dynamics (Fig 6A) during control pacing (basic cycle length, BCL, of 1250ms). The AP duration to 90% repolarisation (APD_90_) at this cycle length is 345ms and the Ca^2+^ transient has an upstroke time of 20ms, magnitude of 0.68μM and duration to 90% of peak of 400ms. These properties fall within expected ranges for large mammal ventricular myocytes (e.g. for the sheep–the animal model from which the structural datasets were attained [50,51]). The temporal evolution of force follows the Ca^2+^ transient (Fig 6Ae) which in turn follows the AP upstroke. Model dynamics are stable over long simulation duration times (Fig 6B) once steady-state is achieved. The model successfully reproduces rate dependence of the diastolic and systolic Ca^2+^ concentrations, exhibiting an elevation of both as pacing rate increases (S2 Fig).

### Spatio-temporal Ca^2+^ dynamics in the bulk cytoplasm

**Fig 7:**
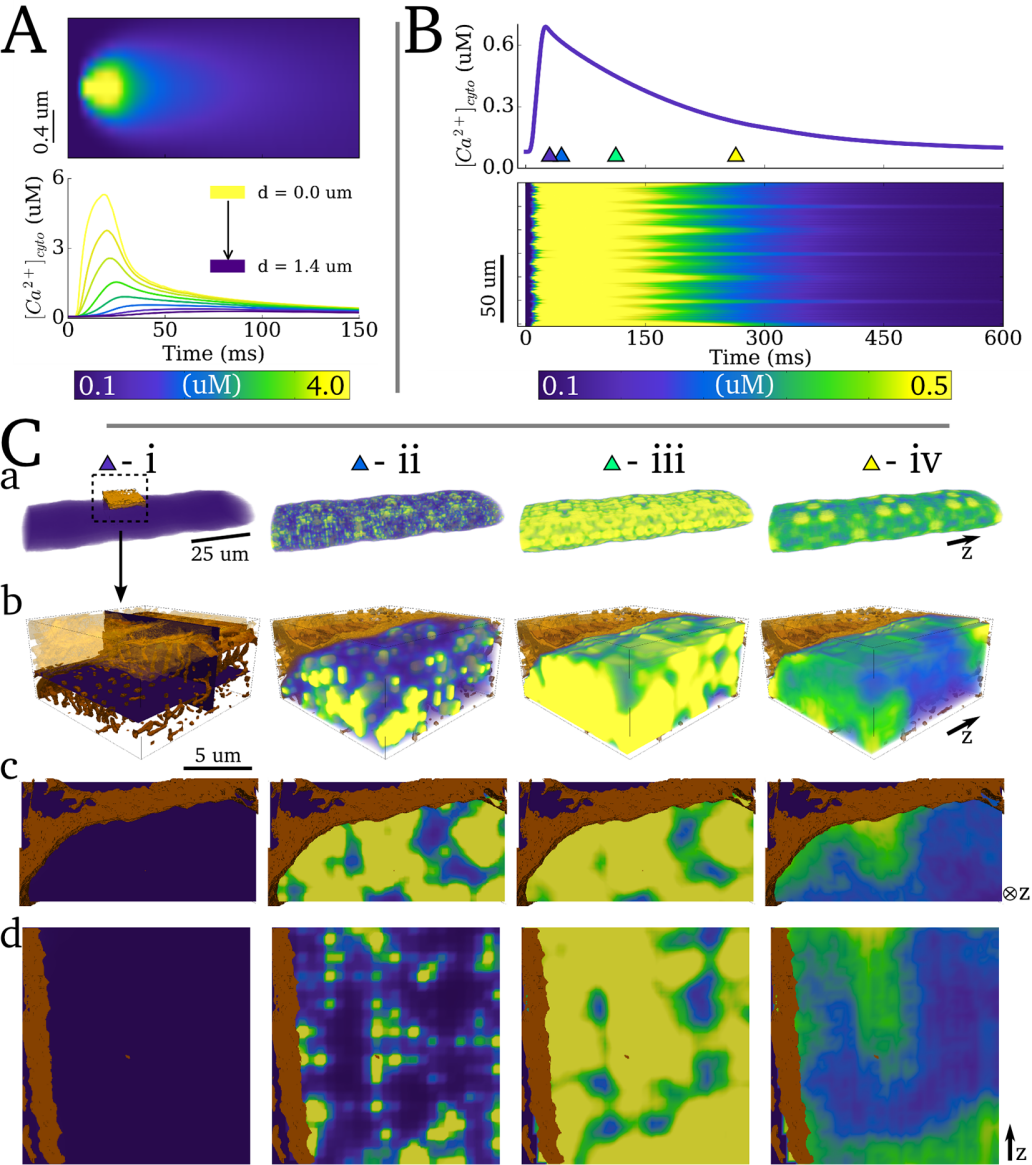
Spatio-temporal Ca^2+^ dynamics in the cytoplasm. **A)** Linescan of a single Ca^2+^ spark (upper panel) and Ca^2+^ transients at different distances from the dyad centre (lower panel) **B)** Whole-cell intracellular Ca^2+^ transient (upper panel) and linescan along the longitudinal axis for a single beat (lower panel). **C)** Temporal snapshots of spatial Ca^2+^ concentration in a 3-D volume of the cell from multiple views: **a)** whole cell; **b)** small portion of the cell (1/4 cross section; 1/6 length), corresponding to the highlighted location in (**ai**); **c)** cross-sectional 2D slice and; **d)** 2D slice along the longitudinal axis. The slices in (**c**) and (**d**) are illustrated in bi. Triangular markers in (**A**) indicate the timings of the corresponding panels (**Ci-iv**)**–** 0ms, 10ms, 50ms and 200ms relative to the applied stimulus. The purple-yellow colour bar corresponds to all spatial plots. The original reconstructed sarcolemma and TTs (brown contours) are shown for reference. Note that the repeated symmetry in Ca^2+^ concentration structure is a direct result of the whole-cell geometry being created by tessellating a small portion of the cell (see Methods: Creating a whole cell model).

Spatio-temporal dynamics in the cytoplasmic Ca^2+^ concentration associated with a single beat are shown in Fig 7 and S1 Video. The model captures the dynamics of a single Ca^2+^ spark (Fig 7A), illustrating the rapid and non-linear decay of the Ca^2+^ transient as a function of distance from the centre of the dyad [17,28]: the peak of the Ca^2+^ concentration at a distance of 1.2 μm from the centre of the dyad was an order of magnitude smaller than at the dyad centre.

A linescan along the longitudinal axis of the cell demonstrates the spatial variation in the rise and decay of the Ca^2+^ transient (Fig 7B): temporal variation of the initiation of the upstroke of the transient results in significant spatial gradients during the initial phase of excitation; significantly smoother spatial gradients were observed during the decay phase.

**Fig 8:**
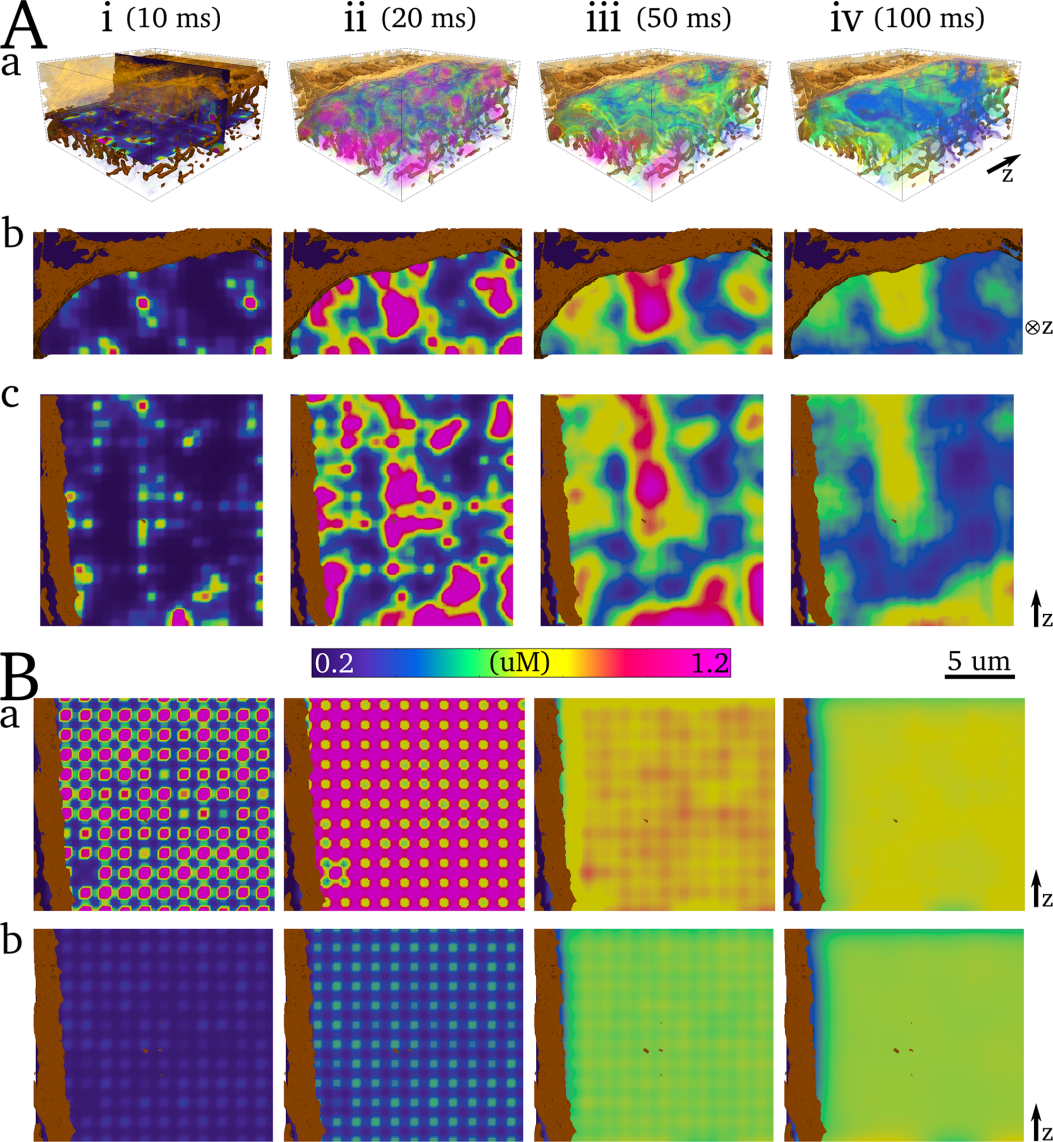
Spatial Ca^2+^ gradients during normal excitation. **Aa)** Intracellular Ca^2+^ concentration in a 3-D volume of the cell. A transparent volume render is used with spikes of increased opacity at various intervals to provide contour lines. Surface sarcolemma and TT reconstructions are shown in brown contour for reference; **b)** and **c)** correspond to the same 2D slices in **Fig 7**. **B)** 2D slices during normal excitation in the semi-idealised cell model, **a)** is in the plane of the dyads and **b)** is in-between dyad planes. In contrast to **Fig 7**, the colour-map is scaled to reveal the local peaks of Ca^2+^ concentration significantly larger than the whole-cell average and highlight the gradients–note also the lower limit. Further renders of the 3-D intracellular Ca^2+^ gradients can be found in in the S3 Fig and a high resolution version is available at http://physicsoftheheart.com/.

Snapshots of spatial Ca^2+^ concentration in 3-D (Fig 7C) and with enhanced scaling (Fig 8A) reveal the 3-D structure of the Ca^2+^ gradients associated with normal excitation. Properties of Ca^2+^ gradients during the different phases of the transient are determined by intracellular structure and the nature of the various fluxes controlling Ca^2+^ dynamics.

The discrete and non-uniformly distributed dyads, heterogeneity in the dyadic cleft volume and small protein numbers (which enhances stochastic state transitions of the RyRs and LTCCs), combined with the rapid transient morphology of *J*_*rel*_ during excitation led to a rapid upstroke of the Ca^2+^ transient and significant spatial heterogeneity during this initial excitation phase (Fig 7Ci; Fig 8Ai). *J*_*rel*_ quickly terminates at around the time of the transient peak (S4 Fig)—the Ca^2+^ influx throughout the cell is significantly reduced and Ca^2+^ diffuses away from the dyads through the bulk cytoplasm, reducing the peaks surrounding the dyads and smoothing the gradients. Due to significant Ca^2+^ buffering, diffusion is slow and gradients are not eradicated (Fig 7Ciii; Fig 8Aii,iii). The decay of the Ca^2+^ transient is slower and more spatially uniform due to the spatially and temporally continuous nature of the effluxes, *J*_*Mem*_ and *J*_*SR*_, (Fig 7Civ; Fig 8Aiv). Subcellular heterogeneity was also observed during controlled AP clamp conditions.

Comparison to the semi-idealised cell model (which contains a uniform dyad distribution—See Methods: Semi-idealised and perturbed structure models) reveals that the complex 3-D structure of Ca^2+^ gradients is largely determined by the dyad distribution (compare Fig 8A with 8B). Temporal variation in the activation time of individual dyads results in significant gradients during the first few miliseconds of excitation in the semi-idealised model, but then much more uniform Ca^2+^ concentration was observed during the main excitation phase, compared to the fully detailed structural cell model (Fig 8). Inclusion of realistic flux distribution in the semi-idealised model enhances spatial heterogeneity but has a much smaller impact than the dyad distribution.

### Spatio-temporal Ca^2+^ dynamics in the network SR

**Fig 9:**
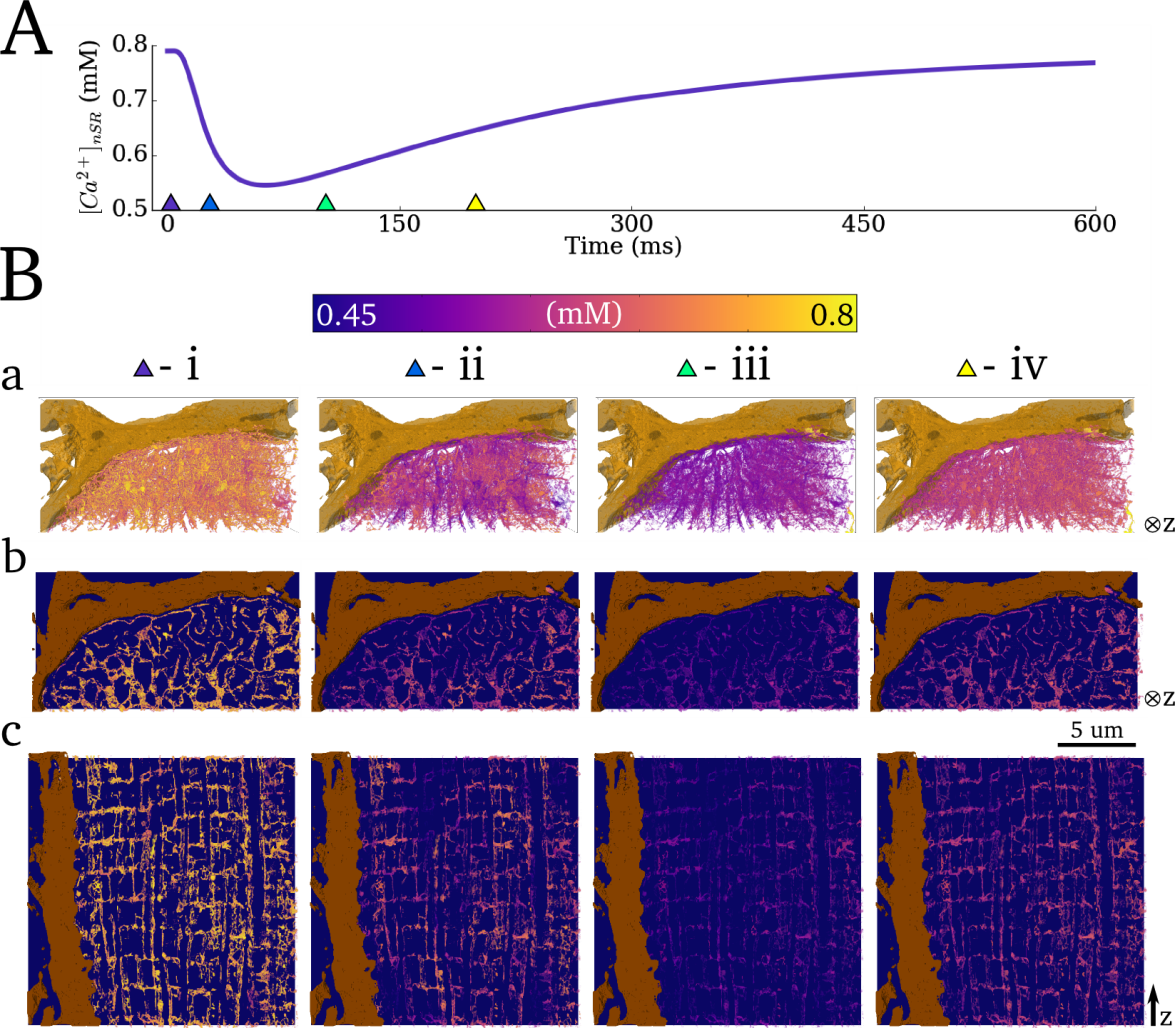
Spatio-temporal Ca^2+^ dynamics in the network SR. **A)** Whole-cell SR Ca^2+^ concentration during a single beat. **B)** Temporal snapshots of spatial Ca^2+^ concentration **a)** in the full 3-D network SR; **b)** and **c)** in pseudo-slices of the cross-sectional and longitudinal planes. Pseudo-slices are comprised of thin 3-D clips and are equivalent to the slices in previous figures. Triangular markers in (**A**) indicate the timings of the corresponding panels (**Bi-iv**)**–** 10ms, 25ms, 100ms and 200ms relative to the applied stimulus. A high resolution version is available at http://physicsoftheheart.com/.

Spatial Ca^2+^ gradients in the nSR were less pronounced than in the cytoplasm (Fig 9; S1 Video). Nevertheless, gradients were observed during the initial excitation (emptying) phase, due to the distributed dyads/jSRs (Fig 9i and 9ii). The spatial distribution is almost uniform at the time of maximum depletion of the SR (Fig 9iii). During the refilling phase (Fig 9iv), gradients were observed but are more uniform than those during the initial phase (compare Fig 9 panel iii with v), comparable to the behaviour observed in the bulk cytoplasm.

### Evaluation of spatial distribution of fluxes in the cytoplasm

**Fig 10:**
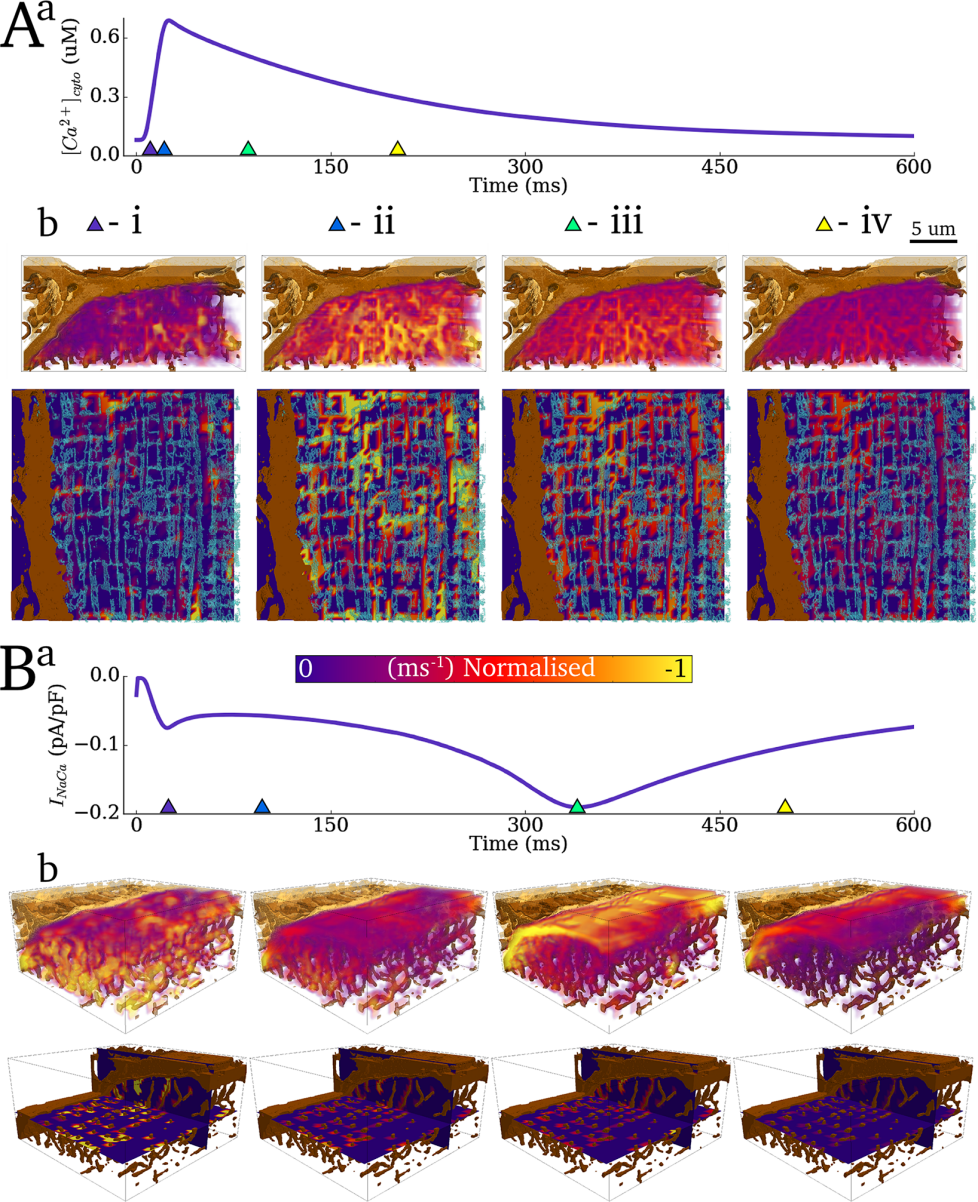
Evaluation of *J*_*up*_ and *J*_*NaCa*_ fluxes in the cytoplasm. **A)** Spatial distribution of the magnitude of *J*_*up*_ (**b**) with the Ca^2+^ transient shown as a reference (**a**). The blue semi-transparent contour render is a pseudo-slice of the SR for reference. **B**) Spatial distribution of the magnitude of *J*_*NaCa*_ (**b**) with the whole-cell average current shown as a reference (**a**). Triangular markers in the upper panels indicate the timings relative to the applied stimulus of the corresponding lower panels **i-iv.** For *J*_*up*_ (**A**) these correspond to 10ms, 25ms, 100ms and 200ms; for *J*_*NaCa*_ (**B**) these correspond to 25ms, 100ms, 345ms and 500ms.

Fluxes acting on the cytoplasm in a single portion of the 3-D cell were analysed (Fig 10). The spatial distribution of *J*_*up*_ (Fig 10A) and *J*_*NaCa*_ (Fig 10B) can be clearly seen, and correspond to the nSR and surface sarcolemma/TT structures, respectively. Spatial gradients in the magnitude of both of these fluxes are observed, as a direct result of the gradients in intracellular Ca^2+^ concentration (note the spatial correspondence between the gradients in Figs 7, 8 and 10).

The effect of voltage inhibition on the activity of *J*_*NaCa*_ combined with the initial large peaks of Ca^2+^ in locations close to the dyads can be clearly seen by spatially distributed peaks in *J*_*NaCa*_ in the initial phase of excitation (Fig 10Bi), uniform and small fluxes during the bulk of the excitation phase, wherein the Ca^2+^ concentration is large (Fig 10Bii), and a more spatially uniform but larger flux during the decay phase of the Ca^2+^ transient, corresponding to the peak of *I*_*NaCa*_ (Fig 10Biii).

### Intracellular Ca^2+^ transient alternans

#### Production of Ca^2+^ transient alternans

**Fig 11:**
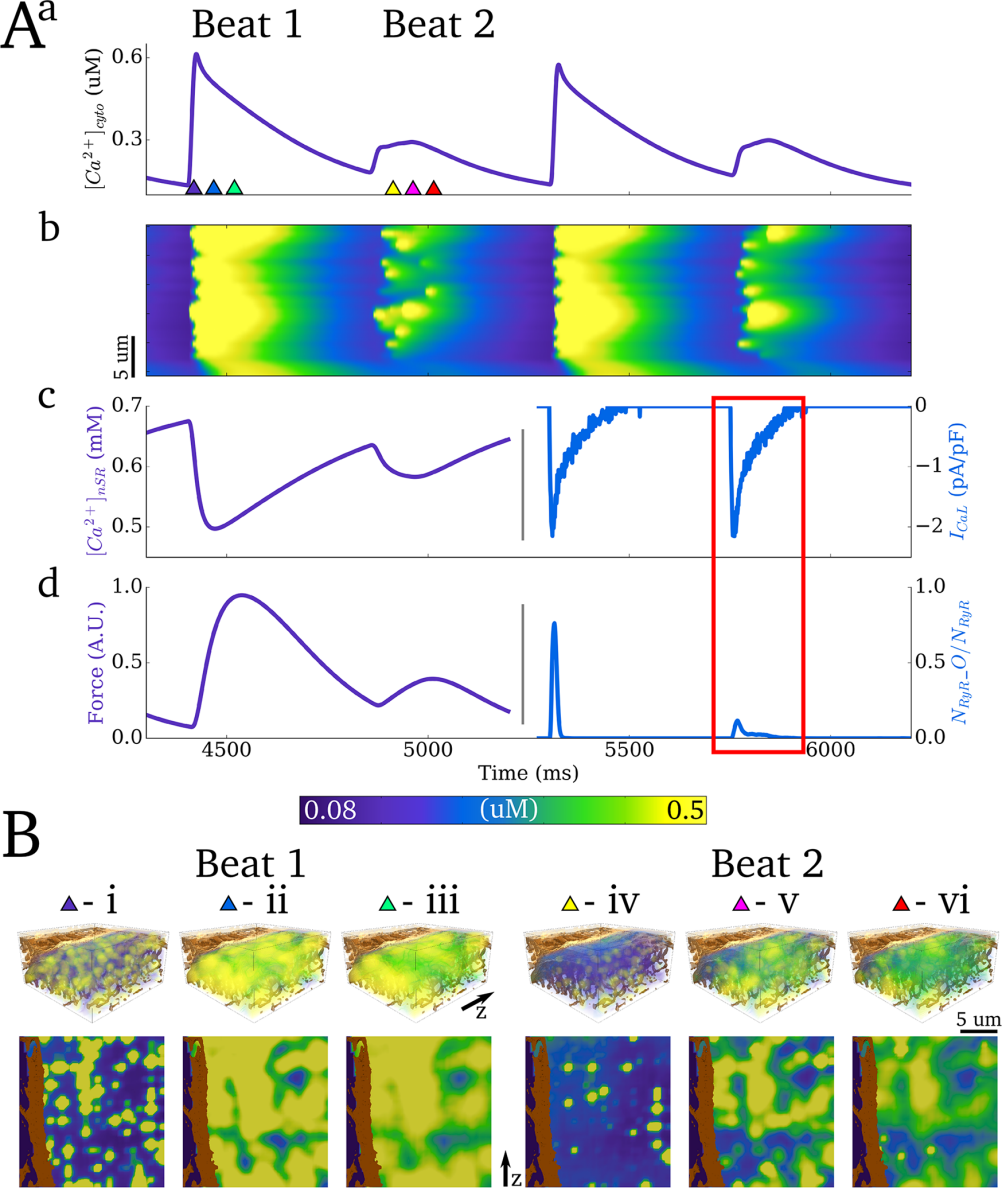
Ca^2+^ transient alternans. **A)** Whole cell characteristics associated with four cycles during Ca^2+^ transient alternans: **a)** Ca^2+^ transient; **b)** linescan along the longitudinal axis of the cross-section portion cell model; **c)** nSR concentration (left y-axis, purple) and *I*_*CaL*_ current trace (right y-axis, blue); **d)** force (left y-axis, purple) and proportion of open RyRs (right y-axis, blue). The role of ICaL and RyR underlying the alternans is highlighted by the red box. **B**) Snapshots of spatial Ca^2+^ distribution in a 3-D portion of the cell (upper panel) and longitudinal slice (lower panel) associated with two beats (large and small amplitude, i-iii and iv-vi, respectively). Timings of snapshots for both beats are identical relative to the applied stimulus: i,iv - 10ms, ii,v - 60ms, and iii,vi - 100ms. Parameters for this figure were: *J*_*up*_ scale = 0.5, LTCC open probability scale = 0.2.0.

**Fig 12:**
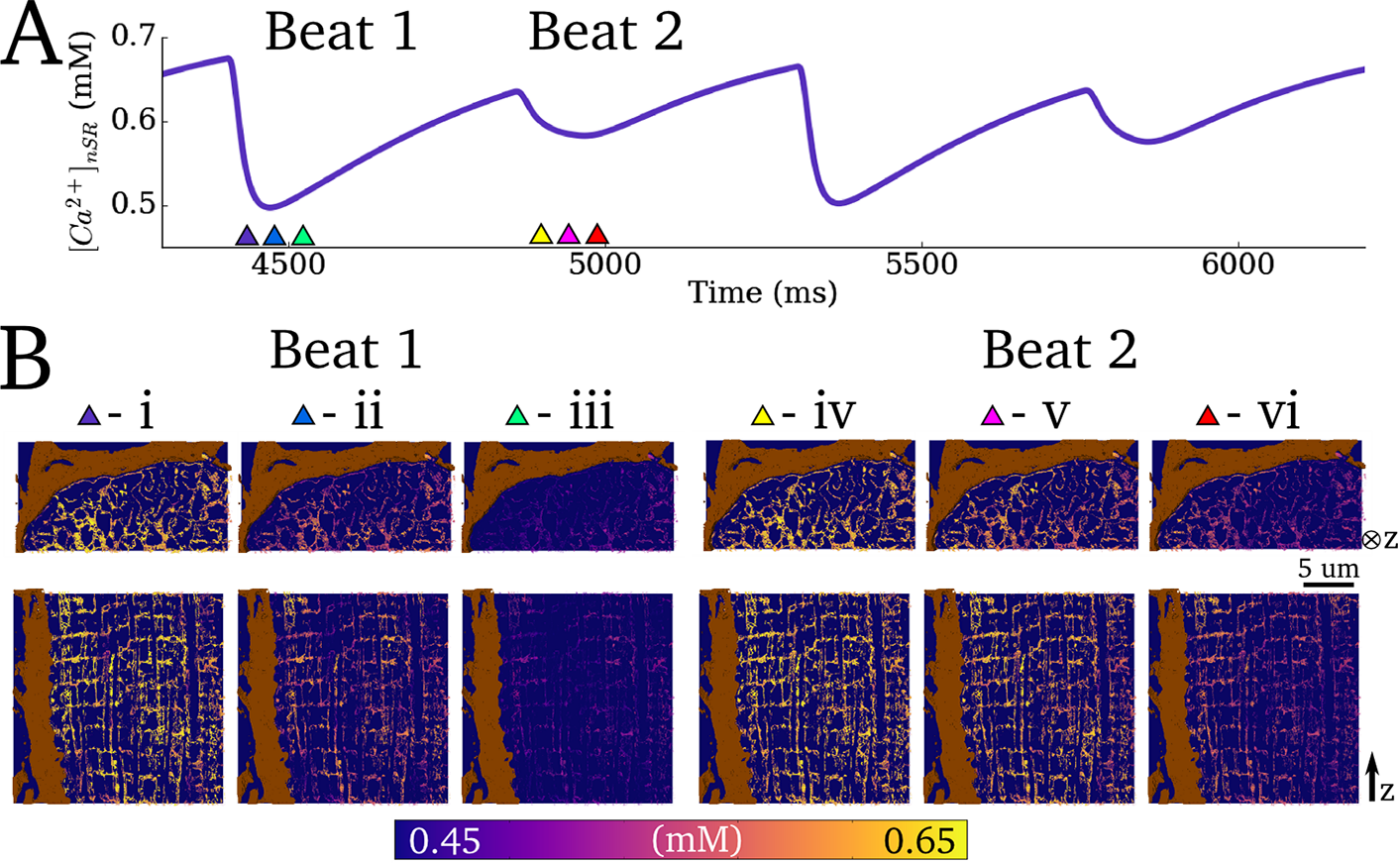
Spatio-temporal SR Ca^2+^ dynamics during Ca^2+^ transient alternans. **A)** Whole cell nSR Ca^2+^ concentration associated with four cycles during Ca^2+^ transient alternans (the same four cycles as in **Fig 11)**. **B)** Snapshots of spatial Ca^2+^ distribution in the nSR associated with two beats (large and small amplitude, i-iii and iv-vi, respectively). Timings of snapshots for both beats are identical relative to the applied stimulus: i,iv - 15ms, ii,v 0–25ms, and iii,vi - 50ms.

To demonstrate the suitability of the model for the study of arrhythmic cellular dynamics, the ability of the model to reproduce Ca^2+^ transient alternans was tested. The maximal flux rate of *J*_*up*_ and the open probability of the LTCCs (transition rate from state d_2_ to d_3_, see S1 Text) were both reduced—factors known to promote alternans. Under these conditions, at a BCL of 450ms, significant Ca^2+^ transient and subsequently contractile force alternans were observed (Fig 11 –cytoplasm Ca^2+^ concentration and force; Fig 12 –nSR Ca^2+^ concentration).

#### Ca^2+^ transient alternans mechanisms and dynamics

Ca^2+^ transient alternans under these conditions was determined primarily by beat-to-beat variation in the magnitude of intracellular release and not variations in the magnitude of I_CaL_ (Fig 11Ac,d); thus, “silent” openings of the LTCCs occur in dyads in which the RyRs are not activated.

The mechanism by which threshold activation of individual dyads manifests as coordinated whole-cell Ca^2+^ transient alternans is illustrated by analysis of spatio-temporal dynamics (Fig 11Ab and 11B; Fig 12B; S2 Video). The magnitude of localised peaks in Ca^2+^ concentration was comparable between the two successive beats, whereas the spatial distribution of the peaks was significantly perturbed associated with the small amplitude cycle. Thus, whereas individual dyads undergo threshold release, the small amplitude whole cell release is a result of only a proportion of the dyads becoming activated by the LTCCs combined with failure to recruit neighbours, whereas significant initial activation and successful recruitment (aided by the larger SR Ca^2+^ concentration) ensure global dyad activation associated with the large amplitude cycle. Note also a significantly increased temporal variation in the activation of dyads which undergo threshold release during the small amplitude cycle, and beat-to-beat variation therein (Fig 11Ab).

**Fig 13:**
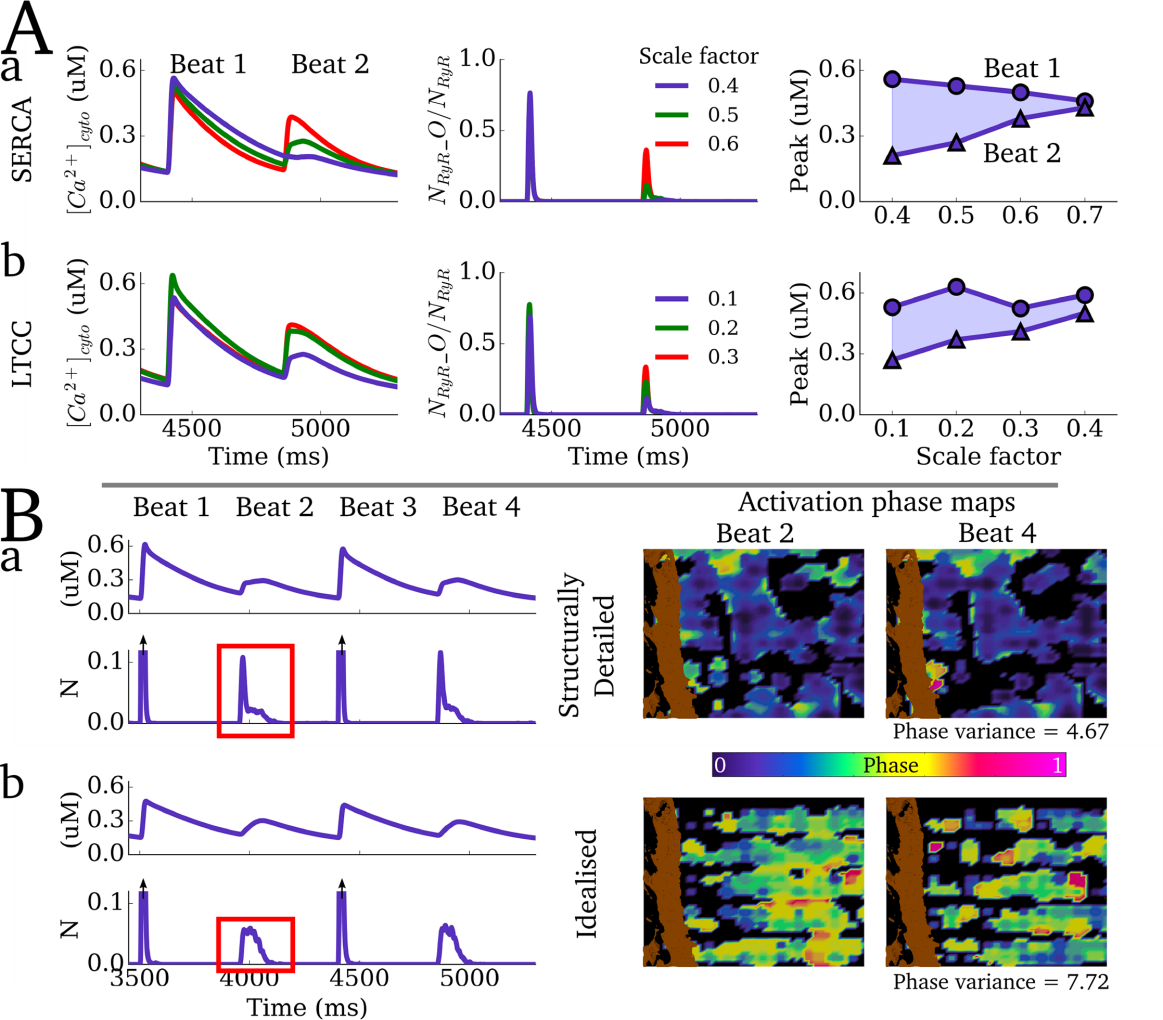
Properties of Ca^2+^ transient alternans. **A)** Alternans magnitude associated with varying the maximal flux rate of SERCA (**a**) and scaling the open probability of the LTCCs (**b**). **B)** Comparison of alternans dynamics between the structurally detailed (**a**) and semi-idealised models (**b**). The right panels show phase maps associated with two successive small amplitude cycles. The phase is calculated as the difference in activation time between a considered voxel and the stimulus, and normalised to the latest measured activation (180 ms after *t*_*stim*_). A threshold of 0.3 μM was used to determine a voxel’s activation, in order to capture the areas surrounding active dyads. The phase variance is given as the summed difference in activation phase maps over several successive small amplitude cycles, normalised to the summed difference of the activation phase maps in the large amplitude cycles.

Further analysis revealed a graded response of the magnitude of Ca^2+^ transient alternans to modifications of the maximal flux rate of *J*_*up*_ and open probability of the LTCCs (Fig 13A). Comparison of alternans dynamics with the semi-idealised model reveals the role of structural heterogeneity in determining the spatial structure of dyad activation and recruitment associated with the small amplitude cycle (Fig 13B): patterns were more spatially disordered in the idealised model than the structurally detailed model, where intracellular structure constrained the probabilistic nature of activation of individual dyads during the small amplitude cycle and reduced beat-to-beat variability in the phase (Fig 13B). This also manifests as a significant difference in the morphology of the RyR waveform on the small amplitude cycle between the two types of model: the idealised model has a smooth dome morphology whereas the structurally detailed model has a spike and decay morphology. Comparison with a model with fewer dyads also highlights the important role of recruitment in the small amplitude cycle: increased inter-dyad distances led to a failure of recruitment, and a significantly smaller proportion of the dyads becoming activated associated with the small amplitude cycle.

### Ca^2+^ spark hierarchy and spontaneous release events

#### Ca^2+^ spark hierarchy

**Fig 14:**
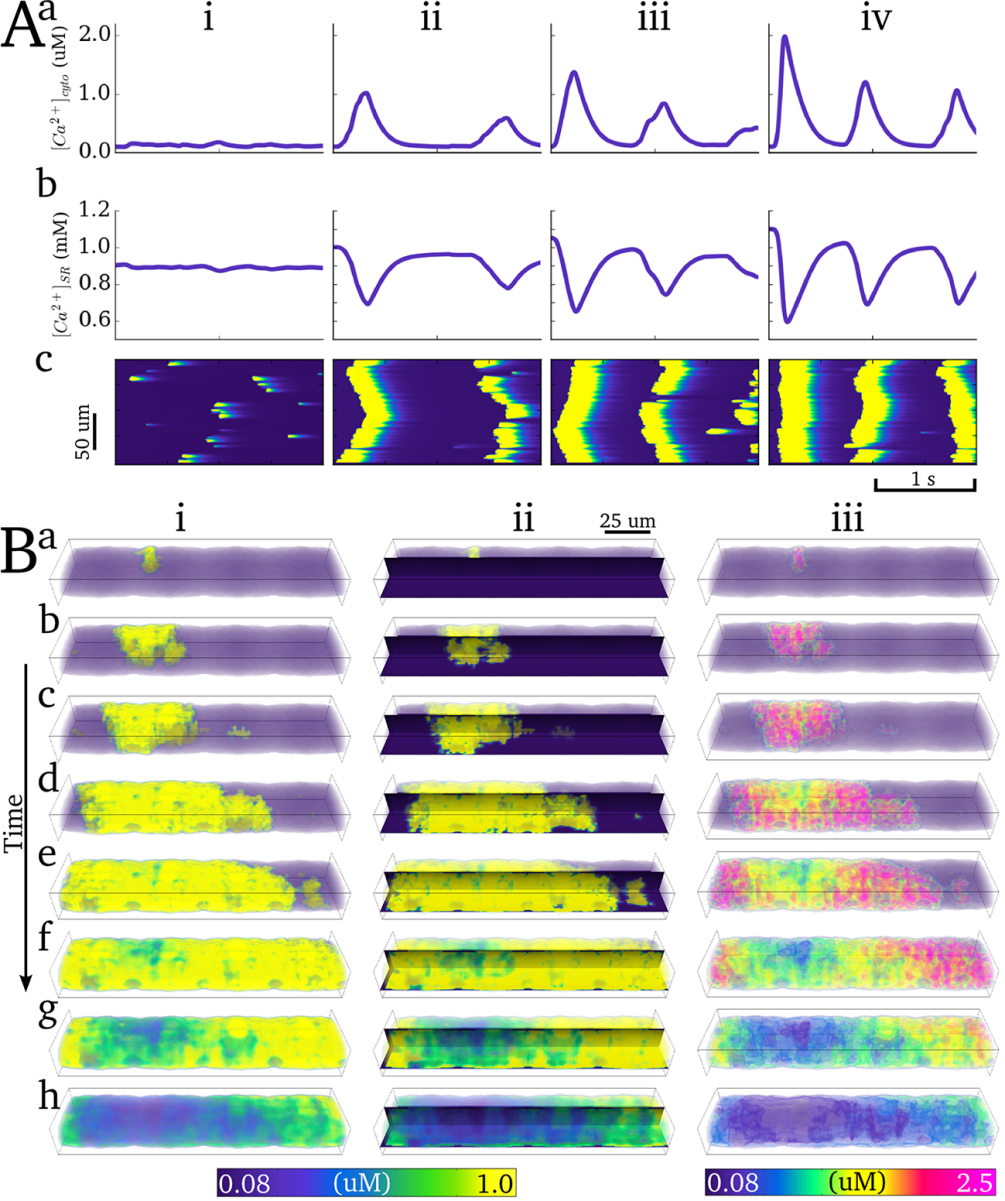
Spontaneous Ca^2+^ release events. **A**) Ca^2+^ spark hierarchy associated with various SR Ca^2+^ loads (i—0.9 mM, ii—1.0 mM, iii—1.05 mM, iv—1.1 mM), illustrating the whole cell average for the cytoplasm (**a**) and nSR (**b**) Ca^2+^ concentration and a longitudinal linescan of the cytoplasm (**c**). **B**) Snapshots (**a-h**) of a coordinated intracellular Ca^2+^ wave in 3-D (i,iii) and in both longitudinal plane slices (ii). i and ii are scaled to the whole cell average; iii uses enhanced scaling with varying transparencies to enhance contours, as in **Fig 8**. The visualisation corresponds to the first spontaneous release event at 1.05 mM nSR Ca^2+^ concentration, shown in **Aiii**. The value of **D** in the subspace (see S1 Text) was 1 μm/ms to produce the single coordinated wave.

Ca^2+^ spark hierarchy was demonstrated through a varied SR Ca^2+^ concentration quiescent protocol: the initial conditions describing SR concentration were varied from 0.8 to 1.2 mM and the model was left in a quiescent state for 2 simulation seconds (Fig 14A). To simulate coordinated whole-cell Ca^2+^ waves, the RyR distribution was also increased to promote Ca^2+^ propagation: additional RyR clusters were introduced along the SR (see next section and Methods: Semi-idealised and perturbed structure models). Spontaneous Ca^2+^ release hierarchy was observed, pertaining to non-propagating Ca^2+^ sparks at SR Ca^2+^ concentrations above normal diastolic values but below threshold (Fig 14Ai), the emergence of whole-cell Ca^2+^ waves above the threshold SR Ca^2+^ concentration (Fig 14Aii and 14B; S3 Video), and multiple and rapid waves significantly above threshold (Fig 14Aiii,iv). The spark-induced-spark mechanism of Ca^2+^ wave propagation is clear at the enhanced visualisation scale (Fig 14Biii).

#### Criticality in Ca^2+^ spark propagation

**Fig 15:**
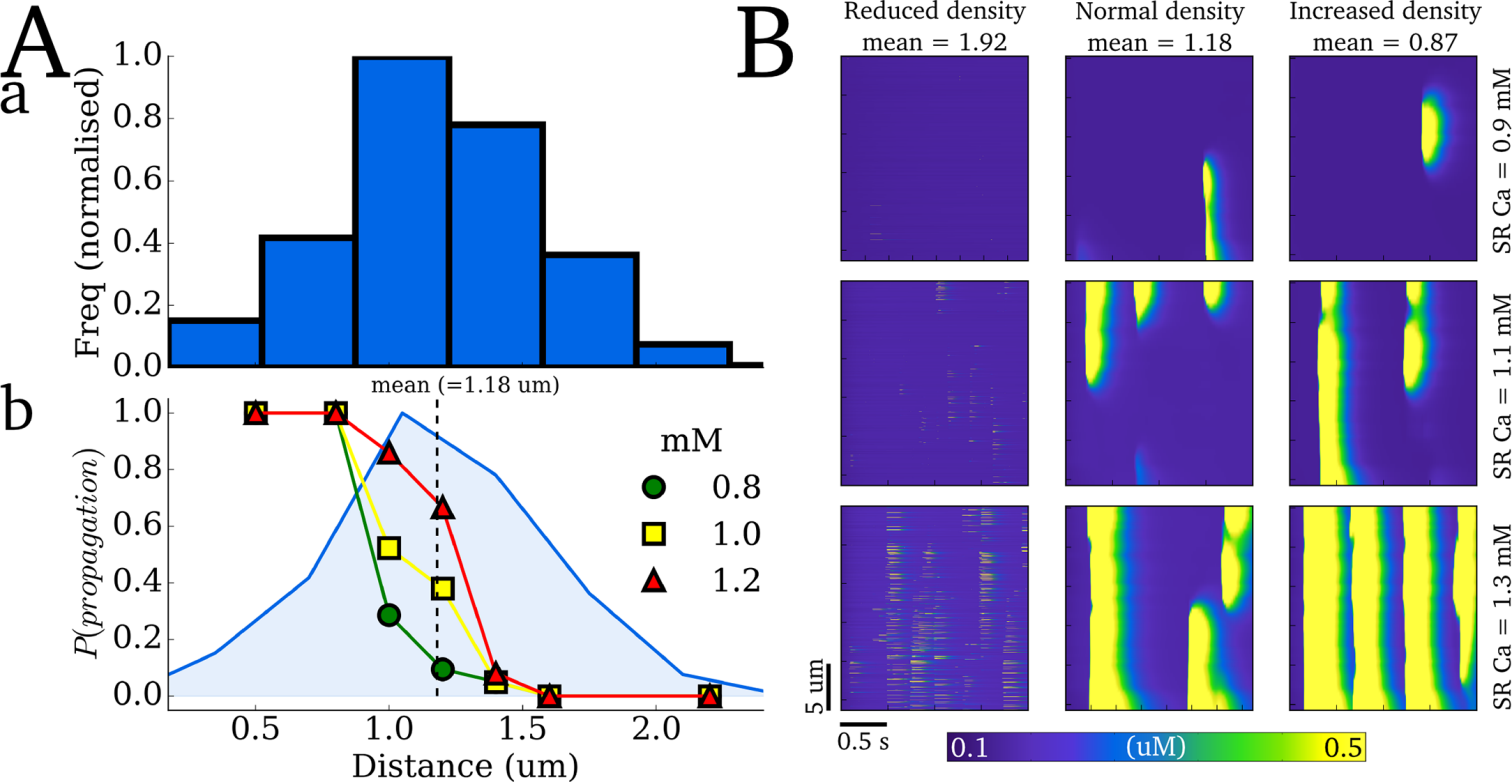
Criticality in Ca^2+^ spark propagation. **Aa)** Histogram of the nearest neighbours for each dyad in the structurally detailed model and **b)** probability of Ca^2+^ spark-induced-spark propagation in a two-dyad model at different SR Ca^2+^ concentrations (indicated in the figure legend). The dyad distribution is imposed for reference and the mean nearest neighbour inter-dyad distance is show by the dotted line. **B)** Ca^2+^ spark hierarchy at different mean nearest neighbour distances (left to right) for different SR Ca^2+^ loads (top to bottom), simulated in the cross-sectional portion cell model.

A two-dyad model was constructed to study the effect of inter-dyad distance on the probability of successful propagation of Ca^2+^ sparks. Ca^2+^ sparks were triggered in the first dyad by opening of the LTCCs during AP clamp conditions and the probability of the second dyad becoming activated was determined over a range of distances and SR Ca^2+^ concentrations (0.8, 1.0 and 1.2 mM). A rapid decay of the probability of successful propagation with distance was observed for all SR Ca^2+^ concentrations (with larger SR Ca^2+^ increasing the gradient of the probability curve relative to distance, Fig 15A). Note that these maximum distances for successful propagation lie within the distribution of nearest-neighbour inter-dyad distances (Fig 15A), indicating that this heterogeneity in inter-dyad distances is critical to the maintenance of coordinated whole-cell intracellular Ca^2+^ waves.

This is further demonstrated through dynamics of Ca^2+^ spark hierarchy with different dyad distribution densities (Fig 15B): with significantly increased inter-dyad distances, Ca^2+^ sparks fail to propagate. Even at very large [Ca^2+^]_SR_, Ca^2+^ sparks occur in many dyads but fail to coordinate into propagating waves (Fig 15B). Conversely, with decreased inter-dyad distance (increased density), Ca^2+^ sparks more easily propagate leading to whole cell waves at lower [Ca^2+^]_SR_ than the normal distribution (Fig 15B). With both the normal and increased density distributions, the main properties of Ca^2+^ spark hierarchy were reproduced.

#### Spontaneous release events during rapid pacing

**Fig 16:**
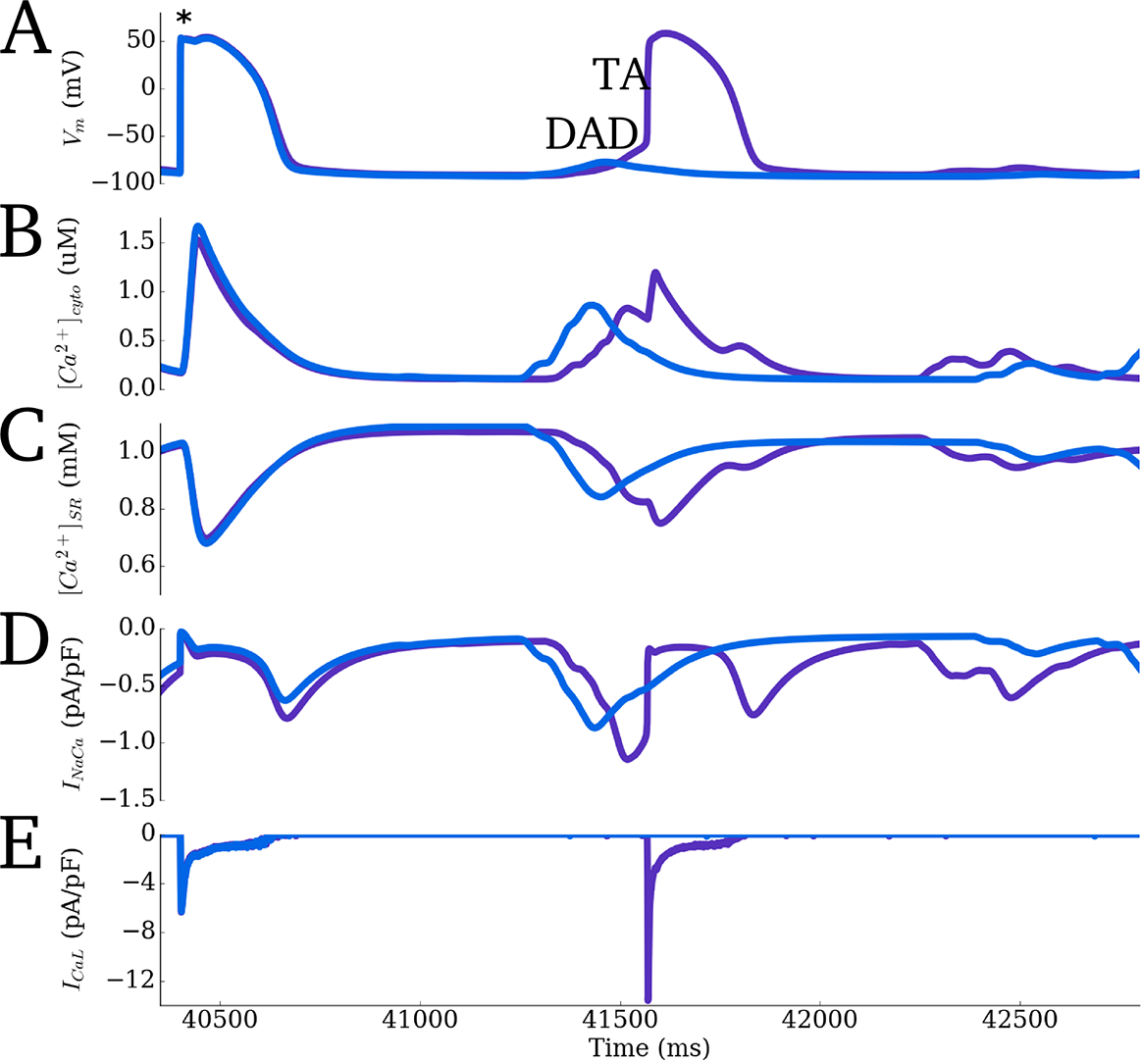
Rapid pacing induced spontaneous release. Final paced excitation and quiescent period of a train of rapid pacing (BCL = 400 ms) with enhanced *I*_*CaL*_, *I*_*NaCa*_ and *J*_*up*_. **A**) Membrane potential (V_m_). **B,C)** Intracellular and SR Ca^2+^ concentration traces. **D,E)** *I*_*NaCa*_ and *I*_*CaL*_ current traces. Purple trace corresponds to a larger increase of *I*_*NaCa*_ than the blue. The asterisk in A indicates the timing of the final applied stimulus. Delayed-after-depolarisations (DADs) are observed in both cases caused by spontaneous Ca^2+^ release; with the largest *I*_*NaCa*_, the threshold of activation for a triggered action potential (TA) is surpassed.

Spontaneous activity during excitation was investigated using an SR Ca^2+^ loading protocol: the maximal uptake rate of *J*_*up*_ and open probability of the LTCCs were increased by factors of between 1.5 and 2.5 and the model paced to steady state (100 beats) at a rapid cycle length of 400ms, before being left for a quiescent period. Under significant SR loading, spontaneous release events were observed which underlie delayed-after-depolarisations (DADs) (Fig 16). Combined with enhanced *I*_*NaCa*_ activity (2-fold increase in maximal flux rate), DADs manifested as triggered APs (Fig 16).

### SR diffusion properties

**Fig 17:**
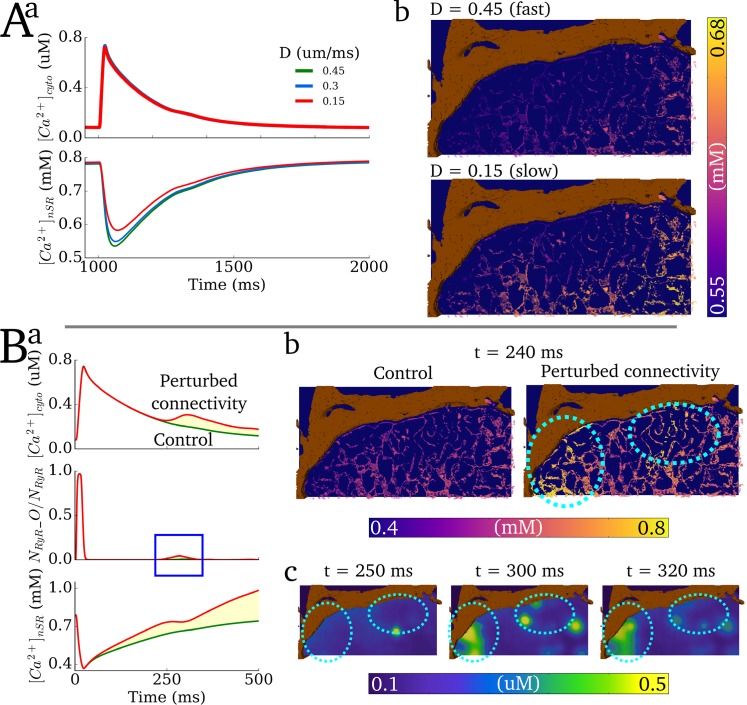
Varying SR diffusion properties. **A)** Effect of varying SR diffusion coefficient (D) on whole-cell characteristics (**a**) and SR Ca^2+^ gradients (**b**) during normal excitation. The snaphots in **b)** are taken 50 ms after the stimulus time. Note the scaling of the colour map to reveal the differences between the two conditions. **B)** The effect of perturbing the SR connectivity on normal excitation. **a)** Whole-cell characteristics, demonstrating secondary systolic Ca^2+^ release; **b)** Snapshots of SR Ca^2+^ concentration taken at 240 ms after the stimulus, comparing control to the perturbed connectivity. Islands of SR loading are highlighted by the dotted-blue ovals; **c)** snapshots of intracellular Ca^2+^ concentration during secondary systolic Ca^2+^ release. The same locations as in (**b**) are highlighted.

Varying the SR diffusion parameters primarily affected the spatial-distribution of Ca^2+^ in the SR: Rapid diffusion in the SR facilitated equilibration of the SR Ca^2+^ content and reduced gradients; slower diffusion enhanced Ca^2+^ gradients (Fig 17A). However, the impact on whole-cell dynamics was relatively minimal under the variance in diffusion coefficient of 0.5–2 times the baseline value (0.3 μm/ms) considered in this study.

Reduced connectivity in the SR network led to significant SR Ca^2+^ gradients during normal excitation, with islands of SR loading appearing during the systolic phase (Fig 17B). This localised early SR loading promoted secondary spontaneous release during the AP–note the locations of secondary release correspond to the islands of SR loading.

## Discussion

In this study, a model of 3-D spatio-temporal Ca^2+^ dynamics in a large mammal ventricular myocyte was developed which incorporates significant details of intracellular Ca^2+^ handling structures. This is the first to employ 3-D reconstructions of the TT network and SR for cardiac myocytes in situ within tissue blocks, derived from SBF-SEM (Fig 1). We describe how the structures were processed to form computational grids and mapping functions and incorporated into a 3-D spatio-temporal Ca^2+^ handling mathematical model, accounting for realistic cytoplasm and nSR structure and fluxes distributed according to the imaging data (Figs 2–5). Our simulations illustrate that the model accurately reproduces excitation characteristics in normal conditions (Figs 6–9) as well as perturbed conditions leading to Ca^2+^ transient alternans and spontaneous release events (Figs 11–14), and reveals high resolution 3-D Ca^2+^ gradients and fluxes associated with excitation (Figs 8–10). Furthermore, we provide preliminary analysis which demonstrates the importance of intracellular structures in determining the spatial distribution of dyad recruitment during Ca^2+^ transient alternans (Fig 13) and Ca^2+^ wave propagation (Fig 15) as well as the role of SR connectivity in maintaining stable Ca^2+^ dynamics (Fig 17).

### Comparison to previously developed models

There are numerous exemplary studies in recent years implementing spatio-temporal Ca^2+^ handling models in multiple dimensions (e.g. [17–41]). These models have been used to mechanistically evaluate physiological and pathophysiological dynamics in the intracellular Ca^2+^ handling system, such as graded release [20], RyR dynamics [23,40], Ca^2+^ transient alternans [20,21,25,27,30], Ca^2+^ waves [19,20,41] and pacemaker activity [38], and account for varying degrees of detail of intracellular structure (realistic RyR distribution; reconstruction of single TT; super-resolution of single dyad). However, no model has yet accounted for the realistic structure of the nSR, nor integrated fluxes according to realistic membrane structure, dyad distribution and nSR structure at the whole cell scale.

The model developed in the present study achieves this goal, providing for the first time a framework to directly integrate imaging data on multiple structures with whole-cell modelling. A major feature of the model is the ability to account for the structure of the network SR at the whole-cell scale. Identifying the network-like structure of the model as the key feature to be captured provides a method to model Ca^2+^ diffusion throughout the nSR at lower resolutions than required to image this structure, through the reduction to a 3-D network of 1-D strands (Fig 3).

Whereas idealised geometry based models offer ease of data interpretation and are suitable for general analysis of Ca^2+^ dynamics, the modelling approaches presented in this study provide a method to remove much of the uncertainty inherent to models based on idealised geometries and to directly asses how structure-function relationships are affected by variability in intracellular structure, which may be particularly relevant when considering structural remodelling associated with disease [7]. The present approach provides the first framework which allows multiple structural datasets to be directly integrated with mathematical modelling of Ca^2+^ dynamics without the assumptions required by idealised models to capture structural variability, which accentuate the inherent uncertainty of these models. This, therefore, potentially significantly increases the confidence of simulation data regarding variability in intracellular structure.

On the other hand, models of specific regions of the cell which incorporate realistic structure can offer significant understanding of local control of EC coupling, but cannot capture whole-cell emergent properties such as the interaction of heterogeneity of these structures throughout the cell. The model presented here therefore complements those previously developed, providing a framework to investigate whole-cell dynamics underlain by real structure and heterogeneity.

We note that, in support of the validation of the model, results in the present study are consistent with those of previous modelling studies where comparable. For example, Izu et al. 2006 [17] found similar results regarding the criticality of inter-dyad distances in maintaining the propagation of Ca^2+^ waves. Ca^2+^ spark hierarchy is similar to that shown in Nivala et al. 2012 [19]. The mechanism underlying Ca^2+^ transient alternans is consistent with the studies of Restrepo et al. 2008 [20], Rovetti et al. 2010 [21] and Alvarez-Lacalle et al. 2015 [25].

### Reproducing dynamics under different conditions

The suitability of the model for future research was demonstrated by its application under multiple conditions. The model reproduces whole-cell and spatio-temporal Ca^2+^ characteristics under control pacing (Figs 7–9), rapid perturbed pacing leading to alternans (Figs 11–13), and rapid pacing leading to SR overload, spontaneous Ca^2+^ release and the development of single-cell triggered activity (Figs 14–16).

### The role of structure underlying calcium dynamics

The primary focus of the study was to develop an approach to overcome the challenges inherent in the construction of such a detailed model and to demonstrate the suitability of the model for future research, which was achieved through application of the model in normal and arrhythmic excitation conditions. The intention of the model is for future studies to incorporate multiple datasets, including those describing multiple disease conditions, to fully assess the role of variability in intracellular structure in determining potentially pro-arrhythmic dynamics; such detailed analysis was therefore beyond the scope of this study.

This notwithstanding, the present study also provides for the first time detailed and high resolution (spatial, temporal and concentration) reconstruction of Ca^2+^ gradients and fluxes in 3-D in both the cytoplasm and network SR (see Results sections: Spatio-temporal Ca^2+^ dynamics in the bulk cytoplasm; Spatio-temporal Ca^2+^ dynamics in the network SR; Evaluation of spatial distribution of fluxes in the cytoplasm), as well as preliminary analysis of the importance of structure underlying spatio-temporal Ca^2+^ dynamics and the role of SR diffusion and connectivity (see Results sections: Intracellular Ca^2+^ transient alternans; Ca^2+^ spark hierarchy and spontaneous release events; SR Diffusion Properties).

Comparison with the semi-idealised model revealed similarities and differences between the two levels of detail included in the model. The similarities of whole-cell characteristics and gross spatio-temporal dynamics between the two models during normal structure highlights the suitability of idealised models for general and mechanistic modelling of spatio-temporal dynamics associated with normal structure.

However, multiple results presented here also emphasise the important role of structure and heterogeneity underlying the complex and fine-scale details of 3-D intracellular Ca^2+^ dynamics, which may be particularly important for disease models of intracellular structural remodelling.

First, the complex structure of 3-D Ca^2+^ gradients and intracellular Ca^2+^ fluxes arises primarily as result of dyad distribution and intracellular structure; this complex structure does not emerge in the idealised cell model. Moreover, spatially heterogeneous distributions of the membrane and SR Ca^2+^ fluxes were observed despite homogeneous distribution of the maximal flux rates, as a direct result of these intracellular Ca^2+^ gradients.

Second, the degree of disorder associated with alternans dynamics was significantly reduced in the structurally detailed model compared to the idealised model; dyad distribution significantly affects recruitment patterns and therefore spatially constrains the probabilistic nature of recruitment, with real structure reducing the phase variance associated with multiple small-amplitude cycles. Similarly, reduced density of dyad distribution led to significant failure to recruit and very small transients associated with the small amplitude cycle. These results thus add further insight to those gained in previous studies [20,25,27].

Third, the critical inter-dyad distances to maintain Ca^2+^ propagation are within the distribution of dyad nearest neighbour distances; whereas the overall trend of Ca^2+^ spark hierarchy is preserved under different dyad distributions, the propagation patterns emerging and dependence on SR Ca^2+^ concentration were significantly affected by dyad distribution, with whole-cell coordinated single waves requiring relatively short inter-dyad distances.

Finally, the connectivity of the network SR was vital in maintaining normal Ca^2+^ dynamics; reduced connectivity led to failure of the SR to equilibrate, significant and heterogeneous SR loading and secondary systolic Ca^2+^ release.

These results highlight the necessity for integrated multi-scale models which can capture realistic structure of both the intracellular and SR spaces as well as heterogeneity in dyad properties and flux distribution. For example, a combination of increased heterogeneity in dyad properties with remodelling of dyad distribution may result in highly unpredictable constraints on dynamics. TT structure, dyad distribution and SR structure have all been shown to be perturbed in both animal models of disease and patients [7–14]. Whereas many previous modelling studies have investigated remodelling in proteins and flux dynamics in the single cell (e.g., [16]), intracellular structural remodelling has only been investigated by a few computational studies and these have been idealised (e.g., [52]). Application and analysis of structurally accurate models such as the one presented in this study will therefore allow mechanistic evaluation of the role of structural remodelling in determining arrhythmogenic electrical and perturbed contractile dynamics at the cellular scale as well as structure-function relationships underlying normal cardiac excitation.

### Generalisation of the model

Whereas the present study includes a novel mathematical model describing intracellular Ca^2+^ dynamics, the primary focus was on the methods to process and integrate structural datasets into modelling frameworks. To demonstrate the generalisability of these approaches, the structural model was integrated with the independent mathematical model of Nivala et al. 2012 [27]. Note that this model contains a different schematic structure (with no subspace) and exhibits a larger Ca^2+^ transient associated with excitation.

The whole-cell characteristics of the integrated model were comparable to that of the Nivala et al. study, whereas the structure of intracellular Ca^2+^ gradients is comparable to those previously shown in the present study; Ca^2+^ diffusion is aided in the Nivala et al. version of the model as a result of the larger transient, and thus the quantitative comparison of the gradients reveals some differences–nevertheless, the main structure remains (S5 Fig). This therefore demonstrates the generalisability of the approaches for integration with independent mathematical models and further supports the suitability for future research.

### Limitations of and future extensions to the model

For the purpose of the methodological development of the model, only a portion of the myocyte was reconstructed at high resolution and processed to form the computational grids and mapping functions used for simulation. For specific studies of variability in Ca^2+^ dynamics, especially those in disease where intracellular structure may be highly irregular, a larger or full-cell reconstruction would be necessary. However, it should be noted that this does not affect the fundamental model itself nor the methodological approaches for structural data processing.

In the model, the membrane and SR fluxes were spatially distributed according to maps created from the reconstructions. Within this structure, the fluxes were distributed evenly and continuously. Incorporation of immuno-labelling data describing the realistic distribution of the proteins responsible for these fluxes would provide a further degree of accuracy in the cell model as well as a tool for investigation of the functional impact of changes to the distribution of these flux-carrying proteins; arbitrary heterogeneity could have been introduced into the present model but this was avoided due to the inherent uncertainty in such an approach–the purpose of this model being to remove this necessity for future research. In further simulations, we also demonstrate that redistribution of *I*_*NaCa*_ primarily to the TTs, as has been performed in other studies [24], results in preferential flux in the TTs without significant perturbation to whole-cell dynamics (S6 Fig).

The model includes a restricted buffering subspace which functionally couples neighbouring dyads. The inclusion of this subspace was primarily motivated by considerations for reproducing physiological Ca^2+^ dynamics. Whereas the presence of pathways between the localised Ca^2+^ buffers may be physiologically relevant, it is not the intention of this study to make a comment and such functionality requires further investigation experimentally. We, however, note that similar constructs are present in previously developed models from other groups: in both Gaur-Rudy 2011 [28] and Voigt et al. 2014 [26], neighbouring dyadic spaces are directly coupled. The present model contains this functional coupling but also preserves the restricted local spaces of individual dyads. This construct is not critical to the primary novelty of the developed approach–in processing and modelling with the structural datasets–as demonstrated by integration with the independent mathematical model of Nivala et al., which does not contain this subspace (see Discussion: Generalisation of the model).

The dyads/jSRs were treated as point sources (single voxels) in the spatial geometries. Whereas heterogeneity was incorporated through functionality to vary dyadic cleft volume and protein numbers (NRyR and NLTCC), the spatial structure of the dyad, including local RyR distribution, is not accounted for in the present model. A multi-scale integration method could feasibly be implemented to integrate previously developed super-resolution models of single dyads [37,53], though this was beyond the scope of the present study. Similarly, the RyR dynamic model is primarily functional rather than rigorously based on experimental data describing RyR kinetics. Incorporation of a more accurate RyR model, such as the recent induction decay models [40] would also improve the accuracy and suitability for future studies. Furthermore, heterogeneity in the jSR volume and calsequestrin concentration could be included to further analyse the role of these heterogeneities underlying spatial Ca^2+^ dynamics.

Further possible extensions to the model include segmentation of the mitochondria [39] (the spatial distribution of the mitochondria will affect spatial Ca^2+^ diffusion because they effectively act as barrier to diffusion) and subsequent incorporation of localised energetics. Furthermore, the contractile system could also be segmented for simulation of spatially localised troponin buffering and force generation, for application to understand reduced contractile force associated with heart failure, for example.

### Conclusions

A computational model of 3-D spatio-temporal Ca^2+^ dynamics has been created which incorporates realistic reconstructions of multiple intracellular structures, namely the network SR structure, cytoplasm volume, and fluxes distributed according to the membrane/TT and SR structures. Understanding the role of intracellular Ca^2+^ cycling in physiological and pathophysiological cellular dynamics is vital for mechanistic evaluation of perturbed contraction and arrhythmia associated with Ca^2+^ handling malfunction, and may contribute to improved treatment strategies for prevention, management and termination of life-threatening conditions. The methodological framework and model reported here provide a powerful tool for future investigation of structure-function relationships at the whole cell scale underlying physiological and pathophysiological intracellular Ca^2+^ handling, beyond the insight gained in this study. Full model code is provided (S1 Code) to facilitate realisation of the potential of future applications of these approaches.

## Supporting information

S1 Code Code and documentation. Code provided in C/C++ and includes the structural datasets used in this study. Documentation is provided on use of and updating the code. Updates may also be available on Michael Colman’s website, http://www.physicsoftheheart.com and github repository: https://github.com/michaelcolman/CODE---PLOS-Comp-Biol-2017-Model-SR.
(ZIP)

S1 Text Model description. Contains all equations, variables and parameters describing the developed model.
(PDF)

S1 Fig Comparison between full-sized and cross-sectional portion models. (PDF)

S2 Fig Rate dependence of the Ca^2+^ transient. (PDF)

S3 Fig Further renders of Ca^2+^ gradients in the cytoplasm. (PDF)

S4 Fig *J*_*rel*_ and single-dyad dynamics. (PDF)

S5 Fig Spatio-temporal dynamics in a model without a subspace. (PDF)

S6 Fig Redistribution of *I*_*NaCa*_ to the TTs. (PDF)

S1 Video Spatio-temporal dynamics during a single beat of cardiac excitation. Left panels: Whole cell membrane potential, cytoplasm and SR Ca^2+^ concentration during a single beat. Centre panels: cytoplasm Ca^2+^ concentration in a small portion of the cell in 3-D (upper) and a longitudinal slice (lower) scaling. Right panels: nSR Ca^2+^ concentration in 3D (upper) and longitudinal pseudo slice (lower). Colours are according to the colour bars in Figs 7 and 9.
(AVI)

S2 Video Spatio-temporal dynamics during Ca^2+^ transient alternans. Left panels: Whole cell cytoplasm and SR Ca^2+^ concentration during multiple beats undergoing Ca^2+^ transient alternans. Upper panels: cytoplasm Ca^2+^ concentration in a small portion of the cell. Lower panels: nSR Ca^2+^ concentration. Colours are according to the colour bar in Figs 11 and 12.
(AVI)

S3 Video Spontaneous Ca^2+^ waves in the whole cell. Upper panel shows whole cell proportion of open RyRs and intracellular Ca^2+^ concentration. Middle and lower panels show the 3D Ca^2+^ concentration in the whole cell at normal and enhanced scaling. Video corresponds to the condition illustrated in Fig 14B.
(AVI)
