# Supplementary material for: A computational model of spatio-temporal cardiac intracellular calcium handling with realistic structure and spatial flux distribution from sarcoplasmic reticulum and t-tubule reconstructions
Source: PLoS Comput Biol. 2017 Aug 31;13(8):e1005714. doi: 10.1371/journal.pcbi.1005714 (PMC5597258; doi:10.1371/journal.pcbi.1005714)
Supplement: S1 Code — Code provided in C/C++ and includes the structural datasets used in this study. Documentation is provided on use of and updating the code. Updates may also be available on Michael Colman’s website, http://www.physicsoftheheart.com and github repository: https://github.com/michaelcolman/CODE---PLOS-Comp-Biol-2017-Model-SR. (ZIP) [file pcbi.1005714.s001.zip › PLOS_SR_Clean/Documentation_for_model_code.pdf]

Documentation for code:

A Computational Model of Spatio-Temporal Cardiac Intracellular Calcium Handling with Realistic Structure and Spatial Flux Distribution from Sarcoplasmic Reticulum and T-tubule Reconstructions

Michael A. Colman, m.a.colman@leeds.ac.uk

Accepted by PLOS Computational Biology, August 2017

Disclaimer:

COPYRIGHT MICHAEL A. COLMAN 2017. THIS SOFTWARE IS PROVIDED OPEN SOURCE AND MAY BE FREELY USED, DISTRIBUTED AND UPDATED, PROVIDED: (i) THE BELOW CITATIONS ARE MADE AS APPOPRIATE; (ii) THIS TEXT IS RETAINED WITHIN THE CODE OR ASSOCIATED WITH IT. ANY INTENDED COMMERCIAL USE OF THIS SOFTWARE MUST BE BY EXPRESS PERMISSION OF MICHAEL A. COLMAN ONLY. IN NO EVENT ARE THE COPYRIGHT HOLDERS LIABLE FOR ANY DIRECT, INDIRECT, INCIDENTAL, SPECIAL, EXEMPLARY OR CONSEQUENTIAL DAMAGES ASSOCIATED WITH USE OF THIS SOFTWARE.

ANY use of this code MUST cite this associated paper: "A Computational Model of Spatio-Temporal Cardiac Intracellular Calcium Handling with Realistic Structure and Spatial Flux Distribution from Sarcoplasmic Reticulum and T-tubule Reconstructions" PLOS Comp Biol.

Use of the structural datasets provided MUST also cite *Pinali C et al Circ Res. 2013 Nov 8;113(11):1219–30.*

Use of the implementation of the ORd ionic cell model MUST cite the original paper: *O'Hara T. et al PLoS Comput Biol. 2011 May;7(5):e1002061.* **PLEASE ALSO SEE TEXT AT THE TOP OF lib/memrbane_ord_currents.cpp FOR THEIR OWN STATEMENT REGARDING USE OF THEIR MODEL.**

The Ca handling model uses equations from *Shiferaw Y. et al Biophys J. 2003 Dec;85(6):3666–86* **and** *Shannon TR et al Biophys J. 2004 Nov;87(5):3351–71.*, **as well as using approaches from** *Restrepo JG et al Biophys J. 2008 Oct;95(8):3767–89* **and** *Nivala M et al Front Physiol. 2012;3:114*: **It would also be polite to cite those papers.**

Pseudo-random numbers are generated by *Matsumoto M, Nishimura T. Mersenne twister: a 623-dimensionally equidistributed uniform pseudo-random number generator. ACM Trans Model Comput Simul. 1998 Jan 1;8(1):3–30* **implemented by Richard J. Wagner 2009. PLEASE SEE DISCLAIMER AT THE TOP of "lib/MersenneTwister.h".**

Contents

Notes ... 3
Basic use ... 4
Full list of arguments ... 5
Output files ... 6
Spatial data reconstruction and visualisation ... 8
Adding your own structural datasets ... 9
Adding new dynamics models ... 9
Simulation settings examples ... 10

Notes

This has been provided for general use: please feel free to update the dynamics and incorporate your own datasets. Please still cite this paper, even if all dynamics and structures have been changed. The previous disclaimer applies to all modifications of this code. If you are unsure, please contact me directly.

This documentation has instructions on how to use and run the code, as well as how to incorporate your own structural datasets for future investigation.

Furthermore, settings to reproduce results in the paper are also described.

While tidying the code for this distribution, a couple of small things were noticed which have led to small changes to a few parameters. This does not affect any overall conclusions or the model itself, but please be aware of this if something isn't exactly the same as in the paper.

Please regularly check my website (http://physicsoftheheart.com/) and github repository (https://github.com/michaelcolman/CODE---PLOS-Comp-Biol-2017-Model-SR) for updates and fixes.

It is always possible with code this size for there to be errors and bugs. If you find any, please inform me so I can solve the issues.

**** There may be issues with old versions of compilers due to optimisation. I had an issue with RedHat which did not show up in any other distribution. Please check that your simulations reproduce the control data before progressing ** If they are different, try using "***Single_cell_structural_3D_with_SS_alternative.cc*" **as your Main – this is slightly less optimised, but solved the issue I had with RedHat. *** If the problem persists, please contact me.**

Basic use

This code is designed to be used within a linux command line environment.

1. First, you must select the appropriate main file.

There are two provided:

Single_cell_structural_3D_with_SS.cc || Main with SS at multiple discretisation

Single_cell_structural_3D_no_SS.cc || Main with no SS

Note: The primary difference between the models (other than presence of subspace) is cytoplasm volume and the magnitude of the transient. The subspace model was created to maintain a realistic transient (~0.7 µM) and allow propagation; other published models which have Ca^{2+} propagation and no subspace typically have a larger transient (~2µM or more – see Nivala et al for eg) and this is what is observed in the no SS model. The SS model thus has a nicer magnitude transient, but is more artificial in its coupling; the non-SS model is more natural coupling, but larger transient. The no SS model has not been parameterised to the same extent as the SS model – it is suggested to update the parameters with a new set or independent model before use. THE NON SS DYNAMICS MODEL HAS NOT BEEN VALIDATED AND TESTED. But the structure is there for future use. Chose whichever you prefer – the parameters provided should allow both models in their current state to work and be stable. Both reproduce Ca^{2+} transient alternans and waves, so ultimately a matter of preference which model you chose.

2. Copy this file to a file called "*Main.cc*". If using command line linux, type "make" to compile the code. Note: each file calls a different update parameters function, controlling settings for the different structures. These are in *lib/Params.c*, functions: *set_params_full_struct(..)* and *set_params_full_struct_no_SS(..)*. Ensure you modify the correct function!!

   If using Windows, you will have to create your own project in whatever software you use.

3. Now it is compiled, the code can be executed with "./model". This will run the model with default parameters. (BCL = 1000 ms; Cross-sectional portion of the cell).

4. This code uses command-line arguments to control parameters and settings. You may pass as many or few as you like, in any order. Each one is passed as an argument followed by a value, with spaces as separators.

   For example:

   ```
   ./model BCL 500 beats 10 RyR_Po 2
   ```

   Will run the model for 10 beats at a cycle length of 500 ms with the RyR open transition rate set to double the baseline value. The full list of available arguments is provided below.

To save time and memory, it is suggested that you place the "*Framework_geometry_and_state_files*" folder somewhere sensible, and specify its location in "*PATH.txt*". This way, the code will always look in this folder for geometry and state files, no matter which directory it is run from.

Full list of arguments

**Argument**	**Options**	**Notes**
BCL	x in ms	Basic cycle length
Total_time	x in ms	Total simulation time
Stim_period	x in ms	Period to apply stimulus
Beats	n	Number of applied beats
S2	x in ms	S2 stimulus interval
Ionic_model	"ORd"	Use this if adding different models
Celltype	"EPI"<br>"ENDO"<br>"M"	Selects celltype to run
Structure_model	"Idealised_small"<br>"Idealised_full"<br>"Cross_section" *default<br>"Full_cell"<br>"Full_cell_extended"<br>"Cross_section_idealised"<br>"Full_cell_idealised"	Selects structural datasets to use.<br>Cross_section_idealised uses real cytoplasm geometry but idealised dyads etc
Dyad_density	"Normal" *default<br>"High"	Only works for fully structural models; selects dyad map
Dss_scale	x [1 – 2.5]	Scales **D** for ss
red_factor	1, 2, 4	Factor by which SS is discretised relative to cyto
Spatial_output_interval	n (Default = 10)	Time-step to output spatial data
Cai	x (µM) (range 0.08 – 0.12)	Initial condition Cai
CaSR	x (µM) (range 600 – 1500)	Initial condition CaSR
RyR_Po	x	Scales open transition rate RyR
RyR_expression	x	Scales NRyR (per dyad)
LTCC_Po	x	Scales open rate LTCC
LTCC_expression	x	Scales NLTCC (per dyad)
Jup_scale	x	Scales J_{up}
Jleak_scale	x	Scales J_{leak}
Jmem_scale	x	Scales J_{NaCa}, J_{Cab}, J_{Cap}

Output files

Whole_cell_out contains files with average/global outputs from the simulation. *Spatial_diff_out* contains vtk and data files describing Ca^{2+} concentration in the cytoplasm and SR in the 3D volume of cell. vtk files may be visualised using software such as Paraview™.

The whole cell files are:

Currents.dat - contains current traces and gating variables

Excitation.dat - contains excitation measurement variables

CRU.dat - contains Ca handling data

RyR_states.dat - contains the RyR states

Ca_linescanZ.dat – linescan in Z direction for Ca

The full contents of the data-files are as follows:

File: “Whole_cell_out/Currents.dat”

**Column**	**Variable**
1	*Time (ms)*
2	*Vm (mV)*
3	I_{Na} *(pA/pF)*
4	I_{Na}*_voltage_activation_gate*
5	I_{Na}*_voltage_inactivation_gate_1*
6	I_{Na}*_voltage_inactivation_gate_2*
7	I_{to} *(pA/pF)*
8	I_{to}*_voltage_activation_gate*
9	I_{to}*_voltage_inactivation_gate*
10	I_{CaL} *(pA/pF)*
11	I_{CaL}*_voltage_activation_gate*
12	I_{CaL}*_voltage_inactivation_gate*
13	I_{Kur} *(pA/pF)*
14	I_{Kur}*_voltage_activation_gate*
15	I_{Kur}*_voltage_iactivation_gate*
16	I_{Kr} *(pA/pF)*
17	I_{Kr}*_voltage_activation_gate*
18	I_{Kr}*_voltage_iactivation_gate*
19	I_{K1} *(pA/pF)*
20	I_{NaCa} *(pA/pF)*
21	I_{NaCa_ss} *(pA/pF)*
22	I_{Cab} *(pA/pF)*
23	I_{Cap} *(pA/pF)*
24	I_{stim} *(pA/pF)*
25	I_{stim}*_S2 (pA/pF)*

File: "Whole_cell_out/Excitation.dat"

**Column**	**Variable**
1	*time*
2	*Vm*
3	*Excitation state (0 or 1)*
4	*dv/dt*
5	*dv/dt_max*
6	*APD*

File: "Whole_cell_out/CRU.dat"

**Column**	**Variable**
1	*Time*
2	*Vm*
3	$[Ca^{2+}]\_ds$
4	$[Ca^{2+}]\_ss$
5	$[Ca^{2+}]\_cyto$
6	$[Ca^{2+}]\_jSR$
7	$[Ca^{2+}]\_nSR$
8	J_{up}
9	J_{leak}
10	J_{CaL}
11	*N_RyR_open*
12	J_{rel}
13	J_{NaCa}
14	J_{NaCa_ss}
15	J_{Cab}
16	J_{Cab_ss}
17	J_{Cap}
18	J_{Cap_ss}
19	*Force*

File: "Whole_cell_out/RyR_states.dat"

**Column**	**Variable**
1	*time*
2	*C1*
3	*C2*
4	*O1 ** this is open state*
5	*O2*
6	*Monomer*
7	J_{rel}

Spatial data reconstruction and visualisation

VTK files may be directly visualised using appropriate software. The CaSR vtk output is of the 1D-strand map at the cytoplasm resolution. Please note that non-space is given a value of "-100" and therefore must be rendered as volume with transparency or thresholded. Please ensure to scale your colour bar from 0 (or higher) and not -100.

The model also outputs 1D array data files, which contain only the data associated with real space. These may be converted to vtks using post-processing (simple code provided) and require the geometry. This is what you need to do if you want the visualisation of the high-resolution SR concentration.

Two post-processing tools have been provided, but are not fully tidied or nearly as functionally-neat as the main code. Check for updates on these tools.

Make_cropped_vtk_from_cross_section.c - takes in the 1D data array from the cross-section, and outputs a VTK of a quarter-portion. Use as such

./exe [Ca, CaSR] [time] [geometry file]

For example ./exe Ca 30 [PATH]/Intracellular_structure_geometries/CYTO_cross_section.geom

Will produce a vtk for time output 30. You can update this code to take in full-length geometry.

Make_SR_VTK_files_cropped_from_cross_section.c - makes the full resolution visualisation of the SR in a quarter portion from the cross section data

./exe [time] [PATH]/Intracellular_structure_geometries/SR_1D_cross_section.geom

NOTE: the file "SR_full_geo_map.map" (found in the geometries folder) must be in the current directory, or re-coded to read it properly. Note that this code needs the 1D geometry to relate the array to 3D space, then the full res geometry to map it to high res.

Adding your own structural datasets

The code has been designed for ease of inclusion of new structural datasets.

You must provide a “cytoplasm” dataset (0s for non-space and 1s for space, corresponding to the intracellular volume of the cell). Everything else can be idealised or use geometries, depending on what data you have

Files to include:

Cytoplasm geometry

Membrane and TT geometry

SR geometry

Dyad map

All geometries must be in the form of a grid of 0s and 1s. All grids must be the same size, and correspond to the discretisation resolution you are using. If this is done, the mapping functions between geometries is all taken care of in the code.

Go to *lib/Intracellular_structure.cpp* and find the function “*set_structure_geometry_parameters*(…)”

Simply add your own section to the *if* statements – call your model whatever you like, specific the dimension sizes and specify your geometry names. Use the cytoplasm geometry for any geometry you don’t have data for (except the dyad distribution – set it to “None” if no data and it will create a uniform distribution for you). It should be clear how to do this – just follow the forms of the options already there.

Adding new dynamics models

The model dynamics can be largely controlled by the model parameters, and so these can be used to develop new or integrate previously existing models. In “*lib/Params.c*” has been provided a function “*update_default_params* (…)”. You may use this to add new parameter values for a new model, without changing the default model. Please ensure the function is called in Main (it is currently commented out).

If you need to change equations, please create new functions and change which function is called.

PLEASE FEEL FREE TO CONTACT ME WITH ANY QUESTIONS OR ISSUES.

SIMILARLY, IF YOU USE THIS CODE AND BUILD ON IT, I WOULD APPRECIATE BEING SENT ANY UPDATED VERSIONS.

Simulation settings examples

Just a few examples of how to produce different behaviour:

Control pacing, cross sectional cell portion (default settings)

./model

Control pacing, full length cell model

./model Structure_model Full_cell

Control pacing, idealised model

./model Structure_model Idealised_small

./model Structure_model Idealised_full

Control pacing, semi-idealised

./model Structure_model Semi_idealised_small

./model Structure_model Semi_idealised_full

Alternans (experiment with scaling/BCL)

./model Jup_scale [0.3-0.7] LTCC_Po [0.3-0.7] BCL [340-550] + Structure arguments

Ca^{2+} spark hierarchy (try all of these settings, see what happens) | use also Structure_model Full_cell for proper waves (but longer simulations)

./model CaSR [800-1500] Beats 0 Total_time 2000

./model CaSR [800-1500] Beats 0 Total_time 2000 red_factor 4

./model CaSR [800-1500] Beats 0 Total_time 2000 Dyad_density High

./model CaSR [800-1500] Beats 0 Total_time 2000 red_factor 4 Dyad_density High

./model CaSR [800-1500] Beats 0 Total_time 2000 Dyad_density High RyR_expression 1.5

./model CaSR [800-1500] Beats 0 Total_time 2000 red_factor 4 Dyad_density High RyR_expression 1.5

Rapid pacing (try running this on cross-sectional model, then input Cai and CaSR from that data in full cell model for 3-5 beats and see what happens)

./model ISO 1 BCL 400 Jmem_scale [1-3]
