# Supplementary material for: A computational model of spatio-temporal cardiac intracellular calcium handling with realistic structure and spatial flux distribution from sarcoplasmic reticulum and t-tubule reconstructions
Source: PLoS Comput Biol. 2017 Aug 31;13(8):e1005714. doi: 10.1371/journal.pcbi.1005714 (PMC5597258; doi:10.1371/journal.pcbi.1005714)
Supplement: S1 Text — Contains all equations, variables and parameters describing the developed model. (PDF) [file pcbi.1005714.s002.pdf]

Supporting Information S1 - Model Description

A Computational Model of Spatio-Temporal Cardiac Intracellular Calcium Handling with Realistic Structure and Spatial Flux Distribution from Sarcoplasmic Reticulum and T-tubule Reconstructions

Michael A. Colman*[1,3], Christian Pinali[2], Andrew W. Trafford[2], Henggui Zhang[3] and Ashraf Kitmitto[2]

[1] School of Biomedical Sciences, Faculty of Biological Sciences, University of Leeds, UK

[2] Division of Cardiovascular Sciences, Faculty of Biology, Medicine and Health Sciences, University of Manchester, UK

[3] School of Physics and Astronomy, Faculty of Engineering and Physical Sciences, University of Manchester, UK

* Corresponding author

E-mail: **m.a.colman@leeds.ac.uk** (MAC)

Contents

Variables 3
Cell Structure 4
Calcium Dynamics 4
- Diffusively coupled domains (cyto, rbSS, nSR) 4
- Dyad and jSR 5
- Inter-domain diffusion 5

Ca^{2+} Buffering 6
- Instantaneous buffering in the cytoplasm 6
- Instantaneous buffering in the jSR 6
- Troponin buffering and force 6

Reaction terms and flux definitions 9
- Dyad and jSR 9
  - Intracellular Ca^{2+} release, J_{rel} 9
  - L-type Calcium Flux, J_{CaL} 10
- SR fluxes 13
  - Intracellular uptake through SERCA 13
  - SR Ca^{2+} leak 13
- Membrane Fluxes 14
  - Sodium-Ca^{2+}-exchanger, J_{NaCa} 14
  - Membrane Ca^{2+} pump, J_{pCa} 14
  - Background Ca^{2+} current 14
- Complete Equations for the Reaction Terms 15

Ionic Model 15
Initial Conditions 18
References 19

Variables

Table S1: Ca^{2+} handling model variables

Parameter	Description	Unit
$[Ca^{2+}]_{ds}$	Average Ca^{2+} concentration in the dyadic cleft	µM
$^{m}[Ca^{2+}]_{ds}$	Ca^{2+} concentration in dyad *m*	µM
$[Ca^{2+}]_{rbSS}$	Average Ca^{2+} concentration in the subspace	µM
$^{n}[Ca^{2+}]_{rbSS}$	Ca^{2+} concentration in subspace voxel *n*	µM
$[Ca^{2+}]_{cyto}$	Average Ca^{2+} concentration in the bulk cytoplasm	µM
$^{n}[Ca^{2+}]_{cyto}$	Ca^{2+} concentration in cytoplasm voxel *n*	µM
$[Ca^{2+}]_{nSR}$	Average Ca^{2+} concentration in the network SR	µM
$^{q}[Ca^{2+}]_{nSR}$	Ca^{2+} concentration in network SR voxel *q*	µM
$[Ca^{2+}]_{jSR}$	Average Ca^{2+} concentration in the junctional SR	µM
$^{m}[Ca^{2+}]_{jSR}$	Ca^{2+} concentration in junctional SR *m*	µM
$^{m}J_{rel}$ | J_{rel}	Release flux in dyad *m* | Whole cell average release flux	$µM.ms^{-1}$
$^{m}J_{CaL}$ | I_{CaL}	LTCC flux in dyad *m* | Whole cell current	$µM.ms^{-1}$ | pA/pF
$^{q}J_{up}$ | J_{up}	SR Uptake flux in voxel *q* | Whole cell SR update flux	$µM.ms^{-1}$
$^{q}J_{leak}$ | J_{leak}	SR leak flux in voxel *q* | Whole cell SR leak flux	$µM.ms^{-1}$
$^{m}J_{ds}$	Flux from dyad *m* to rbSS voxel at *m*	$µM.ms^{-1}$
$^{n}J_{ss}$	Flux from rbSS to cytoplasm voxel *n*	$µM.ms^{-1}$
$^{m}J_{jsr}$	Flux from network to junctional SR at dyad *m*	$µM.ms^{-1}$
$^{n}J_{trpn}$	Trpn buffering flux at voxel *n*	$µM.ms^{-1}$
$^{p}J_{NaCa}$ | I_{NaCa}	Sodium-Ca^{2+} exchanger flux in voxel *p* | Whole cell current	$µM.ms^{-1}$ | pA/pF
$^{p}J_{pCa}$ | I_{pCa}	PMCA Ca^{2+} pump flux in voxel *p* | Whole cell current	$µM.ms^{-1}$ | pA/pF
J_{Cab} | I_{Cab}	Background Ca^{2+} current flux in voxel *p* | Whole cell current	$µM.ms^{-1}$ | pA/pF
$^{n}\beta_{cyto}$	Instantaneous buffering in the cytoplasm, voxel *n*	-
$^{m}\beta_{jSR}$	Instantaneous buffering in the junctional SR, dyad *m*	-
$^{n}H_{trpn}$	Ca^{2+} bound to high affinity troponin sites, voxel *n*	µM
$^{n}L_{trpn}$	Ca^{2+} bound to high affinity troponin sites, voxel *n*	µM
$^{m}n_{o\_ryr}$	Number of open RyRs in dyad *m*	-
^{m}CA	Number of RyRs in the Closed Activated state, dyad *m*	-
^{m}OA	Number of RyRs in the Open Activated state, dyad *m*	-
^{m}CI	Number of RyRs in the Closed Inactivated state, dyad *m*	-
^{m}OI	Number of RyRs in the Open Inactivated state, dyad *m*	-
csqn	Free calsequestrin concentration	mM
^{m}M	Proportion of csqn in monomer state, dyad *m*	-
$^{m}d_{1}$	LTCC activation gate state 1, dyad *m*	-
$^{m}d_{2}$	LTCC activation gate state 2, dyad *m*	-
$^{m}d_{3}$	LTCC activation gate state 3, dyad *m*	-
$^{m}f_{1}$	LTCC voltage-dependent inactivation state 1, dyad *m*	-
$^{m}f_{2}$	LTCC voltage-dependent inactivation state 2, dyad *m*	-
$^{m}fca_{1}$	LTCC Ca^{2+}-dependent inactivation state 1, dyad *m*	-
$^{m}fca_{2}$	LTCC Ca^{2+}-dependent inactivation state 2, dyad *m*	-
V_{m}	Membrane potential	mV

Cell Structure

Table S2: Cell and voxel volumes and dimensions

Parameter	Description	Value
v_{cyto}	Total volume cytoplasm	49 695 µm^3
v_{cyto_vox}/v_{vox}	Volume per voxel cytoplasm	3.137 x10^{-2} µm^3
v_{nsr}	Total volume network SR	1 826 µm^3
v_{nsr_vox}	Volume per voxel nSR (1D strand map)	2.987 x 10^{-3} µm^3
v_{rbSS}	Total volume of sub-space*	356 – 535 µm^3
v_{rbSS_vox}	Volume per voxel sub-space**	2.703 x10^{-3} – 1.2825 x 10^{-2} µm^3
$<v_{ds}>$	Average volume of individual dyad***	1.512 x10^{-3} µm^3
v_{jSR}	Volume individual jSR	3.5 x10^{-2} µm^3
N_{cyto}	Number of voxels associated with the cytoplasm	1 584 408
N_{nSR}	Number of voxels associated with the nSR	611 404
N_{MEM}	Number of voxels associated with the surface sarcolemma and T-tubules	463 624
N_{Dyads}	Number of dyads / jSRs	17 458

** Volume is varied between simulations*
*** Dependant on discretisation resolution of subspace*
**** Varied between individual dyads within a single simulation*
Total volume and dimensions are provided for the full-length cell model

Calcium Dynamics

This section gives the fundamental equations describing Ca^{2+} dynamics in each of the five domains (cytoplasm, reduced buffering subspace, dyadic cleft, network and junctional SR).

Diffusively coupled domains (cyto, rbSS, nSR)

In the three diffusively coupled domains, dynamics of Ca^{2+} concentration is described by the isotropic reaction diffusion equation:

$$\frac{d[Ca^{2+}]_{cyto,rbSS,nSR}}{dt} = \beta_{cyto,rbSS,nSR}\left(\mathbf{D}\nabla^2[Ca^{2+}]_{cyto,rbSS,nSR} + \phi_{cyto,rbSS,nSR}\right) \quad (1)$$

Where Φ is a general reaction term, β is the instantaneous buffering term (β_{cyto} given below and where $\beta_{nSR,rbSS} = 1$), ∇^2 is the spatial laplacian operator in 3-D and $\boldsymbol{D}$ is the diffusion coefficient. At each voxel, $n = 1, 2 \ldots N_{cyto}$, the laplacian is approximated by the 6 node nearest neighbour finite difference method:

$$\mathbf{D}\nabla^2.{}^{n}[Ca^{2+}]_{cyto,rbSS,nSR} \approx \sum_{x=x_1}^{x=x_3} \mathbf{D}\left(\frac{{}^{n_{x+1}}[Ca^{2+}]_{cyto,rbSS,nSR} + {}^{n_{x-1}}[Ca^{2+}]_{cyto,rbSS,nSR} - 2.{}^{n}[Ca^{2+}]_{cyto,rbSS,nSR}}{\Delta x^2}\right) \quad (2)$$

Where Δx is the spatial discretisation step, x_1-x_3 are the three spatial dimensions and $n_{x\pm1}$ refers to each of the 2 neighbours in each dimension. In the cytoplasm and rbSS domains these correspond to the standard 6 nearest-neighbours (i.e., ± Δx in the x, y and z directions); in the nSR for the full structural model, these are defined by the neighbourhood map from the full resolution reconstruction.

Dyad and jSR

For each dyad and jSR, where *m* is the subset of voxels which contain a dyad (*m = 1, 2 ... N_{dyads}*):

$$\frac{d\,{}^{m}\left[Ca^{2+}\right]_{ds}}{dt} = \beta\left({}^{m}J_{rel} + {}^{m}J_{CaL} - {}^{m}J_{ds}\right) \tag{3}$$

$$\frac{d\,{}^{m}\left[Ca^{2+}\right]_{jSR}}{dt} = \beta\left(-{}^{m}J_{rel}\left(\frac{v_{ds}}{v_{jSR}}\right) - {}^{m}J_{jSR}\right) \tag{4}$$

Fluxes J_{rel} and J_{CaL} are given in the section *Reaction Terms and Flux Definition* and J_{ds} and J_{jSR} given in the next sub-section; all are dependent on local Ca^{2+} concentrations.

Due to the small volume of the dyadic cleft, an analytical description can be found for the dyadic cleft Ca^{2+} concentration under the approximation that the volume reaches its steady-state concentration within the time-step, *Δt*. Thus, by setting:

$$\frac{d\left[Ca^{2+}\right]_{ds}}{dt} = 0 \tag{5}$$

An approximation for equation (3) can be obtained as [1]:

$${}^{m}\left[Ca^{2+}\right]_{ds} = {}^{\theta(m)}\left[Ca^{2+}\right]_{rbSS} + \frac{\tau_{ds}.\left({}^{m}k_{rel}.\,{}^{m}\left[Ca^{2+}\right]_{jSR} + {}^{m}J_{CaL}\right)}{\left(1+\tau_{ds}.\,{}^{m}k_{rel}\right)} \tag{6}$$

Where *θ(m)=n* is the dyad mapping function (inverse map $\theta^{-1}(n)=m$). k_{rel} is given in the sub-section *Reaction Terms and Flux Definition*: *Dyad Fluxes*.

Inter-domain diffusion

Free Ca^{2+} diffusion occurs between the dyadic cleft and rbSS, the rbSS and bulk cytoplasm, and the network and junctional SR [2].

Dyadic cleft to rbSS:

$${}^{m}J_{ds} = \left({}^{m}\left[Ca^{2+}\right]_{ds} - {}^{\theta(m)}\left[Ca^{2+}\right]_{rbSS}\right)\tau_{ds}^{-1} \tag{7}$$

rbSS to cytoplasm:

$${}^{n}J_{SS} = \left({}^{n}\left[Ca^{2+}\right]_{rbSS} - {}^{n}\left[Ca^{2+}\right]_{cyto}\right)\tau_{ss}^{-1} \tag{8}$$

Network to junctional SR:

$${}^{m}J_{jSR} = \left({}^{m}\left[Ca^{2+}\right]_{jSR} - {}^{\theta(m)}\left[Ca^{2+}\right]_{nSR}\right)\tau_{jSR}^{-1} \tag{9}$$

Table S3: Free Ca^{2+} diffusion parameters

Parameter	Description	Value
D	Diffusion coefficient (cytoplasm, nSR)	0.3 µm/ms
D_{rbSS}	Diffusion coefficient (rbSS)*	0.3 – 1.0 µm/ms
Δx	Spatial discretisation step	0.35 µm
Δx_{rbSS}	Spatial discretisation step (rbSS)**	0.35 – 1.4 µm
τ_{ds}	Time constant diffusion dyad → rbSS	0.022 ms
τ_{ss}	Time constant diffusion rbSS → cytoplasm	0.1 ms
τ_{jSR}	Time constant diffusion jSR → nSR	5 ms

** Varied between simulations*
*** Dependant on discretisation resolution*

Ca^{2+} Buffering

Instantaneous buffering in the cytoplasm

Instantaneous buffering in the cytoplasm follows that of previous models, e.g., Restrepo et al [2] and Nivala et al. [3], based on [4]. At each voxel, *n*, the buffering term is given by:

$$ {}^{n}\beta_{cyto} = \left[1 + \sum_{x} \frac{B_x K_x}{\left({}^{n}[Ca^{2+}]_{cyto} + K_x \right)^2} \right]^{-1} \tag{10} $$

Where *x* refers to four buffering processes: Calmodulin, SR sites, Myosin (Ca) and Myosin (Mg).

Instantaneous buffering in the jSR

Buffering in the jSR follows that of the previous study Gaur-Rudy [5]:

$$ {}^{m}\beta_{jSR} = \left[1 + \frac{B_{csqn} K_{mcsqn}}{\left({}^{m}[Ca^{2+}]_{jSR} + K_{mcsqn} \right)^2} \right]^{-1} \tag{11} $$

Troponin buffering and force

Troponin buffering and force generation is from the Gauthier et al model [6,7]:

$$ {}^{n}J_{trpn} = \frac{dH_{trpn,Ca}}{dt} + \frac{dL_{trpn,Ca}}{dt} \tag{12} $$

$$ \frac{d\,{}^{n}H_{trpn,Ca}}{dt} = k^{+}_{H,trpn}\,{}^{n}\left[Ca^{2+}\right]_{cyto} \left(B_{H,trpn} - H_{trpn,Ca} \right) - k^{-}_{H,trpn} H_{trpn,Ca} \tag{13} $$

$$ \frac{d\,{}^{n}L_{trpn,Ca}}{dt} = k^{+}_{L,trpn}\,{}^{n}\left[Ca^{2+}\right]_{cyto} \left(B_{L,trpn} - L_{trpn,Ca} \right) - k^{-}_{L,trpn} \left(1 - \frac{2}{3} F_{norm} \right) L_{trpn,Ca} \tag{14} $$

Where F_{norm} is the normalised force:

$$ F_{norm} = \left(\frac{P_1 + N_1 + 2P_2 + 3P_3}{P_1^{\max} + 2P_2^{\max} + 3P_3^{\max}} \right) \tag{15} $$

And:

$$\phi = 1 + \frac{2.3 - SL}{(2.3 - 1.7)^{1.6}} \tag{16}$$

$$f_{01} = 3 f_{XB} \tag{17}$$

$$f_{12} = 10 f_{XB} \tag{18}$$

$$f_{23} = 7 f_{XB} \tag{19}$$

$$g_{01} = g_{XB} \tag{20}$$

$$g_{12} = 2 g_{XB} \tag{21}$$

$$g_{23} = 3 g_{XB} \tag{22}$$

$$g_{01,SL} = 1 \phi g_{XB,\min} \tag{23}$$

$$g_{12,SL} = 2 \phi g_{XB,\min} \tag{24}$$

$$g_{23,SL} = 3 \phi g_{XB,\min} \tag{25}$$

$$K_{TRPN}^{Ca} = \frac{k_{L,TRPN}^{-}}{k_{L,TRPN}^{+}} \tag{26}$$

$$N_{TRPN} = 3.5 SL - 2.0 \tag{27}$$

$$K_{1/2}^{TRPN} = \left[1 + \frac{K_{TRPN}^{Ca}}{1.4 \times 10^{-3} - 8 \times 10^{-4} \left((SL - 1.7)/0.6 \right)} \right]^{-1} \tag{28}$$

$$k_{np,TRPN} = k_{pn,TRPN} \left(\frac{\left[TRPN_{Ca}^{L} \right]}{K_{1/2} TRPN_{tot}^{L}} \right)^{N_{TRPN}} \tag{29}$$

$$\sum Paths = g_{01} g_{12} g_{23} + f_{01} g_{12} g_{23} + f_{01} f_{12} g_{23} + f_{01} f_{12} f_{23} \tag{30}$$

$$P_1^{\max} = \frac{f_{01} g_{12} g_{23}}{\sum Paths} \tag{31}$$

$$P_2^{\max} = \frac{f_{01} f_{12} g_{23}}{\sum Paths} \tag{32}$$

$$P_3^{\max} = \frac{f_{01} f_{12} f_{23}}{\sum Paths} \tag{33}$$

$$\frac{dP_0}{dt} = -\left(k_{pn,TRPN} + f_{01}\right)P_0 + k_{np,TRPN}N_0 + g_{01,SL}P_1 \quad (34)$$

$$\frac{dP_1}{dt} = -\left(k_{pn,TRPN} + f_{12} + g_{01,SL}\right)P_1 + k_{np,TRPN}N_1 + f_{01}P_0 + g_{12,SL}P_1 \quad (35)$$

$$\frac{dP_2}{dt} = -\left(f_{23} + g_{12,SL}\right)P_2 + f_{12}P_1 + g_{23,SL}P_3 \quad (36)$$

$$\frac{dP_3}{dt} = -g_{23,SL}P_3 + f_{23,SL}P_2 \quad (37)$$

$$\frac{dN_1}{dt} = k_{pn,TRPN}P_1 - \left(k_{np,TRPN} + g_{01,SL}\right)N_1 \quad (38)$$

$$\frac{dN_0}{dt} = \frac{dP_0}{dt} - \frac{dP_1}{dt} - \frac{dP_2}{dt} - \frac{dP_3}{dt} - \frac{dN_1}{dt} \quad (39)$$

Table S4: Ca^{2+} buffering parameters

Parameter	Description	Value
K_{CAM}	Dissociation constant for calmodulin	7.0 µM
B_{CAM}	Total concentration buffering sites	24.0 µM
K_{SR}	Dissociation constant for SR sites	1.08 µM
B_{SR}	Total concentration buffering sites	47.0 µM
$K_{M,Ca}$	Dissociation constant for Myosin (Ca)	0.01155 µM
$B_{M,Ca}$	Total concentration buffering sites	49.0 µM
$K_{M,Mg}$	Dissociation constant for Myosin (Mg)	6.552 µM
$B_{M,Mg}$	Total concentration buffering sites	140 µM
K_{mcsqn}	Dissociation constant for csqn	0.8 mM
B_{csqn}	Total concentration buffering sites	10 mM
$k^{+}_{H, trpn}$	On rate for troponin high affinity sites	100 mM^{-1} ms^{-1}
$k^{-}_{H, trpn}$	Off rate for troponin high affinity sites	1.0 x10^{-3} ms^{-1}
$k^{+}_{L, trpn}$	On rate for troponin low affinity sites	100 mM^{-1} ms^{-1}
$k^{-}_{L, trpn}$	Off rate for troponin low affinity sites	4.0 x 10^{-2} ms^{-1}
$B_{H,trpn}$	Total high affinity sites on troponin	0.14 mM
$B_{L,trpn}$	Total low affinity sites on troponin	0.7 mM
f_{XB}	Weak to strong cross bridge transition rate	0.05 ms^{-1}
$g_{XB,min}$	Maximum strong to weak rate	0.1 ms^{-1}
SL	Sarcomere length	2.15 µm
$K_{pn,TRPN}$	Permissive to non-permissive transition rate	0.04 ms^{-1}

Reaction terms and flux definitions

Thus far, only the contributions of free Ca^{2+} diffusion to the reaction terms have been defined. In this section, the full reaction terms and their governing equations are described.

Dyad and jSR

Other than free Ca^{2+} diffusion, the only fluxes acting into the dyad are J_{rel} and J_{CaL} described by the RyR and LTCC models, respectively.

Intracellular Ca^{2+} release, J_{rel}

$$ {}^{m}J_{rel} = {}^{m}k_{rel}\left({}^{m}\left[Ca^{2+}\right]_{jSR} - {}^{m}\left[Ca^{2+}\right]_{ds} \right) \tag{40}$$

$$ {}^{m}k_{rel} = {}^{m}n_{o\_RyR}.g_{RyR}.{}^{m}v_{ds}^{-1} \tag{41}$$

${}^{m}n_{o\_RyR}$ is the number of open RyR channels in dyad m. RyR dynamics is described by a 4-state Markov Chain model. The model is similar to Stern et al [8] and Restrepo et al [2], with a functional monomer induced inactivation based on csqn dynamics described by Gaur-Rudy [5].

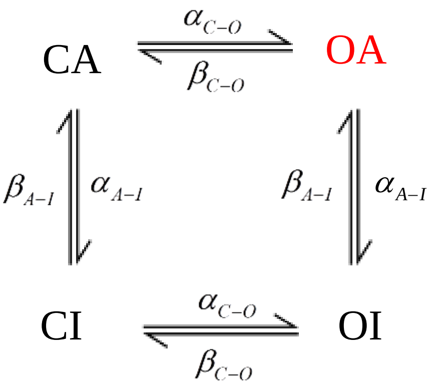

$$ \frac{d\,{}^{m}CA}{dt} = {}^{m}OA.\beta_{C-O} + {}^{m}CI.\beta_{A-I} - {}^{m}CA.(\alpha_{C-O} + \alpha_{A-I}) \tag{42}$$

$$ \frac{d\,{}^{m}OA}{dt} = {}^{m}CA.\alpha_{C-O} + {}^{m}OI.\beta_{A-I} - {}^{m}OA.(\beta_{C-O} + \alpha_{A-I}) \tag{43}$$

$$ \frac{d\,{}^{m}CI}{dt} = {}^{m}OI.\beta_{C-O} + {}^{m}C.\alpha_{A-I} - {}^{m}CI.(\alpha_{C-O} + \beta_{A-I}) \tag{44}$$

$$ \frac{d\,{}^{m}OI}{dt} = {}^{m}CI.\alpha_{C-O} + {}^{m}OA.\alpha_{A-I} - {}^{m}OI.(\beta_{C-O} + \beta_{A-I}) \tag{45}$$

Where:

$$ \alpha_{C-O} = k_{a}\left({}^{m}\left[Ca^{2+}\right]_{ds} \right)^{H} \tag{46}$$

$$\beta_{C-O} = k_b \tag{47}$$

$$\alpha_{A-I} = \left(1 - {}^{m}Mi_{ss}\right) / \tau_{Mi,1} \tag{48}$$

$$\beta_{A-I} = {}^{m}Mi_{ss} / \tau_{Mi,2} \tag{49}$$

$${}^{m}Mi_{ss} = 1 \Big/ \left(1 + e^{({}^{m}M - 0.5)/0.04167}\right) \tag{50}$$

$$\frac{dM}{dt} = \alpha_M (1 - M) + \beta_M M \tag{51}$$

$$\alpha_M = M_{ss} / \tau_{M,1} \tag{52}$$

$$\beta_M = (1 - M_{ss}) \big/ \tau_{M,2} \tag{53}$$

$$M_{ss} = 1 \big/ (1 + e^{(-6.5.(csqn - 6.37))}) \tag{54}$$

$$csqn = B_{csqn}.K_{mcsqn} \Big/ \left({}^{m}\left[Ca^{2+}\right]_{jSR} + K_{mcsqn}\right) \tag{55}$$

In this model, *OA* is the only state in which a flux occurs. Thus, ${}^{m}n_{o\_RyR}$ is equal to the number of channels in dyad *m* which are in state *OA* (red text in schematic).

L-type Calcium Flux, J_{CaL}

The flux through the L-type Calcium Current is defined as:

$${}^{m}J_{CaL} = -{}^{m}n_{o\_LTCC} \, {}^{m}\bar{J}_{CaL} \tag{56}$$

Where ${}^{m}n_{o\_LTCC}$ is the number of open LTCC channels in dyad *m* (defined below) and $\bar{J}_{CaL}$ is the maximal flux rate per channel [9]:

$${}^{m}\bar{J}_{CaL} = 4P_{Ca}zF \, \frac{\frac{1}{2}\gamma_{Ca} \, {}^{m}\left[Ca^{2+}\right]_{ds} e^{2z} - \gamma_{Ca}\left[Ca^{2+}\right]_{o}}{e^{2z} - 1} \tag{57}$$

$$z = \frac{V_m F}{RT} \tag{58}$$

Where $[Ca^{2+}]_o$ is the extracellular Ca^{2+} concentration, P_{Ca} is the maximum permeability of an individual LTCC and *F* is the Faraday constant.

The LTCCs are described by a Markov Chain construction of a Hodkin-Huxley model [9,10]

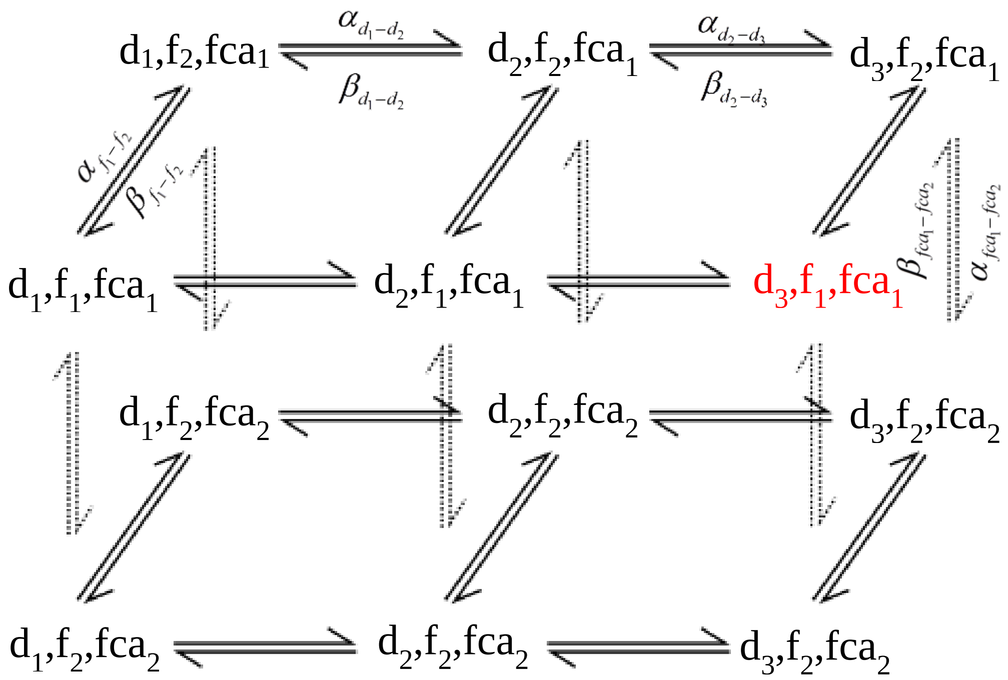

Which is equivalent to the three gate Hodgkin-Huxley model:

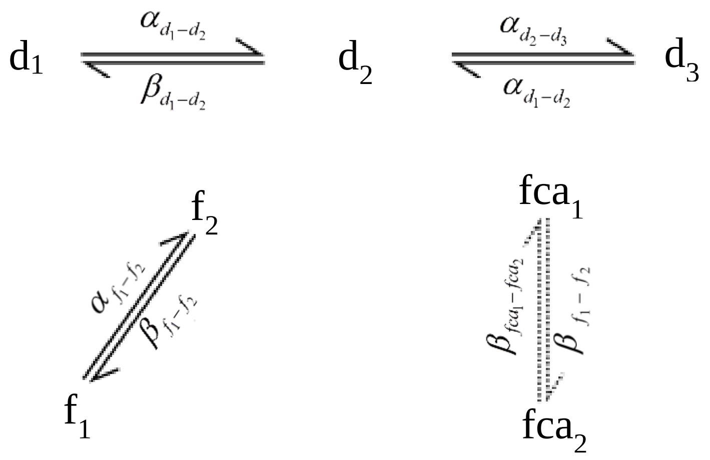

And thus described by:

$$\frac{d(d_1)}{dt} = d_2\beta_{d_1-d_2} - d_1\alpha_{d_1-d_2} \tag{59}$$

$$\frac{d(d_2)}{dt} = d_1\alpha_{d_1-d_2} + d_3\beta_{d_2-d_3} - d_2(\beta_{d_1-d_2} + \alpha_{d_2-d_3}) \tag{60}$$

$$\frac{d(d_3)}{dt} = d_2\alpha_{d_2-d_3} - d_3\beta_{d_2-d_3} \quad (61)$$

$$\frac{d(f_1)}{dt} = f_2\beta_{f_1-f_2} - f_1\alpha_{f_1-f_2} \quad (62)$$

$$\frac{d(fca_1)}{dt} = fca_2\beta_{fca_1-fca_2} - fca_1\alpha_{fca_1-fca_2} \quad (63)$$

Where the transition rates for each variable couplet, ($x = d_1\text{-}d_2$, $f_1\text{-}f_2$, $fca_1\text{-}fca_2$) are defined from the steady-state and time constant in the standard way:

$$\alpha_x = x_{ss} / \tau_x \quad (64)$$

$$\beta_x = (1 - x_{ss}) / \tau_x \quad (65)$$

And:

$$\alpha_{d_2-d_3=}k_{d2d3} \quad (66)$$

$$\beta_{d_2-d_3=}k_{d3d2} \quad (67)$$

$$d_{ss} = 1 \Big/ \left(1 + e^{(-(V_m-5)/6.24)}\right) \quad (68)$$

$$\tau_d = d_{ss}.\left(1 - e^{(-(V_m-5)/6.24)}\right) \Big/ \left(0.035(V_m - 5)\right) \quad (69)$$

$$f_{ss} = 1 - 1 \Big/ \left(1 + e^{((V_m+32.06)/8.6)}\right) \quad (70)$$

$$\tau_f = 2 \Bigg/ \left(0.0197e^{-\left(\left[0.0337(V_m+7)\right]^2+0.02\right)}\right) \quad (71)$$

$$fca_{ss} = 1 - 1 \Bigg/ \left(1 + \left({}^{m}\left[Ca^{2+}\right]_{ds} \Big/ \bar{C}a\right)^2\right) \quad (72)$$

Note that the steady states of the inactivation gates (*f, fca*) are inverse to those in the standard Hodgkin-Huxley model because in this Markov description f_2 is the inactivated state, equivalent to ($1\text{-}f$) in the standard description (and f_1 is equivalent to f).

Table S5: RyR and LTCC flux parameters

Parameter	Description	Value
g_{ryr}	Maximal flux rate through the RyRs	2.05 x 10^{-4} μm^3 ms^{-1}
P_{Ca}	Maximum permeability of LTCC	11.9 µmol C^{-1} ms^{-1}
γ_{Ca}	Activity Coefficient LTCC	0.341
N_{RyR}	Number of RyRs per dyad*	100
H	RyR Open rate Ca^{2+} power	2.5
k_a	RyR activation rate constant	0.617 x 10^{-4} $\mu M^{-2.5}$ ms^{-1}
k_b	RyR deactivation rate constant	1.0 ms^{-1}
$\tau_{M,1}$	Time constant of monomer binding	5 ms
$\tau_{Mi,1}$	Time constant of monomer inactivation	5 ms
$\tau_{M,2}$	Time constant monomer unbinding	213 ms
$\tau_{Mi,2}$	Time constant of de-inactivation	30 ms
N_{LTCC}	Number of L-type Ca^{2+} channels per dyad*	15
k_{d2d3}	Rate constant for transition d_2-d_3	0.3 ms^{-1}
k_{d3d2}	Rate constant for transition d_3-d_2	6.0 ms^{-1}
τ_{fca}	Time constant for Ca^{2+} induced inactivation	15 ms
$\bar{Ca}$	Ca^{2+} constant for Ca^{2+} induced inactivation	6.0 µM
$[Ca^{2+}]_o$	Extracellular Ca^{2+} concentration	1.8 mM

**Can be varied between individual dyads and simulations*

SR fluxes

Flux between the SR and the cytoplasm are J_{up} and J_{leak}, where q is the subset of cytoplasm voxels which contain an SR ($q = 1, 2 \ldots N_{SR}$). These equations are based on Restrepo et al [2] and preceding studies [11,12].

Intracellular uptake through SERCA

$$ {}^{q}J_{up} = g_{up} \frac{\left({}^{\theta_{SR}(q)}\left[Ca^{2+}\right]_{cyto} \big/ K_{cyto} \right)^2 - \left({}^{q}\left[Ca^{2+}\right]_{nSR} \big/ K_{nSR} \right)^2}{1 + \left({}^{\theta_{SR}(q)}\left[Ca^{2+}\right]_{cyto} \big/ K_{cyto} \right)^2 + \left({}^{q}\left[Ca^{2+}\right]_{nSR} \big/ K_{nSR} \right)^2} \quad (73) $$

Where $\theta_{SR}(q)=n$ is the SR mapping function (inverse map $\theta_{SR}^{-1}(n)=q$) and g_{up} is the maximal flux rate.

SR Ca^{2+} leak

$$ {}^{q}J_{leak} = g_{leak} \frac{{}^{q}\left[Ca^{2+}\right]_{nSR}^2}{{}^{q}\left[Ca^{2+}\right]_{nSR}^2 + K_{leak}^2} \left({}^{q}\left[Ca^{2+}\right]_{nSR} - {}^{\theta_{SR}(q)}\left[Ca^{2+}\right]_{cyto} \right) \quad (74) $$

Table S6: Ca^{2+} uptake and leak parameters

Parameter	Description	Value
g_{up}	Maximal flux rate of J_{up}	0.12161 µM.ms^{-1}
K_{cyto}	Cytoplasm constant for J_{up}	0.15 µM
K_{nSR}	Network SR constant for J_{up}	1700 µM
g_{leak}	Maximal flux rate of J_{leak}	1.28422 x10^{-5} ms^{-1}
K_{leak}	J_{leak} constant	450 µM

Membrane Fluxes

Fluxes for each voxel p ($p = 1, 2 \ldots N_{MEM}$) corresponding to the surface sarcolemma and T-tubules are defined for the sodium-Ca^{2+}-exchanger (J_{NaCa}), membrane Ca^{2+} pump (J_{pCa}) and background Ca^{2+} current (J_{Cab}). Membrane voxel p is associated with cytoplasm voxel n by the membrane mapping function $\theta_{MEM}(p)=n$ and inverse map $\theta_{MEM}^{-1}(n)=p$.

Sodium-Ca^{2+}-exchanger, J_{NaCa}

$$^{p}J_{NaCa} = \frac{K_a g_{NaCa} v_{vox}^{-1}\left(e^{\eta z}\left[Na^+\right]_i\left[Ca^{2+}\right]_o - e^{(\eta-1)z}\left[Na^+\right]_o^{\ \theta_{MEM}(p)}\left[Ca^{2+}\right]_{cyto}\right)}{(t_1+t_2+t_3)\left(1+K_{sat}e^{(\eta-1)z}\right)} \tag{75}$$

Where

$$t_1 = K_{mCai}\left[Na^+\right]_o^3\left(1+\left(\left[Na^+\right]_i \middle/ K_{mNai}\right)^3\right) \tag{76}$$

$$t_2 = K_{mNao}^3 \ {}^{\theta_{MEM}(p)}\left[Ca^{2+}\right]_{cyto}\left(1+\left({}^{\theta_{MEM}(p)}\left[Ca^{2+}\right]_{cyto} \middle/ K_{mCai}\right)\right) \tag{77}$$

$$t_3 = K_{mCao}\left[Na^+\right]_i^3 + \left[Na^+\right]_i^3\left[Ca^{2+}\right]_o + \left[Na^+\right]_o^3 \ {}^{\theta_{MEM}(p)}\left[Ca^{2+}\right]_{cyto} \tag{78}$$

$$K_a = \left[1+\left(K_{da} \middle/ {}^{\theta_{MEM}(p)}\left[Ca^{2+}\right]_{cyto}\right)\right] \tag{79}$$

$$z = \frac{V_m F}{RT} \tag{80}$$

Membrane Ca^{2+} pump, J_{pCa}

$$^{p}J_{pCa} = \left(v_{vox}^{-1} g_{pCa} \ {}^{\theta_{MEM}(p)}\left[Ca^{2+}\right]_{cyto}\right) \middle/ \left(K_{mpCa} + {}^{\theta_{MEM}(p)}\left[Ca^{2+}\right]_{cyto}\right) \tag{81}$$

Background Ca^{2+} current

$$^{p}J_{Cab} = v_{vox}^{-1} g_{Cab}\left(V_m - E_{r,Ca}\right) \tag{82}$$

Table S7: Membrane flux parameters

Parameter	Description	Value
g_{NaCa}	Maximal flux rate of Sodium-Ca^{2+} exchanger	0.13041 $\mu m^3.\mu M.ms^{-1}$
K_{da}	Ca^{2+} scaling constant	0.11 μM
η	Voltage sensitivity coefficient	0.35
K_{sat}	Saturation constant	0.27
K_{mcai}	Intracellular Ca^{2+} constant	3.59 μM
K_{mcao}	Extracellular Ca^{2+} constant	1.3 mM
K_{mnai}	Intracellular Na^+ constant	12.3 mM
K_{mnao}	Extracellular Na^+ constant	87.5 mM
g_{pca}	Maximal flux rate of PMCA Ca^{2+} pump	4.795 $\times10^{-4}$ $\mu m^3.\mu M.ms^{-1}$
g_{cab}	Maximal flux rate of background Ca^{2+} current	6.39 $\times10^{-6}$ $\mu m^3.\mu M.ms^{-1}.mV^{-1}$
$[Na^+]_i$	Intracellular Na^+ concentration	7.95 mM
$[Na^+]_o$	Extracellular Na^+ concentration	136 mM

Complete Equations for the Reaction Terms

Thus, the reaction terms in each of the two diffusively coupled cytoplasm domains (cyto, rbSS) are described by:

$$\left.\begin{aligned}
{}^{n}\phi_{cyto} &= {}^{n}J_{SS}\left(v_{ss_vox}/v_{cyto_vox}\right) - {}^{n}J_{trpn} \\
{}^{n}\phi_{cyto} &= -({}^{q}J_{up} - {}^{q}J_{leak}) + {}^{n}J_{SS} - {}^{n}J_{trpn} \\
{}^{n}\phi_{cyto} &= {}^{p}J_{NaCa} + {}^{p}J_{pCa} + {}^{p}J_{Cab} + {}^{n}J_{SS} - {}^{n}J_{trpn} \\
{}^{n}\phi_{cyto} &= {}^{p}J_{NaCa} + {}^{p}J_{pCa} + {}^{p}J_{Cab} - ({}^{q}J_{up} - {}^{q}J_{leak}) + {}^{n}J_{SS} - {}^{n}J_{trpn}
\end{aligned}\right\}
\begin{aligned}
&\forall n \notin \theta_{mem}(p) \wedge n \notin \theta_{SR}(q) \\
&\forall n \notin \theta_{mem}(p) \wedge n \in \theta_{SR}(q) \\
&\forall n \in \theta_{mem}(p) \wedge n \notin \theta_{SR}(q) \\
&\forall n \in \theta_{mem}(p) \wedge n \in \theta_{SR}(q)
\end{aligned} \tag{83}$$

$$\left.\begin{aligned}
{}^{n}\phi_{rbSS} &= -{}^{n}J_{SS} \\
{}^{n}\phi_{rbSS} &= -{}^{n}J_{SS} + {}^{m}J_{ds}\left(v_{ds}/v_{rbSS_vox}\right)
\end{aligned}\right\}
\begin{aligned}
&\forall n \notin \theta(m) \\
&\forall n \in \theta(m)
\end{aligned} \tag{84}$$

Where p is given by the inverse map $\theta_{mem}{}^{-1}(n)$, q is given by the inverse map $\theta_{SR}{}^{-1}(n)$ and m is given by the inverse map $\theta^{-1}(n)$.

And in the nSR domain, for all voxels n which correspond to an nSR voxel q:

$$\left.\begin{aligned}
{}^{q}\phi_{nSR} &= \left({}^{q}J_{up} - {}^{q}J_{leak}\right)\left(v_{cyto_vox}/v_{nSR_vox}\right) \\
{}^{q}\phi_{nSR} &= \left({}^{q}J_{up} - {}^{q}J_{leak}\right)\left(v_{cyto_vox}/v_{nSR_vox}\right) + {}^{m}J_{jSR}\left(v_{jSR}/v_{nSR_vox}\right)
\end{aligned}\right\}
\begin{aligned}
&\forall q \notin \theta(m) \\
&\forall q \in \theta(m)
\end{aligned} \tag{85}$$

Where n is given by the map $\theta_{SR}(q)$ to define m from the inverse map $\theta^{-1}(n)$.

For computational efficiency, membrane, SR and dyad fluxes are computed only for voxels n given by the appropriate map; the reaction term is calculated by summing reaction terms given over individual loops of each set.

Ionic Model

The simplified ionic model uses currents from recent cell models (Colman et al [13], O'Hara-Rudy [14]) without the inclusion of additional factors such as phosphorylation. Modified equations are as follows:

All gates updated by:

$$y|_{t=t_{n+1}} = y_\infty - \left(y_\infty - y|_{t=t_n}\right)e^{-dt/\tau_y} \tag{86}$$

I_{Na}:

$$I_{Na} = 16.m^3.h.j\left(V_m - E_{Na}\right) \tag{87}$$

$$\alpha_m = 0.32\left(V_m + 47.13\right)\Big/\left(1 - e^{0.1\left(V_m + 47.13\right)}\right) \tag{88}$$

$$\beta_m = 0.08e^{-V_m/11} \tag{89}$$

$$\alpha_h = \left.\begin{matrix} 0.135e^{(V_m+80)/-6.8} \\ 0 \end{matrix}\right\} \begin{matrix} V_m < -40mV \\ V_m \geq -40mV \end{matrix} \tag{90}$$

$$\beta_h = \begin{matrix} 3.56e^{0.079V_m} + 310000e^{0.35V_m} \\ \left[0.13\left(1+e^{(V_m+10.66)/-11.1}\right)\right]^{-1} \end{matrix} \begin{cases} V_m < -40mV \\ V_m \geq -40mV \end{cases} \tag{91}$$

$$\alpha_j = \begin{matrix} \left(-127140e^{0.2444Vm} - 0.00003474e^{-0.04391Vm}\right)\left(\dfrac{V_m + 37.78}{1+e^{0.311(V_m+79.23)}}\right) \\ 0 \end{matrix} \begin{cases} V_m < -40mV \\ V_m \geq -40mV \end{cases} \tag{92}$$

$$\beta_j = \begin{matrix} 0.1212e^{-0.01052V_m} \Big/ \left(1+e^{-0.1378(V_m+40.14)}\right) \\ 0.3e^{2.525\times10^{-7}V_m} \Big/ \left(1+e^{-0.1(V_m+32)}\right) \end{matrix} \begin{cases} V_m < -40mV \\ V_m \geq -40mV \end{cases} \tag{93}$$

<u>*I_{to}*</u>:

$$I_{to} = 0.02.a.i.(V_m - E_k) \tag{94}$$

$$a_\infty = 1.0 \Big/ \left(1+e^{-(V_m-14.34)/14.82}\right) \tag{95}$$

$$\tau_a = 1.0515 \Big/ \left(\left[1.2089(1+e^{-(V_m-19.4099)/29.3814})\right]^{-1} + 3.5/1+e^{(V_m+100)/29.3814}\right) \tag{96}$$

$$i_\infty = 1.0 \Big/ \left(1+e^{(V_m+43.94)/5.711}\right) \tag{97}$$

$$\tau_{i,fast} = 4.562 + 1.0 \Big/ \left(0.3933e^{-(V_m+100)/100} + 0.08004e^{(V_m+50)/16.59}\right) \tag{98}$$

$$\tau_{i,slow} = 23.62 + 1.0 \Big/ \left(0.0014516e^{-(V_m+96.52)/59.05} + 1.78\times10^{-8}e^{(V_m+114.1)/8.079}\right) \tag{99}$$

$$A_{i,fast} = 1.0 \Big/ \left(1+e^{(V_m-213.6)/151.2}\right) \tag{100}$$

$$i = A_{i,fast}.i_{fast} + (1-A_{i,fast})i_{slow} \tag{101}$$

<u>*I_{Ks}*</u>:

$$I_{Ks} = 1.955\times10^{-3}.Ks_{Ca}.xs_1.xs_2.(V_m - E_k) \tag{102}$$

$$xs_{1,\infty} = 1.0 \Big/ \left(1.0+e^{-(V_m+11.60)/8.932}\right) \tag{103}$$

$$\tau_{xs1} = 817.3 + 1.0 \Big/ \left(2.32\times10^{-4}e^{(V_m+48.28)/17.80} + 0.001292e^{-(V_m+210.0)/230.0}\right) \tag{104}$$

$$xs_{2,\infty} = xs_{1,\infty} \tag{105}$$

$$\tau_{xs2} = 1.0 \Big/ \left(0.01e^{(V_m-50.0)/2.0.0} + 0.0193e^{-(V_m+66.54)/31.0}\right) \tag{106}$$

$$Ks_{Ca} = 1.0 + 0.6 \Big/ \left(1 + \left(\frac{3.8 \times 10^{-5}}{[Ca^{2+}]_{CYTO}}\right)^{1.4}\right) \tag{107}$$

I_{Kr}:

$$I_{Kr} = 0.03174.\sqrt{[K^+]_O / 5.4}.xr.rkr.(V_m - E_k) \tag{108}$$

$$xr_\infty = 1.0 \Big/ \left(1.0 + e^{-(V_m + 8.337)/6.789}\right) \tag{109}$$

$$\tau_{xr,fast} = 12.98 + 1.0 \Big/ \left(0.3652 e^{(V_m - 31.66)/3.869} + 4.123 \times 10^{-5} e^{-(V_m - 47.78)/20.38}\right) \tag{110}$$

$$\tau_{xr,slow} = 1.865 + 1.0 \Big/ \left(0.06629 e^{(V_m - 34.7)/7.355} + 1.128 \times 10^{-5} e^{-(V_m - 29.74)/25.94}\right) \tag{111}$$

$$A_{xr,fast} = 1.0 \Big/ \left(1.0 + e^{(V_m + 54.81)/38.21}\right) \tag{112}$$

$$xr = A_{xr,fast} xr_{fast} + (1 - A_{xr,fast}) xr_{slow} \tag{113}$$

$$rkr = \left(1.0 + e^{(V_m + 55.0)/75.0}\right)^{-1} \left(1.0 + e^{(V_m - 10)/30.0}\right)^{-1} \tag{114}$$

I_{K1}:

$$I_{K1} = 0.178398.\sqrt{[K^+]_O}.rk1.xk1.(V_m - E_k) \tag{115}$$

$$xk1_\infty = 1.0 \Big/ \left(1.0 + e^{-(V_m + 2.5538[K^+]_O + 144.59)/(1.5692[K^+]_O + 3.8115)}\right) \tag{116}$$

$$\tau_{xk1} = 122.2 \Big/ \left(e^{-(V_m + 127.2/20.36)} + e^{(V_m + 236.8)69.33}\right) \tag{117}$$

$$rk1 = 1.0 \Big/ \left(1.0 + e^{(V_m + 105.8 - 2.6[K^+]_O)/9.493}\right) \tag{118}$$

Initial Conditions

Table S8: Initial conditions of Ca^{2+} handling model (control pacing)

Variable	Initial value
$[Ca^{2+}]_{ds}$	0.08 µM
$[Ca^{2+}]_{rbSS}$	0.08 µM
$[Ca^{2+}]_{cyto}$	0.08 µM
$[Ca^{2+}]_{nSR}$	0.8 mM
$[Ca^{2+}]_{jSR}$	0.8 mM
CA	1.0
d_1, f_1, fca_1	1.0
V_m	-90 mV
P_0	1.667×10^{-3}
P_1	1.441×10^{-3}
P_2	2.692×10^{-3}
P_3	2.344×10^{-3}
N_0	9.904×10^{-3}
N_1	1.435×10^{-3}
$\left[TRPN_{Ca}^{L}\right]$	0.0181
$\left[TRPN_{Ca}^{H}\right]$	0.1305

References

1. Hinch R. A mathematical analysis of the generation and termination of calcium sparks. Biophys J. 2004 Mar;86(3):1293–307.
2. Restrepo JG, Weiss JN, Karma A. Calsequestrin-mediated mechanism for cellular calcium transient alternans. Biophys J. 2008 Oct;95(8):3767–89.
3. Nivala M, de Lange E, Rovetti R, Qu Z. Computational modeling and numerical methods for spatiotemporal calcium cycling in ventricular myocytes. Front Physiol. 2012;3:114.
4. Wagner J, Keizer J. Effects of rapid buffers on Ca2+ diffusion and Ca2+ oscillations. Biophys J. 1994 Jul;67(1):447–56.
5. Gaur N, Rudy Y. Multiscale modeling of calcium cycling in cardiac ventricular myocyte: macroscopic consequences of microscopic dyadic function. Biophys J. 2011 Jun 22;100(12):2904–12.
6. Gauthier LD, Greenstein JL, Winslow RL. Toward an integrative computational model of the Guinea pig cardiac myocyte. Front Physiol. 2012;3:244.
7. Rice JJ, Winslow RL, Hunter WC. Comparison of putative cooperative mechanisms in cardiac muscle: length dependence and dynamic responses. Am J Physiol. 1999 May;276(5 Pt 2):H1734-1754.
8. Stern MD, Song LS, Cheng H, Sham JS, Yang HT, Boheler KR, et al. Local control models of cardiac excitation-contraction coupling. A possible role for allosteric interactions between ryanodine receptors. J Gen Physiol. 1999 Mar;113(3):469–89.
9. Luo CH, Rudy Y. A dynamic model of the cardiac ventricular action potential. I. Simulations of ionic currents and concentration changes. Circ Res. 1994 Jun;74(6):1071–96.
10. Song Z, Ko CY, Nivala M, Weiss JN, Qu Z. Calcium-voltage coupling in the genesis of early and delayed afterdepolarizations in cardiac myocytes. Biophys J. 2015 Apr 21;108(8):1908–21.
11. Shiferaw Y, Watanabe MA, Garfinkel A, Weiss JN, Karma A. Model of intracellular calcium cycling in ventricular myocytes. Biophys J. 2003 Dec;85(6):3666–86.
12. Shannon TR, Wang F, Puglisi J, Weber C, Bers DM. A mathematical treatment of integrated Ca dynamics within the ventricular myocyte. Biophys J. 2004 Nov;87(5):3351–71.
13. Colman MA, Aslanidi OV, Kharche S, Boyett MR, Garratt C, Hancox JC, et al. Pro-arrhythmogenic effects of atrial fibrillation-induced electrical remodelling: insights from the three-dimensional virtual human atria. J Physiol. 2013 Sep 1;591(17):4249–72.
14. O'Hara T, Virág L, Varró A, Rudy Y. Simulation of the undiseased human cardiac ventricular action potential: model formulation and experimental validation. PLoS Comput Biol. 2011 May;7(5):e1002061.
