# Supplementary material for: A computational model of spatio-temporal cardiac intracellular calcium handling with realistic structure and spatial flux distribution from sarcoplasmic reticulum and t-tubule reconstructions
Source: PLoS Comput Biol. 2017 Aug 31;13(8):e1005714. doi: 10.1371/journal.pcbi.1005714 (PMC5597258; doi:10.1371/journal.pcbi.1005714)
Supplement: S1 Fig — (PDF) [file pcbi.1005714.s003.pdf]

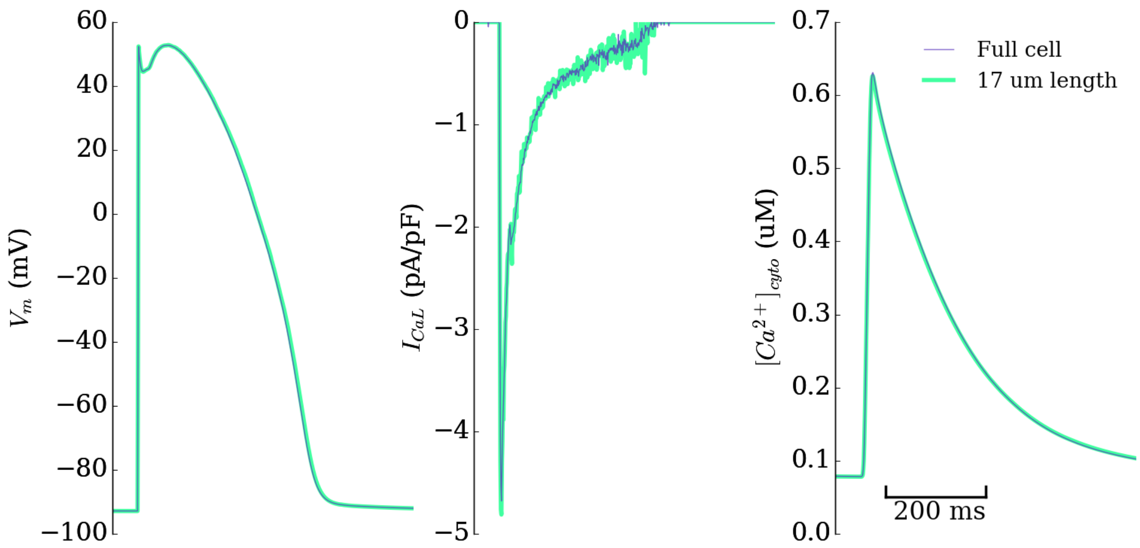

Fig 1: Comparison between the full-sized cell model and the cross-sectional portion cell model. Membrane potential (left), I_{CaL} (middle) and average Ca^{2+} transient (right) traces from the full length cell model (purple thin line) and a small portion of length 17 µm (green thick line).
