# Supplementary material for: A computational model of spatio-temporal cardiac intracellular calcium handling with realistic structure and spatial flux distribution from sarcoplasmic reticulum and t-tubule reconstructions
Source: PLoS Comput Biol. 2017 Aug 31;13(8):e1005714. doi: 10.1371/journal.pcbi.1005714 (PMC5597258; doi:10.1371/journal.pcbi.1005714)
Supplement: S2 Fig — (PDF) [file pcbi.1005714.s004.pdf]

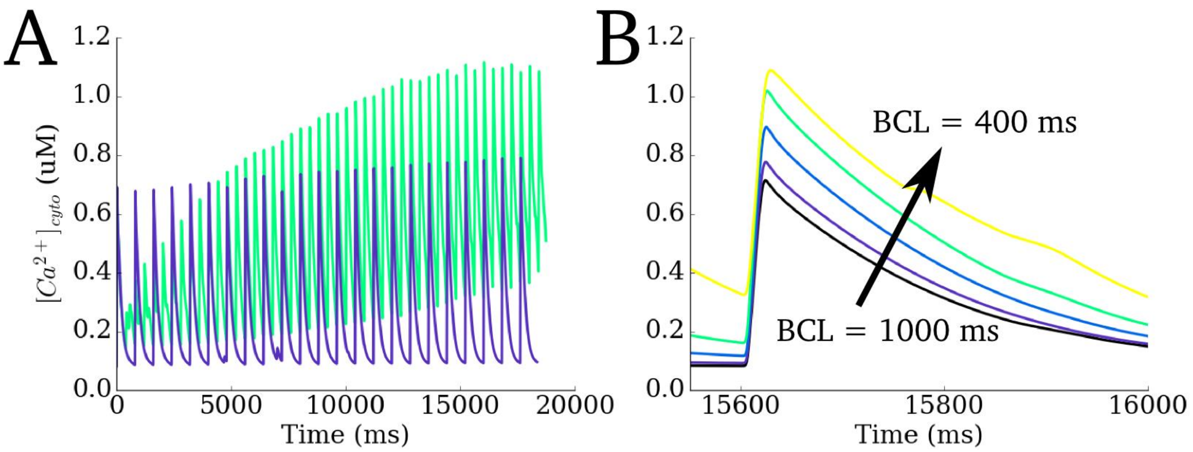

Fig 2: Rate-dependence of the intracellular Ca2+ transient. A – Ca^{2+} transient during long term pacing from initial conditions at BCL = 800 ms (purple) and 400 ms (green). B – Ca^{2+} transient for a single beat (steady state) for multiple BCLs.
