# Supplementary figures and images for: A computational model of spatio-temporal cardiac intracellular calcium handling with realistic structure and spatial flux distribution from sarcoplasmic reticulum and t-tubule reconstructions
Source: PLoS Comput Biol. 2017 Aug 31;13(8):e1005714. doi: 10.1371/journal.pcbi.1005714 (PMC5597258; doi:10.1371/journal.pcbi.1005714)

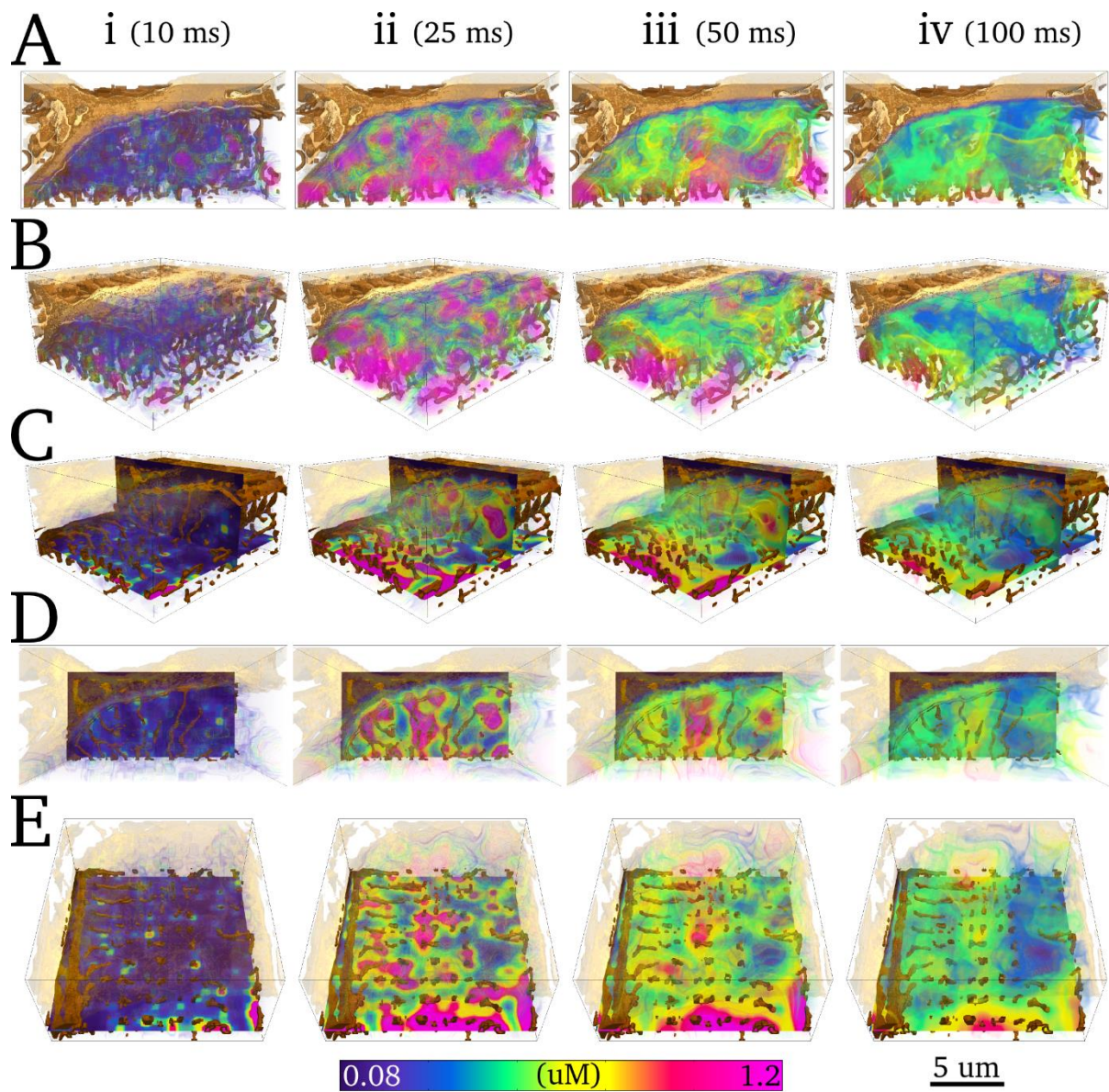

**Fig 3: Further renders of  $\text{Ca}^{2+}$  gradients in 3-D.**

Supplement: S3 Fig — (PDF) [file pcbi.1005714.s005.pdf]

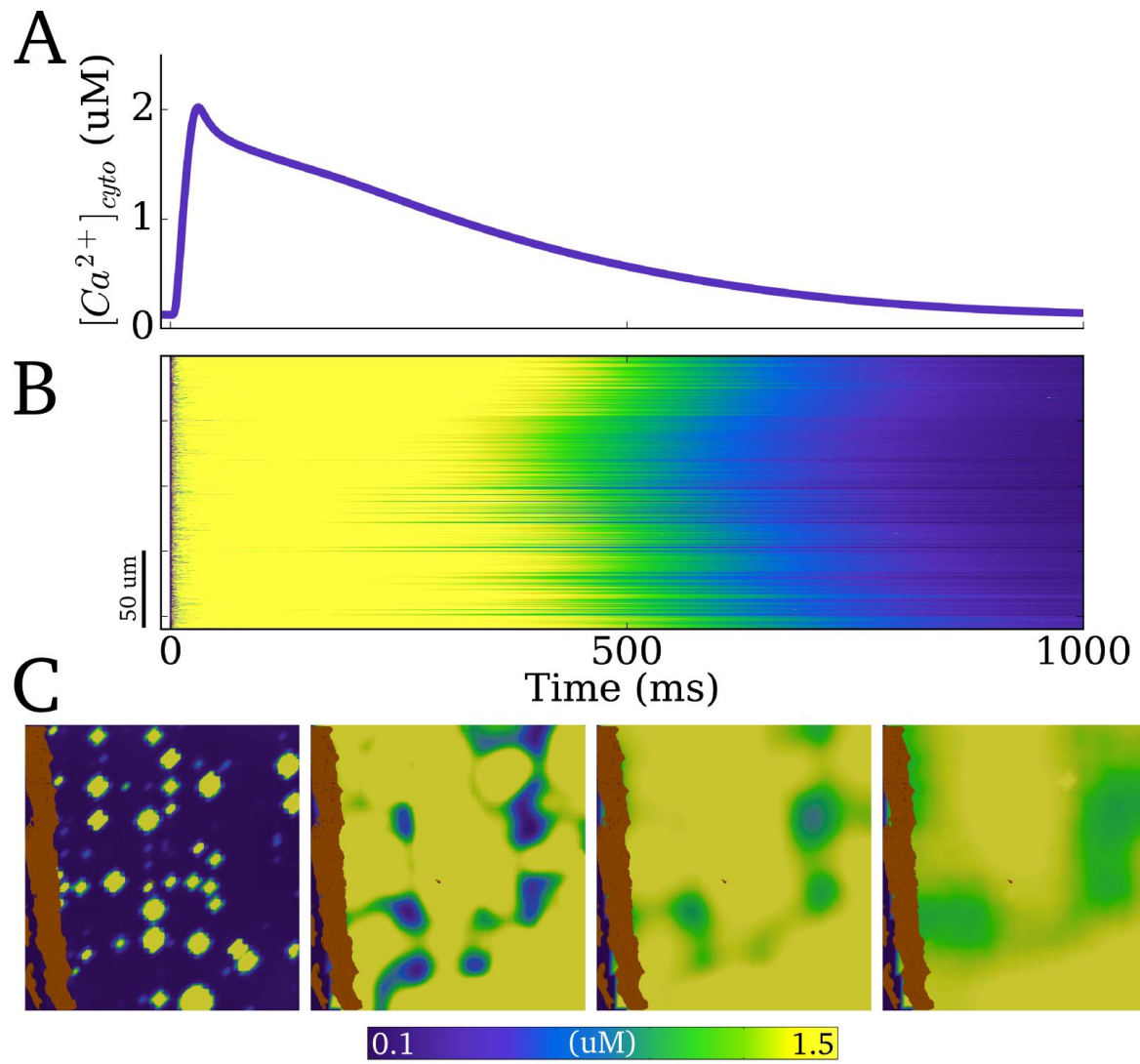

**Fig 5: Spatio-temporal dynamics with the Nivala et al 2012 model showing the same snapshots as in Fig 7.**

Supplement: S5 Fig — (PDF) [file pcbi.1005714.s007.pdf]
