# Supplementary material for: A computational model of spatio-temporal cardiac intracellular calcium handling with realistic structure and spatial flux distribution from sarcoplasmic reticulum and t-tubule reconstructions
Source: PLoS Comput Biol. 2017 Aug 31;13(8):e1005714. doi: 10.1371/journal.pcbi.1005714 (PMC5597258; doi:10.1371/journal.pcbi.1005714)
Supplement: S4 Fig — (PDF) [file pcbi.1005714.s006.pdf]

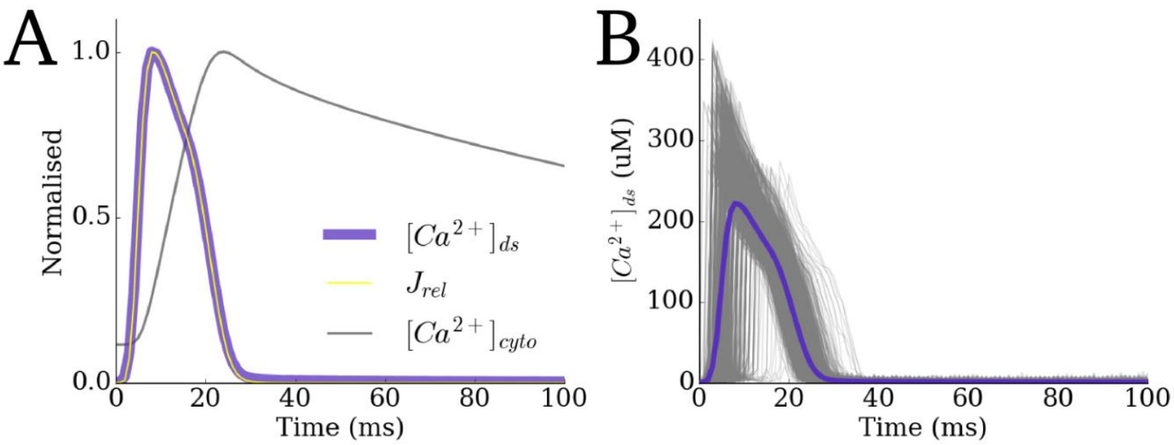

Fig 4: *J_{rel}* and single dyad dynamics. A – Whole cell averages for Ca^{2+} concentration in the dyadic cleft (purple) and cytoplasm (grey) and intracellular Ca^{2+} release, J_{rel} (yellow). B – Ca^{2+} concentration in multiple individual dyadic clefts (grey) with the average (purple).
