# Supplementary material for: A computational model of spatio-temporal cardiac intracellular calcium handling with realistic structure and spatial flux distribution from sarcoplasmic reticulum and t-tubule reconstructions
Source: PLoS Comput Biol. 2017 Aug 31;13(8):e1005714. doi: 10.1371/journal.pcbi.1005714 (PMC5597258; doi:10.1371/journal.pcbi.1005714)
Supplement: S6 Fig — (PDF) [file pcbi.1005714.s008.pdf]

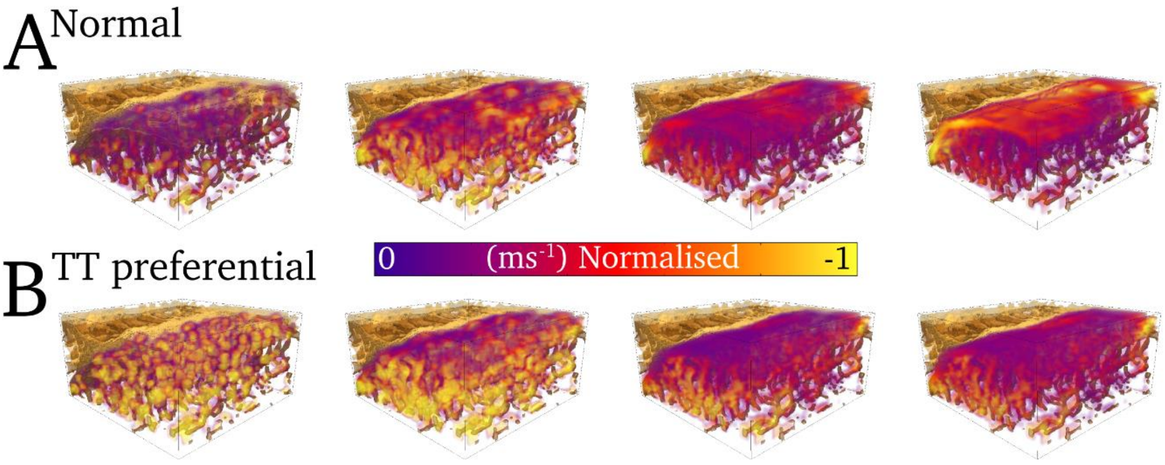

Fig 6: Snapshots of INaCa flux distribution in the even and TT-preferentially distributed models.
